# Supplementary material for: Impact of nonrandom selection mechanisms on the causal effect estimation for two-sample Mendelian randomization methods
Source: PLoS Genet. 2022 Mar 17;18(3):e1010107. doi: 10.1371/journal.pgen.1010107 (PMC8963545; doi:10.1371/journal.pgen.1010107)
Supplement: S1 Text — (PDF) [file pgen.1010107.s001.pdf]

S1 Text

Modeling assumptions

Let $G_1, G_2, ..., G_J$ be *J* genetic variants that are independent with each other. *X*, *Y* and *U* denote an exposure, an outcome and an unmeasured confounder, respectively. We assume that summary data is available on genetic associations with the exposure (beta-coefficient $\hat{\beta}_{X_j}$ and standard error σ_{X_j}) and with the outcome (beta-coefficient $\hat{\beta}_{Y_j}$ and standard error σ_{Y_j}) for each variant G_j.

Three core assumptions. A valid instrumental variable (IV) must satisfy the following three assumptions: i. Relevance – IV (G_j) is robustly associated with the exposure (*X*); ii. Exchangeability – IV (G_j) is not associated with any confounder (*U*) of the exposure–outcome relationship; iii. Exclusion restriction – IV (G_j) is independent of the outcome (*Y*) conditional on the exposure (*X*) and all confounders of the exposure-outcome relationship (i.e. the only path between the instrument and the outcome is via the exposure (*X*)).

Linear and homogeneity assumption. Estimating the average treatment effect (ATE) of an exposure (*X*) on an outcome (*Y*) in the full study population further requires additional sufficient conditions are linearity of the instrumental variable-exposure, instrumental variable-outcome and exposure–outcome relationships with no effect heterogeneity.

Monotonicity assumption. Monotonicity in the context of Mendelian randomization (MR) means that increasing the number of effect alleles for an individual can only increase the exposure from absent to present (or leave it constant), and can never decrease it. (We here define the effect allele as the exposure-increasing allele without loss of generality.) The analogue of ‘compliers’ in Mendelian randomization is individual who would have the exposure present if

they possess an exposure-increasing allele, but would not otherwise. The causal effect, which can be estimated in compliers, is known as the local average treatment effect (LATE) or the complier average causal effect (CACE) in the literature.

No sample overlap. Two sample MR requires two non-overlap samples to estimate causal effects. MR analyses based on summary data using instrument-exposure and instrument-outcome associations in the same sample or in partially overlapping samples may be prone to weak instrument bias and further bias the causal effect estimation. A simulation study suggest that bias due to sample overlap is a linear function of the proportion of overlap between the samples.
