# Supplementary material for: Impact of nonrandom selection mechanisms on the causal effect estimation for two-sample Mendelian randomization methods
Source: PLoS Genet. 2022 Mar 17;18(3):e1010107. doi: 10.1371/journal.pgen.1010107 (PMC8963545; doi:10.1371/journal.pgen.1010107)
Supplement: S2 Text — (PDF) [file pgen.1010107.s002.pdf]

S2 Text

Proof of Theorems

Theorem 1 The sufficient conditions for causal effect invariance under different selection mechanisms $\beta_{G_jY|S_2} / \beta_{G_jX|S_1} = \beta_{G_jY} / \beta_{G_jX}$ from two populations are:

(a) for each valid instrumental variable G_j, $S_1 \perp G_j$ or $S_1 \perp X \mid G_j$ in population I and $S_2 \perp G_j$ or $S_2 \perp Y \mid G_j$ in population II, respectively, or

(b) $G_j \perp Y \mid S_2$ and $G_j \perp Y$ for each valid instrumental variable in population II.

***Proof*:**

For simplicity, each variable is assumed to follow an normal distribution *N*(0,1).

When there is no selection, the causal effect of *X* on *Y* is

$$\frac{\beta_{GY}}{\beta_{GX}} = \frac{\partial E(Y \mid G)}{\partial G} / \frac{\partial E(X \mid G)}{\partial G}.$$

The causal effect with selection is

$$\frac{\beta_{GY|S_2}}{\beta_{GX|S_1}} = \frac{\partial E(Y \mid G, S_2)}{\partial G} / \frac{\partial E(X \mid G, S_1)}{\partial G}.$$

The partial regression coefficient $\beta_{GX|S_1}$ can be estimated by $\frac{Sd_X}{Sd_G} \frac{\rho_{GX} - \rho_{XS_1}\rho_{GS_1}}{1-\rho_{GS_1}^2}$, where Sd is the standard deviation and ρ is the correlation coefficient. For example, Sd_X is the standard deviation of *X* and ρ_{GX} is the correlation coefficient between G and X. Because both X and G follows standard normal distributions, $Sd_X = Sd_G = 1$, i.e.

$$\beta_{GX|S_1} = \frac{\rho_{GX} - \rho_{XS_1}\rho_{GS_1}}{1-\rho_{GS_1}^2}.$$

Furthermore, $\rho_{GS_1} = \frac{\text{cov}(G, S_1)}{Sd_G Sd_{S_1}} = \frac{E(GS_1) - E(G)E(S_1)}{Sd_G Sd_{S_1}} = E(GS_1)$.

Assume that $E(G)=E(\rho_{GX}X)$ and $E(S_1)=E(\rho_{XS_1}X)$,

$E(GS_1)=E(\rho_{GX}\rho_{XS_1}X^2)=\rho_{GX}\rho_{XS_1}E(X^2)=\rho_{GX}\rho_{XS_1}$.

Thus, $\beta_{GX|S_1}=\dfrac{\rho_{GX}-\rho_{XS_1}\rho_{GS_1}}{1-\rho_{GS_1}^2}=\dfrac{\rho_{GX}-\rho_{XS_1}{}^2\rho_{GX}}{1-\rho_{GX}{}^2\rho_{XS_1}{}^2}$.

In addition, $\beta_{GX}=\rho_{GX}$, $\beta_{XS_1}=\rho_{XS_1}$, $\beta_{GS_1}=\rho_{GS_1}$ due to the normal distributions of S_1, G and X. Therefore, $\beta_{GX|S_1}=\dfrac{\rho_{GX}-\rho_{XS_1}\rho_{GS_1}}{1-\rho_{GS_1}^2}=\dfrac{\beta_{GX}-\beta_{XS_1}{}^2\beta_{GX}}{1-\beta_{GX}{}^2\beta_{XS_1}{}^2}=\dfrac{\beta_{GX}(1-\beta_{XS_1}{}^2)}{1-\beta_{GX}{}^2\beta_{XS_1}{}^2}$.

And yields the bias $\beta_{GX|S_1}-\beta_{GX}=\dfrac{\beta_{GX}\beta_{XS_1}{}^2(\beta_{GX}{}^2-1)}{1-\beta_{GX}{}^2\beta_{XS_1}{}^2}$.

The bias is zero if $S_1\perp G$, that is, $\beta_{GX}\beta_{XS_1}=0$.

In this situation, we can obtain $E(X\mid G,S_1=1)$=$E(X\mid G)$ from $\beta_{GX|S_1}=\beta_{GX}$.

If $S_1\perp X\mid G$, $E(X\mid G,S_1=1)$=$E(X\mid G)$ can also be obtained.

Thus when $S_1\perp G$ or $S_1\perp X\mid G$, $E(X\mid G,S_1=1)$=$E(X\mid G)$. Similarly, when $S_2\perp G$ or $S_2\perp Y\mid G$, we have $E(Y\mid G,S_2=1)$=$E(Y\mid G)$. Then we can obtain

$$\frac{\beta_{GY|S_2}}{\beta_{GX|S_1}}=\frac{\partial E(Y\mid G,S_2=1)}{\partial G}/\frac{\partial E(X\mid G,S_1=1)}{\partial G}=\frac{\partial E(Y\mid G)}{\partial G}/\frac{\partial E(X\mid G)}{\partial G}=\frac{\beta_{GY}}{\beta_{GX}}.$$

On the other hand, if $G\perp Y\mid S_2$, we have $\dfrac{\partial E(Y\mid G,S_2=1)}{\partial G}=0$. If $G\perp Y$, then $\dfrac{\partial E(Y\mid G)}{\partial G}=0$.

We have $\dfrac{\beta_{GY|S_2}}{\beta_{GX|S_1}}=\dfrac{\partial E(Y\mid G,S_2=1)}{\partial G}/\dfrac{\partial E(X\mid G,S_1=1)}{\partial G}=0=\dfrac{\partial E(Y\mid G)}{\partial G}/\dfrac{\partial E(X\mid G)}{\partial G}=\dfrac{\beta_{GY}}{\beta_{GX}}$.

The possible causal diagrams are as follows.

□

When exposure and outcome are binary, traditional MR methods can be used to judge whether there is a causal effect, but cannot estimate causal effect accurately. In this case, Wald

ratio can be written as $\log(OR_{G_jY|S_2=1})/\log(OR_{G_jX|S_1=1})$. In other words, beta-coefficients in linear regression are replaced by log(OR)-coefficients in logistic regression. The model of underlying binary variables should be:

$$\begin{aligned}\log it[P(Y \mid X, U, G_1, ..., G_J)] &= \theta X + U + \sum_{j=1}^{J} \gamma_j G_j \\ \log it[P(X \mid U, G_1, ..., G_J)] &= \sum_{j=1}^{J} \alpha_j G_j + U\end{aligned}.$$

We also give sufficient conditions for invariance of causal relationship using two-sample MR method on the scale of OR in Theorem 2. Due to the non-collapsibility, $S_1 \perp G_j$ and $S_2 \perp G_j$ in condition (a) are replaced by $S_1 \perp G_j \mid X$ and $S_2 \perp G_j \mid Y$, respectively. In comparison with the Theorem 1, OR can avoid the influence of selection bias of outcome-dependent, especially in case-control study design. The differences for the DAGs satisfying Theorem 2 are that selection depending on unmeasured confounder no more satisfy the condition (a) in both samples. Instead, selection depending on exposure in sample I and outcome in sample II satisfy condition (a) in Theorem 2.

Theorem 2 The sufficient conditions for invariance of causal relationship $\log(OR_{G_jY|S_1})/\log(OR_{G_jX|S_2}) = \log(OR_{G_jY})/\log(OR_{G_jX})$ from two populations with selection are:

(a) for each valid instrumental variable G_j, $S_1 \perp G_j \mid X$ or $S_1 \perp X \mid G_j$ in population I and $S_2 \perp G_j \mid Y$ or $S_2 \perp Y \mid G_j$ in population II, respectively, or

(b) $G_j \perp Y \mid S_2$ and $G_j \perp Y$ for each valid instrumental variable in population II.

***Proof*:**

(a) The causal effect $\log(OR_{GY|S_2})/\log(OR_{GX|S_1})$ can be written as

$$\log \frac{P(Y=1 \mid G=1, S_2=1)P(Y=0 \mid G=0, S_2=1)}{P(Y=1 \mid G=0, S_2=1)P(Y=0 \mid G=1, S_2=1)} / \log \frac{P(X=1 \mid G=1, S_1=1)P(X=0 \mid G=0, S_1=1)}{P(X=1 \mid G=0, S_1=1)P(X=0 \mid G=1, S_1=1)}$$

For sample I, the $OR_{GX|S_1}$ can be written as

$$OR_{GX|S_1}=\frac{P(X=1|G=1,S_1=1)P(X=0|G=0,S_1=1)}{P(X=1|G=0,S_1=1)P(X=0|G=1,S_1=1)}$$
$$=\frac{\frac{P(X=1,G=1,S_1=1)}{P(G=1,S_1=1)}\frac{P(X=0,G=0,S_1=1)}{P(G=0,S_1=1)}}{\frac{P(X=1,G=0,S_1=1)}{P(G=0,S_1=1)}\frac{P(X=0,G=1,S_1=1)}{P(G=1,S_1=1)}}.$$
$$=\frac{\frac{P(G=1,S_1=1|X=1)P(X=1)}{P(G=1)P(S_1=1|G=1)}\frac{P(G=0,S_1=1|X=0)P(X=0)}{P(G=0)P(S_1=1|G=0)}}{\frac{P(G=0,S_1=1|X=1)P(X=1)}{P(G=0)P(S_1=1|G=0)}\frac{P(G=1,S_1=1|X=0)P(X=0)}{P(G=1)P(S_1=1|G=1)}}$$

Because of $S_1\perp G|X$, $P(G,S_1|X)=P(S_1|X)P(G|X)$ can be achieved. Then, we can obtain that

$$OR_{GX|S_1}=\frac{\frac{P(G=1|X=1)P(S_1=1|X=1)P(X=1)}{P(G=1)P(S_1=1|G=1)}\frac{P(G=0|X=0)P(S_1=1|X=0)P(X=0)}{P(G=0)P(S_1=1|G=0)}}{\frac{P(G=0|X=1)P(S_1=1|X=1)P(X=1)}{P(G=0)P(S_1=1|G=0)}\frac{P(G=1|X=0)P(S_1=1|X=0)P(X=0)}{P(G=1)P(S_1=1|G=1)}}$$

Thus, $$OR_{GX|S_1}=\frac{\frac{P(G=1,X=1)}{P(G=1)}\frac{P(G=0,X=0)}{P(G=0)}}{\frac{P(G=0,X=1)}{P(G=0)}\frac{P(G=1,X=0)}{P(G=1)}}=\frac{P(X=1|G=1)P(X=0|G=0)}{P(X=1|G=0)P(X=0|G=1)}=OR_{GX}.$$

Thus, for population I, we can obtain that $OR_{GX|S_1}=OR_{GX}$ due to $S_1\perp G|X$.

For population I, the $OR_{GX|S_1}$ can be written as

$$OR_{GX|S_1}=\frac{P(X=1|G=1,S_1=1)P(X=0|G=0,S_1=1)}{P(X=1|G=0,S_1=1)P(X=0|G=1,S_1=1)}.$$

Because of $S_1\perp X|G$, we can obtain that

$$OR_{GX|S_1}=\frac{P(X=1|G=1,S_1=1)P(X=0|G=0,S_1=1)}{P(X=1|G=0,S_1=1)P(X=0|G=1,S_1=1)}=\frac{P(X=1|G=1)P(X=0|G=0)}{P(X=1|G=0)P(X=0|G=1)}=OR_{GX}.$$

Thus, for population I, we can obtain that $OR_{GX\bullet S_1}=OR_{GX}$ due to $S_1\perp X|G$.

Similarly, the $OR_{GY\bullet S_2}$ can be written as

$$OR_{GY|S_2} = \frac{P(X=1|G=1,S_1=1)P(X=0|G=0,S_1=1)}{P(X=1|G=0,S_1=1)P(X=0|G=1,S_1=1)}$$

$$= \frac{\frac{P(G=1,S_2=1|Y=1)P(Y=1)}{P(G=1)P(S_2=1|G=1)} \frac{P(G=0,S_2=1|Y=0)P(Y=0)}{P(G=0)P(S_2=1|G=0)}}{\frac{P(G=0,S_2=1|Y=1)P(Y=1)}{P(G=0)P(S_2=1|G=0)} \frac{P(G=1,S_2=1|X=0)P(Y=0)}{P(G=1)P(S_2=1|G=1)}} .$$

Because of $S_2 \perp G|Y$, $P(G,S_2|Y) = P(S_2|Y)P(G|Y)$ can be achieved. Then, we can obtain that

$$OR_{GY|S_2} = \frac{\frac{P(G=1|Y=1)P(S_2=1|Y=1)P(Y=1)}{P(G=1)P(S_2=1|G=1)} \frac{P(G=0|Y=0)P(S_2=1|Y=0)P(Y=0)}{P(G=0)P(S_2=1|G=0)}}{\frac{P(G=0|Y=1)P(S_2=1|Y=1)P(Y=1)}{P(G=0)P(S_2=1|G=0)} \frac{P(G=1|Y=0)P(S_2=1|Y=0)P(Y=0)}{P(G=1)P(S_2=1|G=1)}} .$$

Thus, $OR_{GY|S_2} = \frac{\frac{P(G=1,Y=1)}{P(G=1)} \frac{P(G=0,Y=0)}{P(G=0)}}{\frac{P(G=0,Y=1)}{P(G=0)} \frac{P(G=1,Y=0)}{P(G=1)}} = \frac{P(Y=1|G=1)P(Y=0|G=0)}{P(Y=1|G=0)P(Y=0|G=1)} = OR_{GY}$.

Thus, for population II, we can obtain that $OR_{GY\bullet S_2} = OR_{GY}$ due to $S_2 \perp G|Y$.

For population II, the $OR_{GY\bullet S_2}$ can be written as

$$OR_{GY|S_2} = \frac{P(Y=1|G=1,S_2=1)P(Y=0|G=0,S_2=1)}{P(Y=1|G=0,S_2=1)P(Y=0|G=1,S_2=1)} .$$

Because of $S_2 \perp G|Y$, we can obtain that

$$OR_{GY|S_2} = \frac{P(Y=1|G=1,S_2=1)P(Y=0|G=0,S_2=1)}{P(Y=1|G=0,S_2=1)P(Y=0|G=1,S_2=1)} = \frac{P(Y=1|G=1)P(Y=0|G=0)}{P(Y=1|G=0)P(Y=0|G=1)} = OR_{GY} .$$

Thus, for population II, we can obtain that $OR_{GY|S_2} = OR_{GY}$ due to $S_2 \perp Y|G$.

Therefore, the causal effect $\log(OR_{GY|S_2}) / \log(OR_{GX|S_1})$ is s-recoverable from two samples with selection.

(b) Because $G \perp Y|S_2$, and $G \perp Y$ for each valid instrumental variable in population II, we can obtain that $\log(OR_{GY|S_2}) = 0$. Thus,

$$\log(OR_{G_jY|S_1}) / \log(OR_{G_jX|S_2}) = \log(OR_{G_jY}) / \log(OR_{G_jX}) .$$

□

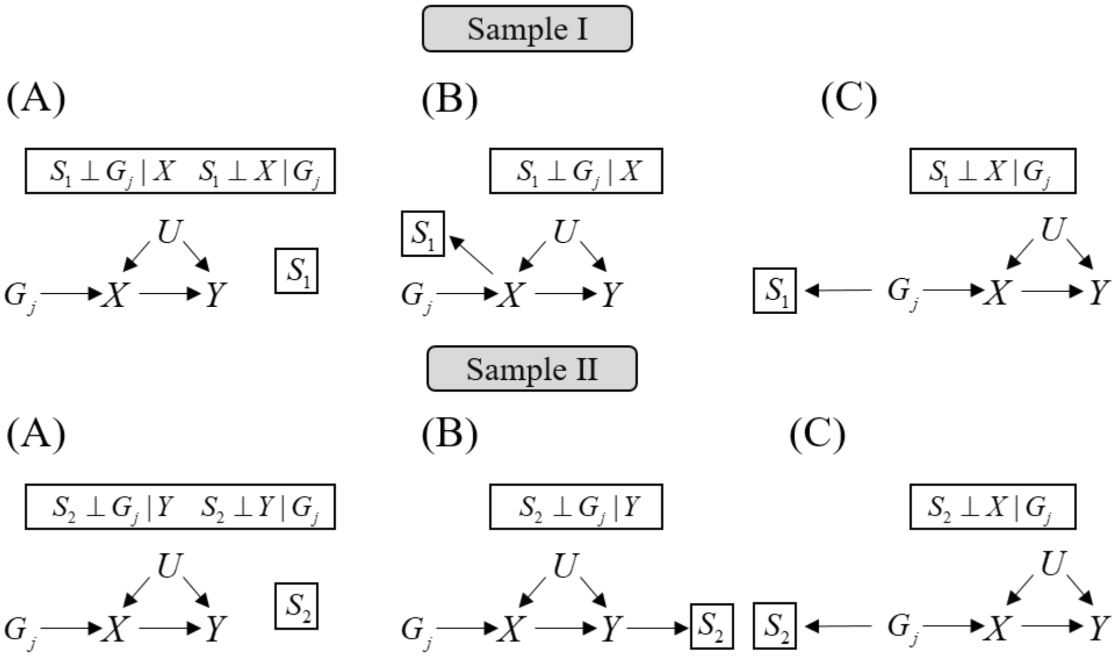

Fig A. The possible causal diagrams for condition (a) of Theorem 2.

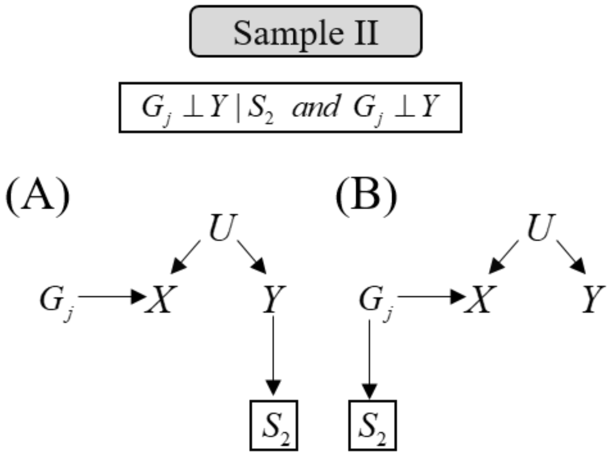

Fig B. The possible causal diagrams for condition (b) of Theorem 2.
