# Supplementary material for: Impact of nonrandom selection mechanisms on the causal effect estimation for two-sample Mendelian randomization methods
Source: PLoS Genet. 2022 Mar 17;18(3):e1010107. doi: 10.1371/journal.pgen.1010107 (PMC8963545; doi:10.1371/journal.pgen.1010107)
Supplement: S3 Text — (PDF) [file pgen.1010107.s003.pdf]

S3 Text

Eight pleiotropy-robust methods in simulation

In the case of multiple genetic variants, if all the genetic variants satisfy the IV assumptions, two stage least square is equal to inverse-variance weighted (IVW) method, which is a fixed-effect meta-analysis. In IVW, the IV-specific causal estimates $\hat{\theta}_j$ are the study-specific estimates, and the weights are the inverse-variance weights. The causal estimate from the IVW method ($\hat{\beta}_{IVW}$) is calculated by the following equation:

$$\hat{\beta}_{IVW} = \frac{\sum_{j=1}^{J} \hat{\theta}_j se(\hat{\theta}_j)^{-2}}{\sum_{j=1}^{J} se(\hat{\theta}_j)^{-2}} = \frac{\sum_{j=1}^{J} \hat{\beta}_{X_j} \hat{\beta}_{Y_j} \sigma_{Y_j}^{-2}}{\sum_{j=1}^{J} \hat{\beta}_{X_j} \sigma_{Y_j}^{-2}}.$$

The IVW estimate can also be obtained by weighted regression of the genetic associations with the outcome on the genetic associations with the exposure:

$$\hat{\beta}_{Y_j} = \theta\hat{\beta}_{X_j} + \varepsilon_j, \varepsilon_j \sim N(0, \sigma_{Y_j}^2)$$

If there are selections depending on *X* or *Y*, a non-causal pathway between *G* and *Y* via *U* (G→X←U→Y or X→Y←U) will be unlocked. This directly leads violations of the assumption of MR and causes the bias of IVW estimation. Nowadays, many of the premiere MR studies feature new instrument-based estimators that do not, strictly speaking, require that all proposed instruments are valid instruments. We wonder that to what extent pleiotropy-robust methods can solve selection bias. We proceed to introduce the different robust two sample MR methods we considered in later simulation: consensus methods, regression-based methods, likelihood-based methods and outlier-robust methods.

Consensus methods. Slob E and Burgess S [1] indicated that a consensus method is one that takes its causal estimate as a summary measure of the distribution of the ratio estimates, including two methods: the median method and the mode based estimate (MBE) method. Firstly,

the median method, requires up to 50% of the variants are valid, which is referred to 'majority valid' assumption. The simple median methods take the median of the ratio estimate $\hat{\theta}_j$. A weighted version takes the median from the ratio estimates in which genetic variants with more precise ratio estimates receive more weight. The MBE method requires the Zero Modal Pleiotropy Assumption (ZEMPA), which is that, out of all the different values taken by ratio estimates θ_j in large samples, the true causal effect is the value taken for the largest number of genetic variants. Then it constructs a smoothed density function by summing normal densities, which is drawn for each genetic variant centered at its ratio estimate $\hat{\theta}_j$:

$$f(x) = \frac{1}{\psi\sqrt{2\pi}} \sum_{j=1}^{L} w_j \exp\left[-\frac{1}{2}\left(\frac{x-\hat{\theta}_j}{\psi} \right)^2 \right],$$

where ψ is the smoothing bandwidth parameter. The causal effect estimate is the value of x that maximizes $f(x)$: $f(\hat{\beta}_{MBE}) = \max[f(x)]$.

Regression-based methods. MR-Egger allows the proposed instruments to be invalid as long as additional assumptions hold, including the Instrument Strength Independent of Direct Effect (InSIDE) assumption that the strength of the biasing pathway is independent of the strength of the proposed instrument–treatment relation. It is similarly to the IVW method, except that the regression model contains an intercept term θ_0:

$$\hat{\beta}_{Y_j} = \theta_0 + \theta\hat{\beta}_{X_j} + \varepsilon_j, \varepsilon_j \sim N(0, \sigma^2_{Y_j}).$$

The intercept will differ from zero when either the average pleiotropic effect is not zero, or the InSIDE assumption is violated. MR-robust method improves the robustness of IVW when some variants are outliers. In this method, M-estimation is used combined with Tukey's biweight loss function, which is a truncated quadratic function. This can limit the degree of which an outlier

contributes to the analysis.

Likelihood-based methods. The contamination mixture method assumes that only some of the genetic variants are valid IVs. This method constructs a likelihood function from the ratio estimates ($\hat{\theta}_j$). If a variant is a valid instrument, then $\hat{\theta}_j \sim N(\theta, se(\hat{\theta}_j)^2)$. If a variant is not a valid instrument, then $\hat{\theta}_j \sim N(0, \psi^2 + se(\hat{\theta}_j)^2)$. This parameter is specified by the analyst. Then this method first constructs a profile likelihood as a function of θ , and then maximizing this function with respect to θ . The value of θ that maximizes the profile likelihood is the causal estimate. The MR-Robust Adjusted Profile Score (RAPS) method provides a maximum profile likelihood estimator with provable consistency and asymptotic normality by adjusting the profile score. In this method, the pleiotropic effects are assumed to be normally distributed about zero with unknown variance. To provide further robustness to outliers, either Tukey's biweight loss function or Huber's loss function can be used.

Outlier-robust methods. In MR-Lasso, the MR-Egger regression model is augmented by adding a lasso penalty term. We minimize:

$$\sum_{j=1}^{J} \sigma_{Y_j}^{-2} (\hat{\beta}_{Y_j} - \theta_{0j} - \theta \hat{\beta}_{X_j})^2 + \lambda \sum_{j=1}^{J} | \theta_{0j} |$$

where λ is a tuning parameter. The intercept term θ_{0j} represents the pleiotropy effect on the outcome, and is equal to zero for a valid IV and non-zero for an invalid IV. These two methods both remove invalid variants from the analysis and will be valuable when there is a small number of invalid genetic variants.

Reference

1. Slob E, Burgess S. A comparison of robust mendelian randomization methods using summary data. bioRxiv [Preprint]. 2019. doi: http://dx.doi.org/10.1101/577940
