# Supplementary material for: Impact of nonrandom selection mechanisms on the causal effect estimation for two-sample Mendelian randomization methods
Source: PLoS Genet. 2022 Mar 17;18(3):e1010107. doi: 10.1371/journal.pgen.1010107 (PMC8963545; doi:10.1371/journal.pgen.1010107)
Supplement: S4 Text — (PDF) [file pgen.1010107.s004.pdf]

S4 Text

DAG for application

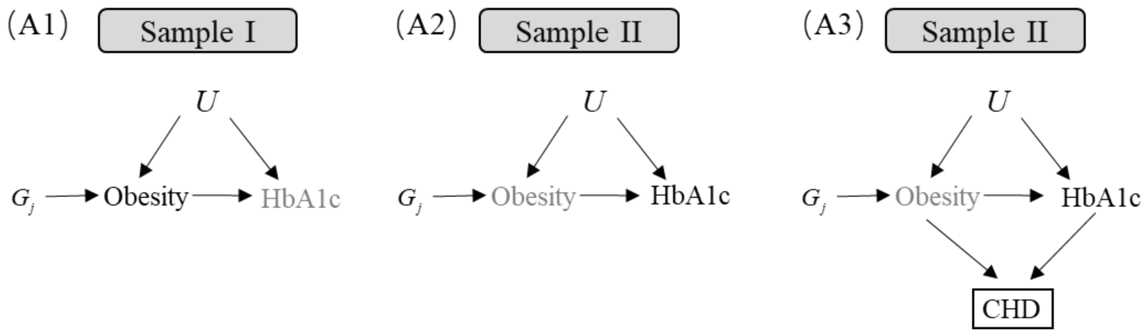

Fig A. The realistic causal diagram for the application. (A1) is DAG for sample I. (A2) and (A3) are DAGs for sample II in general population and CHD patients respectively.
