# Supplementary material for: Impact of nonrandom selection mechanisms on the causal effect estimation for two-sample Mendelian randomization methods
Source: PLoS Genet. 2022 Mar 17;18(3):e1010107. doi: 10.1371/journal.pgen.1010107 (PMC8963545; doi:10.1371/journal.pgen.1010107)
Supplement: S5 Text — (PDF) [file pgen.1010107.s005.pdf]

S5 Text

Simulation results of eight Pleiotropy-robust MR Methods with 100 valid genetic variants in scenario 1

We further investigate the estimations, Standard errors (SEs), type I error rates and statistic power when the number of genetic variants are 100 under different selection mechanisms while varying across selection effects of X, Y or G on selection (S) in scenario 1 (Figs A-F). The results are similarly with 50 variants.

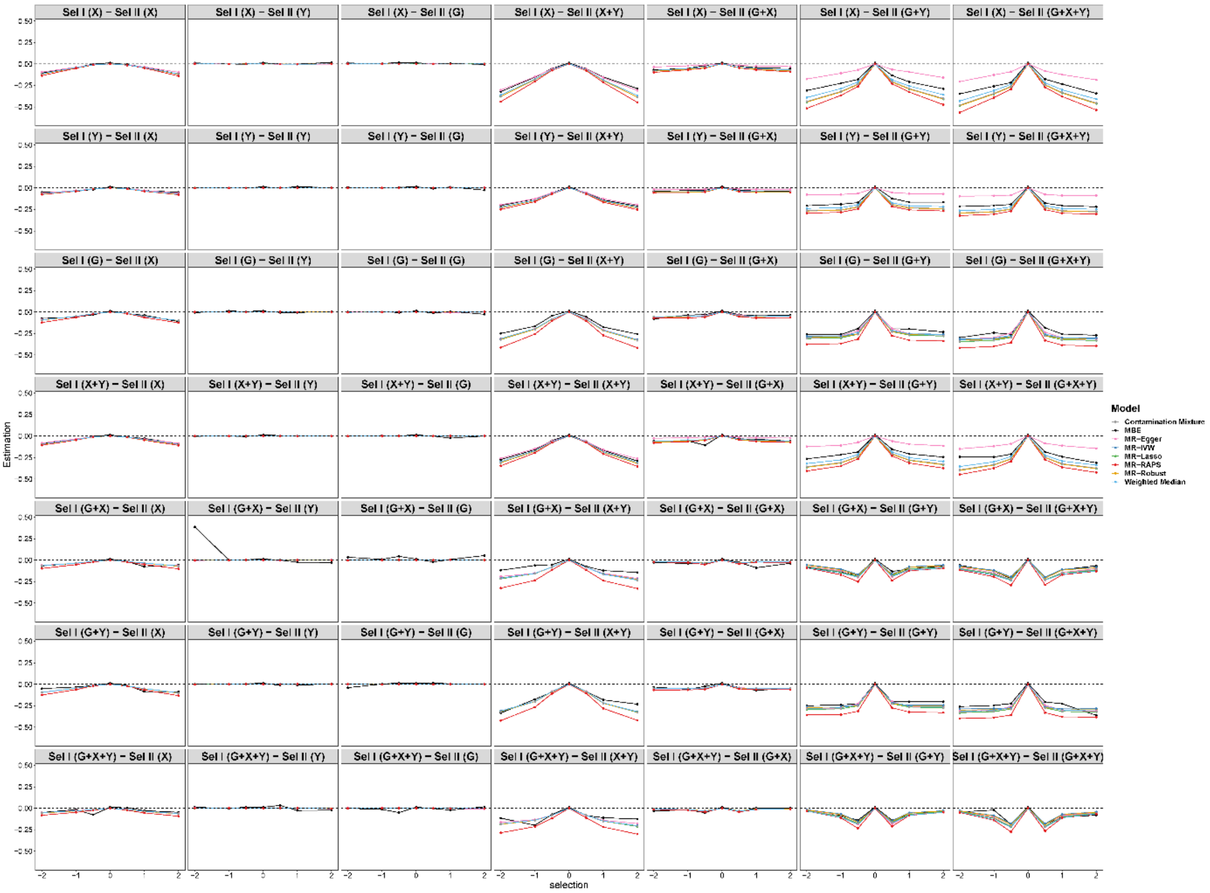

Fig A. Simulation results for causal estimations of eight Pleiotropy-robust MR Methods varying across selection effect from -2 to 2 under different selection mechanisms with Null causal effect in scenario 1 (100 genetic variants). Sel I and II represent selection in sample I and sample II, respectively.

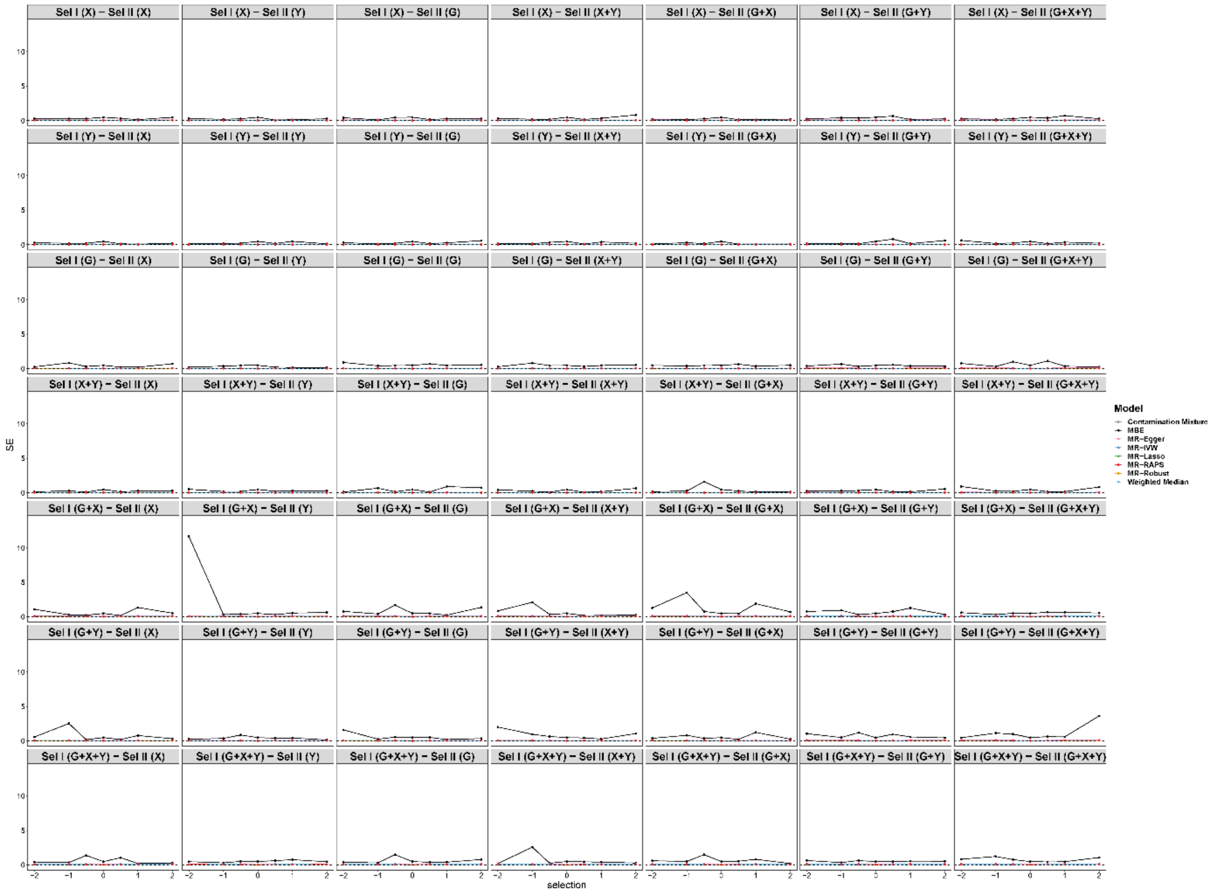

Fig B. Simulation results for SEs of eight Pleiotropy-robust MR Methods varying across selection effect from -2 to 2 under different selection mechanisms with Null causal effect in scenario 1 (100 genetic variants).

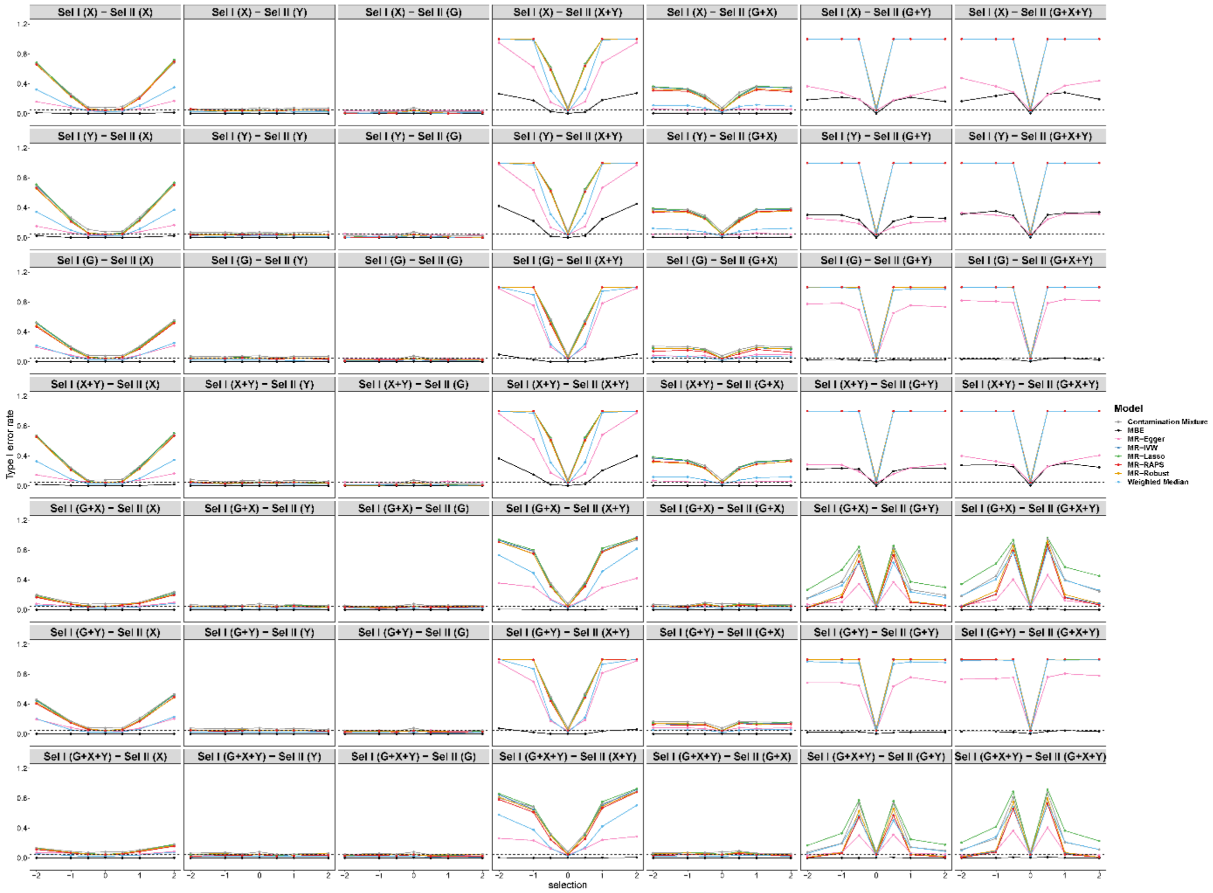

Fig C. Simulation results for type I error rates of eight Pleiotropy-robust MR Methods varying across selection effect from -2 to 2 under different selection mechanisms with Null causal effect in scenario 1 (100 genetic variants).

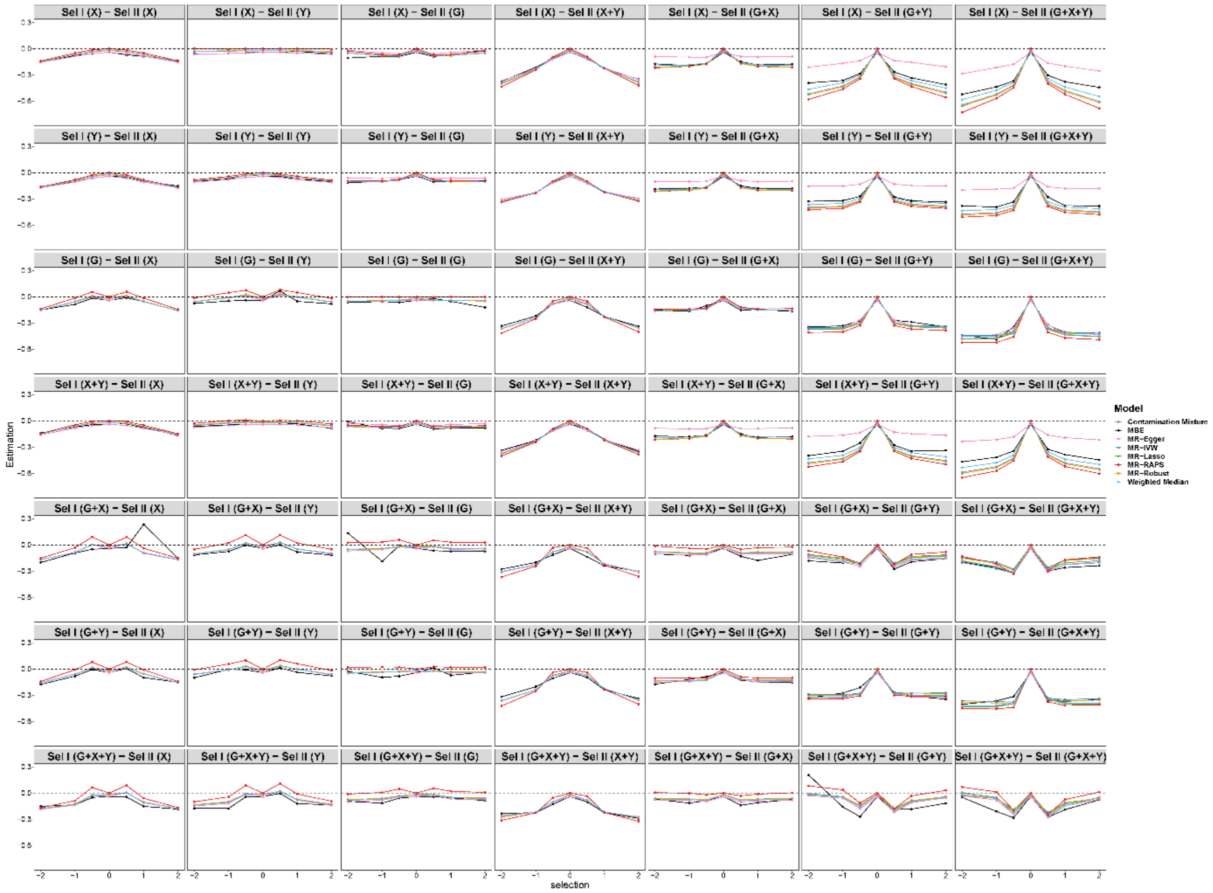

Fig D. Simulation results for causal estimations of eight Pleiotropy-robust MR Methods varying across selection effect from -2 to 2 under different selection mechanisms with Positive causal effect in scenario 1 (100 genetic variants).

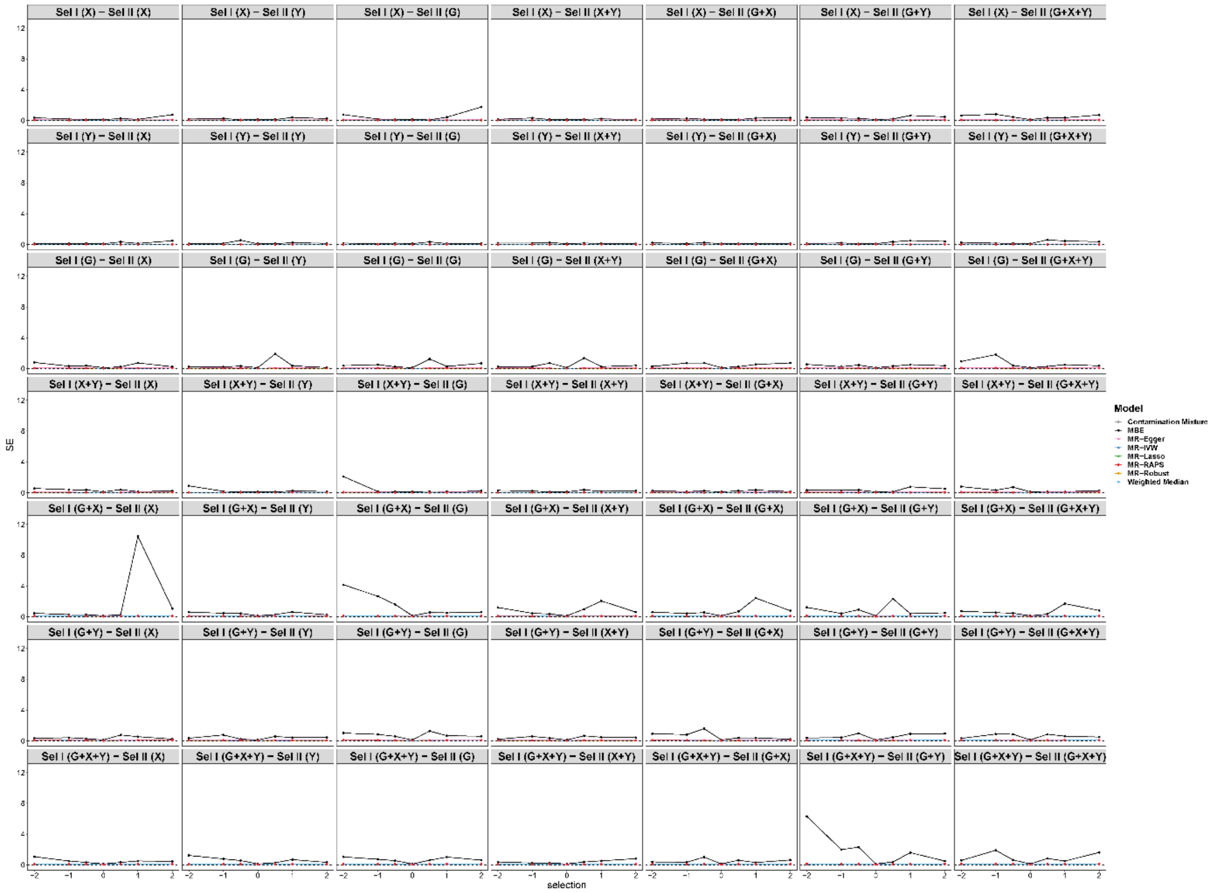

Fig E. Simulation results for SEs of eight Pleiotropy-robust MR Methods varying across selection effect from -2 to 2 under different selection mechanisms with Positive causal effect in scenario 1 (100 genetic variants).

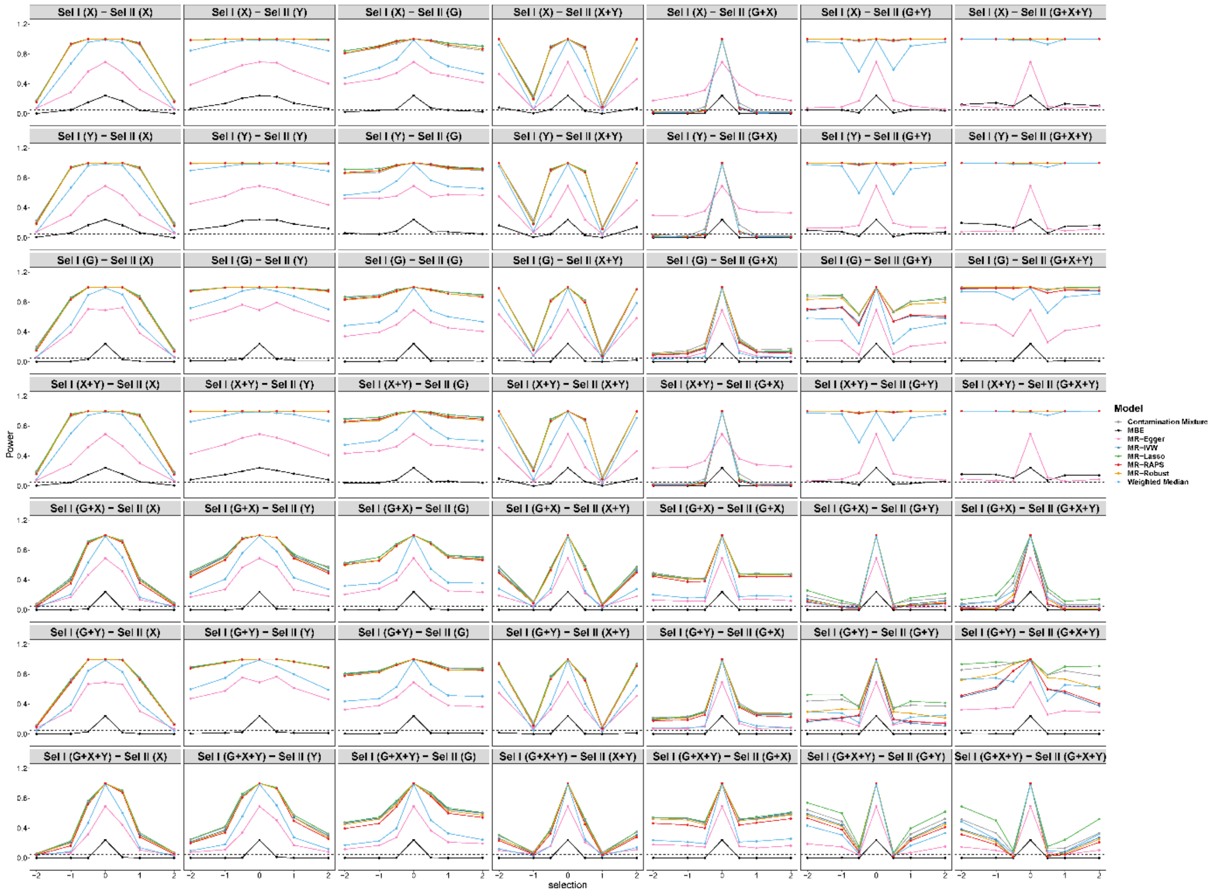

Fig F. Simulation results for statistic power of eight Pleiotropy-robust MR Methods varying across selection effect from -2 to 2 under different selection mechanisms with Positive causal effect in scenario 1 (100 genetic variants).
