# Supplementary material for: Impact of nonrandom selection mechanisms on the causal effect estimation for two-sample Mendelian randomization methods
Source: PLoS Genet. 2022 Mar 17;18(3):e1010107. doi: 10.1371/journal.pgen.1010107 (PMC8963545; doi:10.1371/journal.pgen.1010107)
Supplement: S6 Text — (PDF) [file pgen.1010107.s006.pdf]

# S6 Text

# Simulation results of eight Pleiotropy-robust MR Methods in scenario 2 (Balanced pleiotropy, InSIDE satisfied)

Figs A-F (30% invalid variants, 50 variants) show the tendency of estimations, SEs, type I error rates and statistic power under different selection mechanisms when varying across selection effects of *X*, *Y* or *G* on selection (*S*) in scenario 2 (Balanced pleiotropy, InSIDE satisfied). Each row represents one of seven different selection mechanisms in sample I and columns represent seven different selection mechanisms in sample II.

When the selection mechanism of sample I depend on *X*, the first lines of Fig A illustrate the estimations of exposure on outcome via eight MR methods. We can obtain that the biases of all eight methods are negative and increase with the selection effect increasing when the selection mechanisms of sample II depend on *X*, *X*+*Y*, *G*+*Y*, *G*+*X*+*Y*. The robust biases of all eight models can be obtained when the selection mechanisms of sample II depend on *Y*, *G*, *G*+*X*. When the selection mechanisms of sample I depend on *Y*, *G*, *X*+*Y*, *G*+*Y*, the estimations show similar tendency. However, depending on *G*+*X* and *G*+*X*+*Y* in sample I show different results. When the selection mechanisms of sample II depend on *X*, *Y*, *X*+*Y*, the biases increase, and then decrease with the selection effect increasing. When the selection mechanism of sample II depends on *Y* and *G*, all eight models show biases. In addition, the biases increase and reduce to zero, and then increase with the selection effect increasing when selection mechanism depends on *G*+*Y* or *G*+*X*+*Y*. In summary, nonrandom selection in sample II has a larger influence on biases than those in sample I. With fixed selection mechanism in sample II, the different selection mechanisms I show similarly trend.

Fig B (30% invalid variants, 50 variants) shows the tendency of SEs under different selection mechanisms and simulation situations with a null causal effect ($\theta = 0$) when varying across selection effects of *X*, *Y* or *G* on selection (*S*) in scenario 2. In general, the SEs of MBE model and MR-egger are larger than other models. When the selection mechanism depending on *G*+*Y* or *G*+*X*+*Y* in sample II, the standard errors are larger than other selection mechanisms regardless of the selection mechanism in sample I.

Fig C (30% invalid variants, 50 variants) displays the tendency of Type I error rates under

different selection mechanisms and simulation situations with a null causal effect ( $\theta = 0$ ) when varying across selection effects of $X$, $Y$ or $G$ on selection ($S$) in scenario 2. When the selection mechanisms of sample II depend on G, the type I error rates are closed to 0.05 regardless of the selection mechanism in sample I. Furthermore, the type I error inflation can be observed under different selection mechanisms due to the biased causal effect estimations (Fig C) of exposure on outcome.

Figs D and E (30% invalid variants, 50 variants) display similarly tendency of estimations and SEs with Figs D and E in S5 Text under different selection mechanisms with a positive causal effect ( $\theta = 0.2$ ) when varying across selection effects of $X$, $Y$ or $G$ on selection ($S$). The estimations of MR-egger do not perform as well as other models. Fig F shows the eight methods cannot effectively reject the null hypothesis due to the selection effect in some situations.

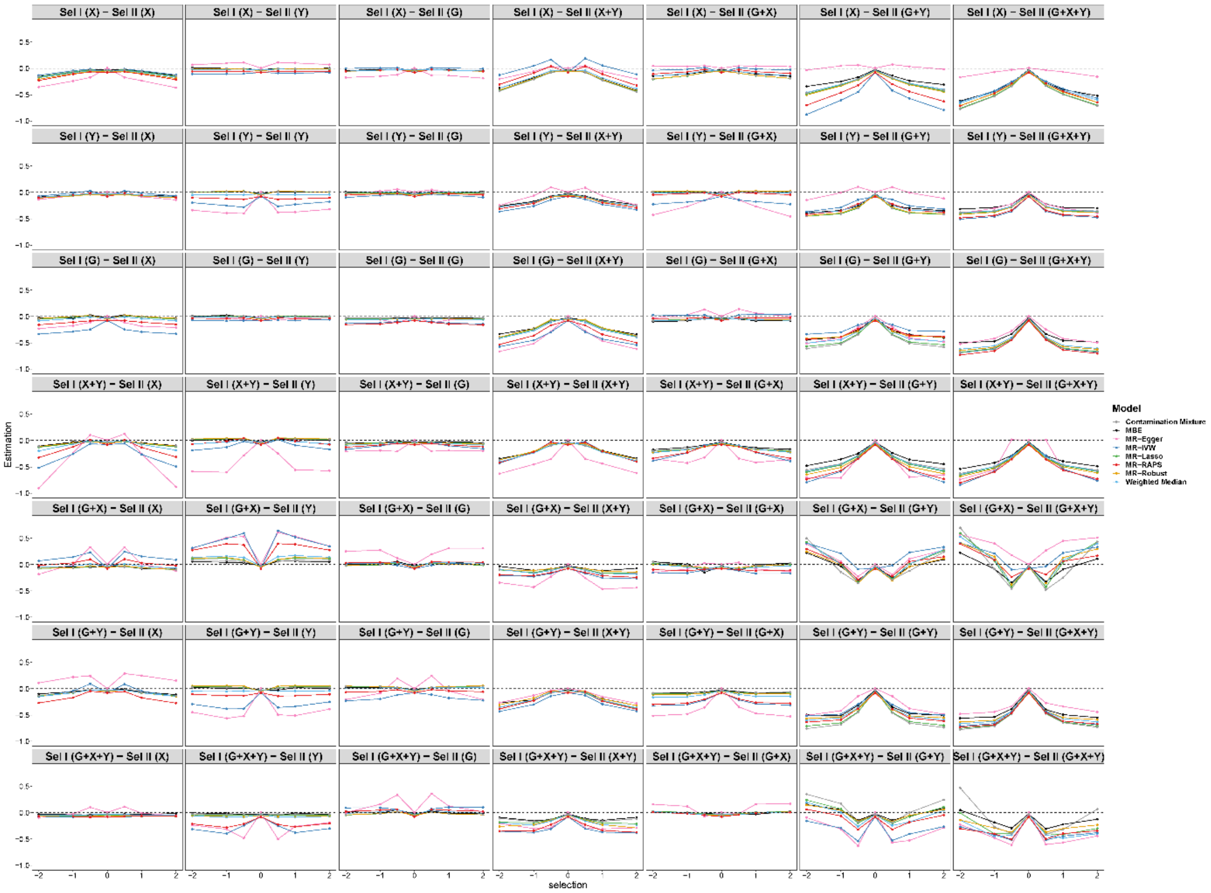

**Fig A.** Simulation results for causal estimations of eight Pleiotropy-robust MR Methods varying across selection effect from -2 to 2 under different selection mechanisms with Null causal effect in scenario 2 (30% invalid variants, 50 genetic variants).

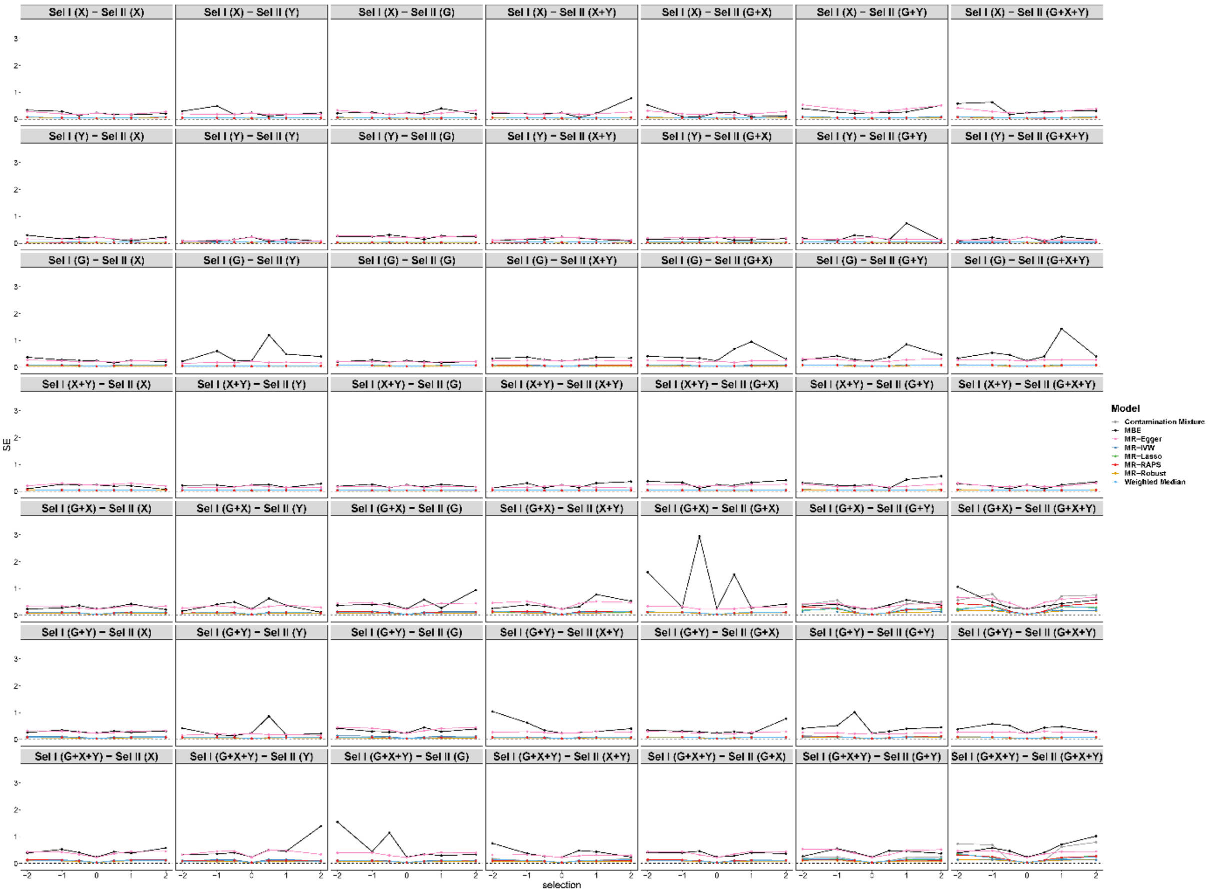


**Fig B.** Simulation results for SEs of eight Pleiotropy-robust MR Methods varying across selection effect from -2 to 2 under different selection mechanisms with Null causal effect in scenario 2 (30% invalid variants, 50 genetic variants).

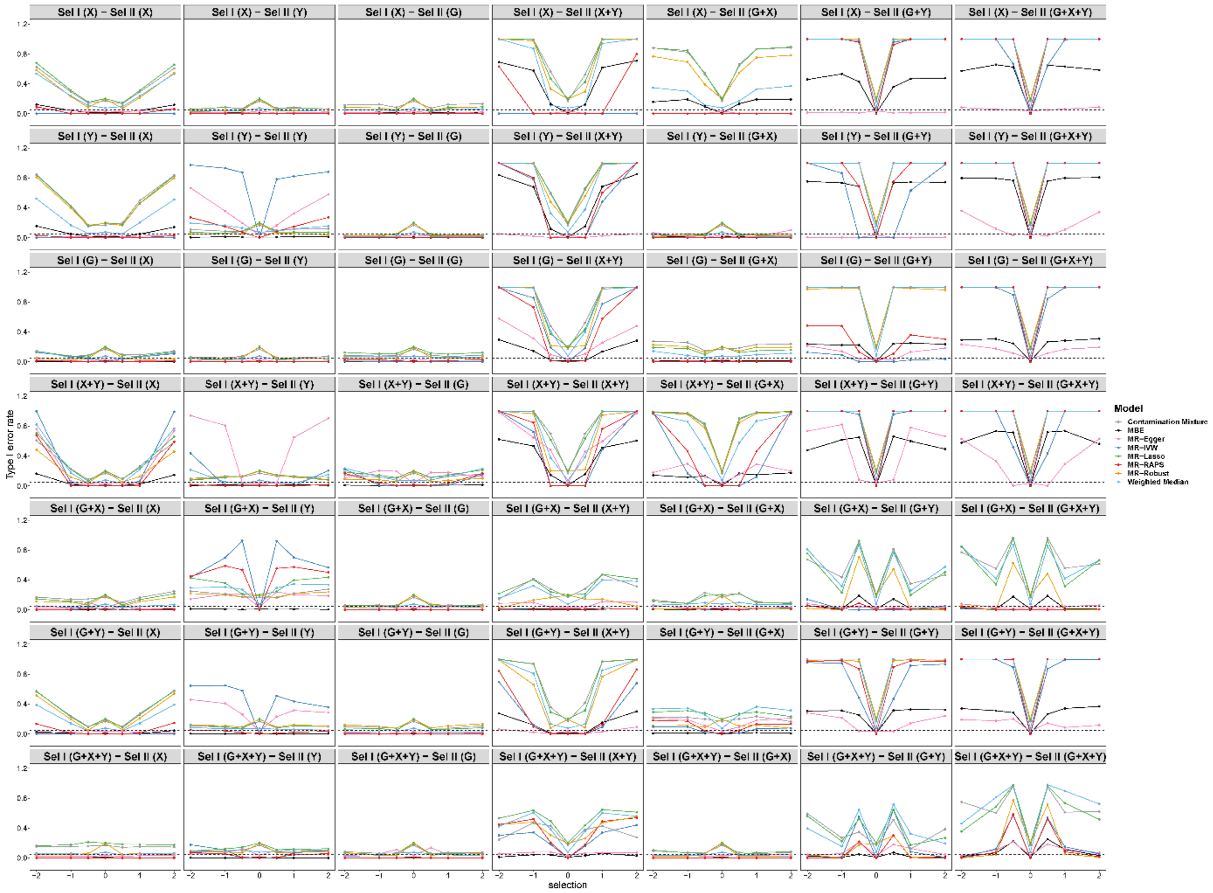


**Fig C.** Simulation results for type I error rates of eight Pleiotropy-robust MR Methods varying across selection effect from -2 to 2 under different selection mechanisms with Null causal effect in scenario 2 (30% invalid variants, 50 genetic variants).

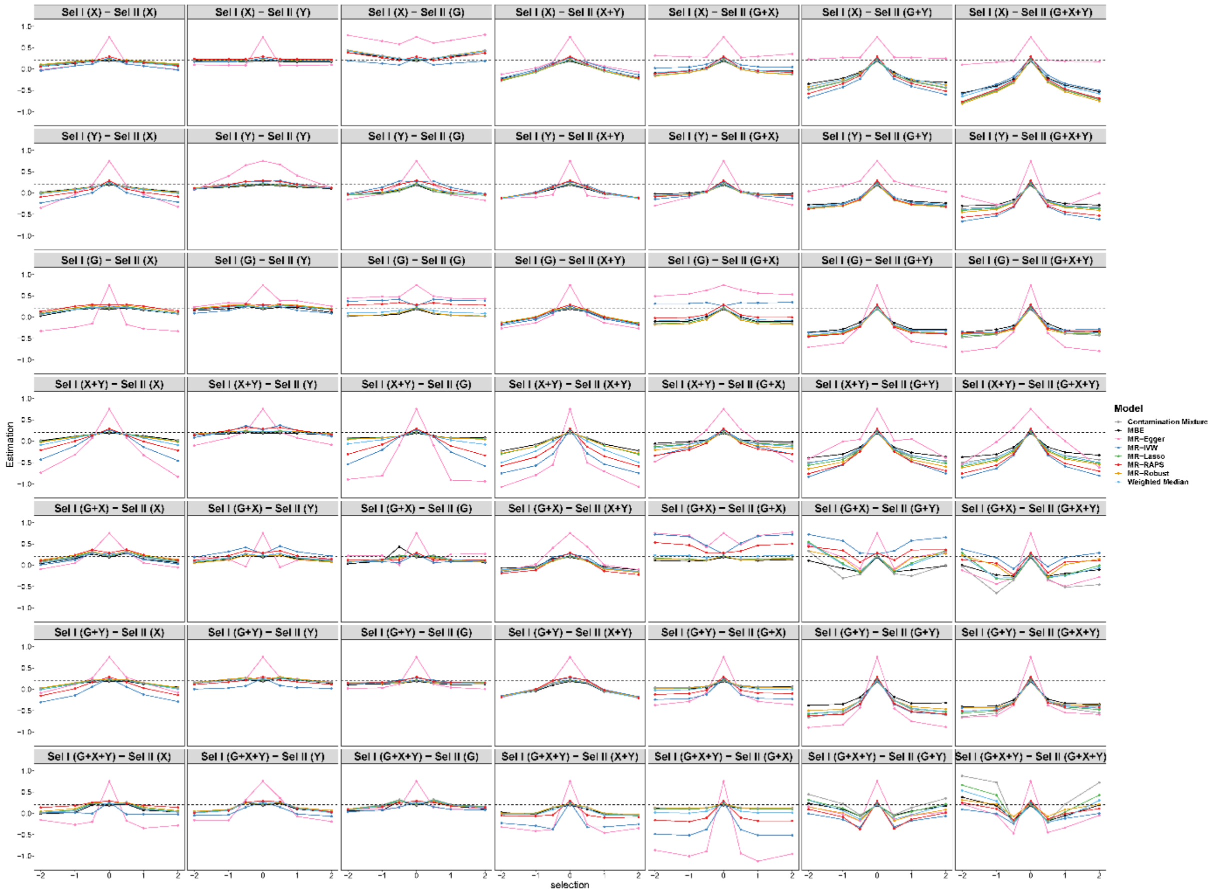


**Fig D.** Simulation results for causal estimations of eight Pleiotropy-robust MR Methods varying across selection effect from -2 to 2 under different selection mechanisms with Positive causal effect in scenario 2 (30% invalid variants, 50 genetic variants).

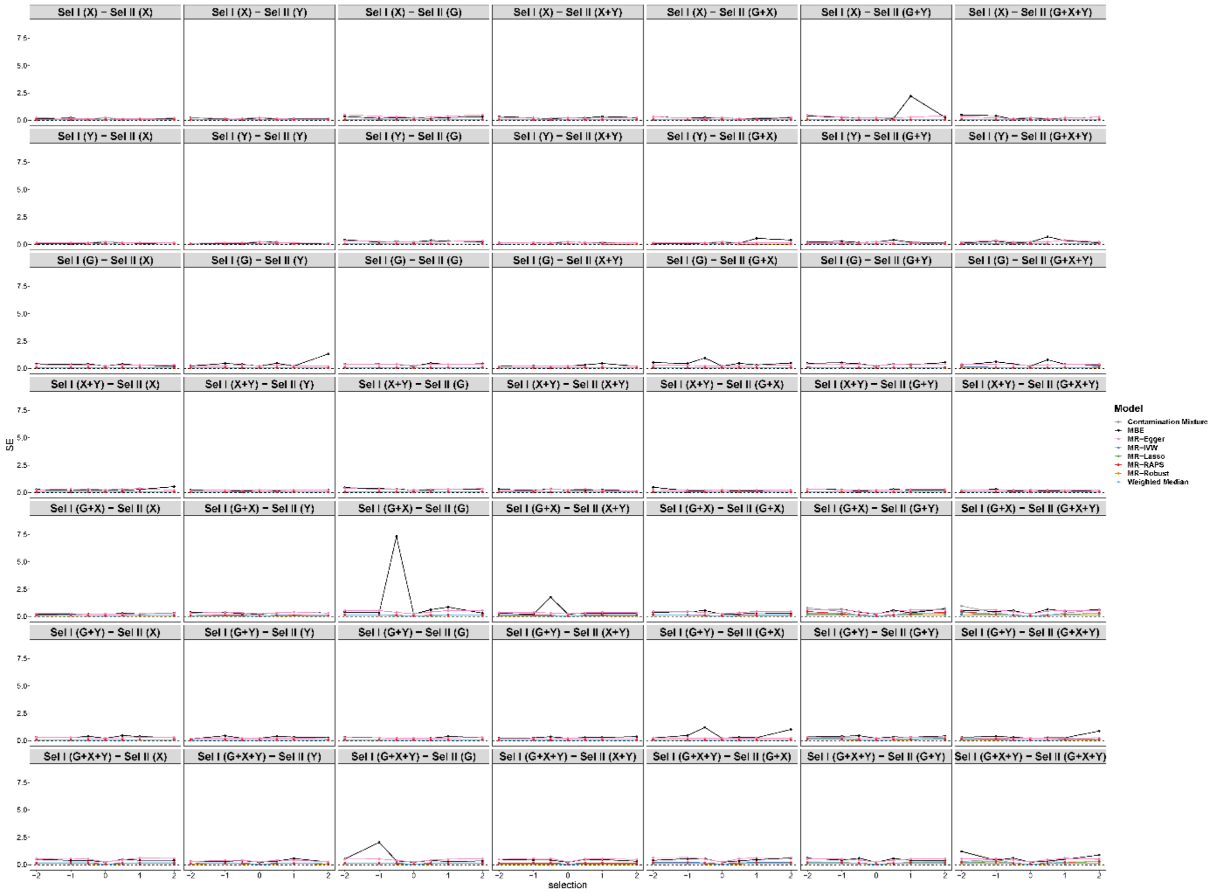


**Fig E.** Simulation results for SEs of eight Pleiotropy-robust MR Methods varying across selection effect from -2 to 2 under different selection mechanisms with Positive causal effect in scenario 1 (30% invalid variants, 50 genetic variants).

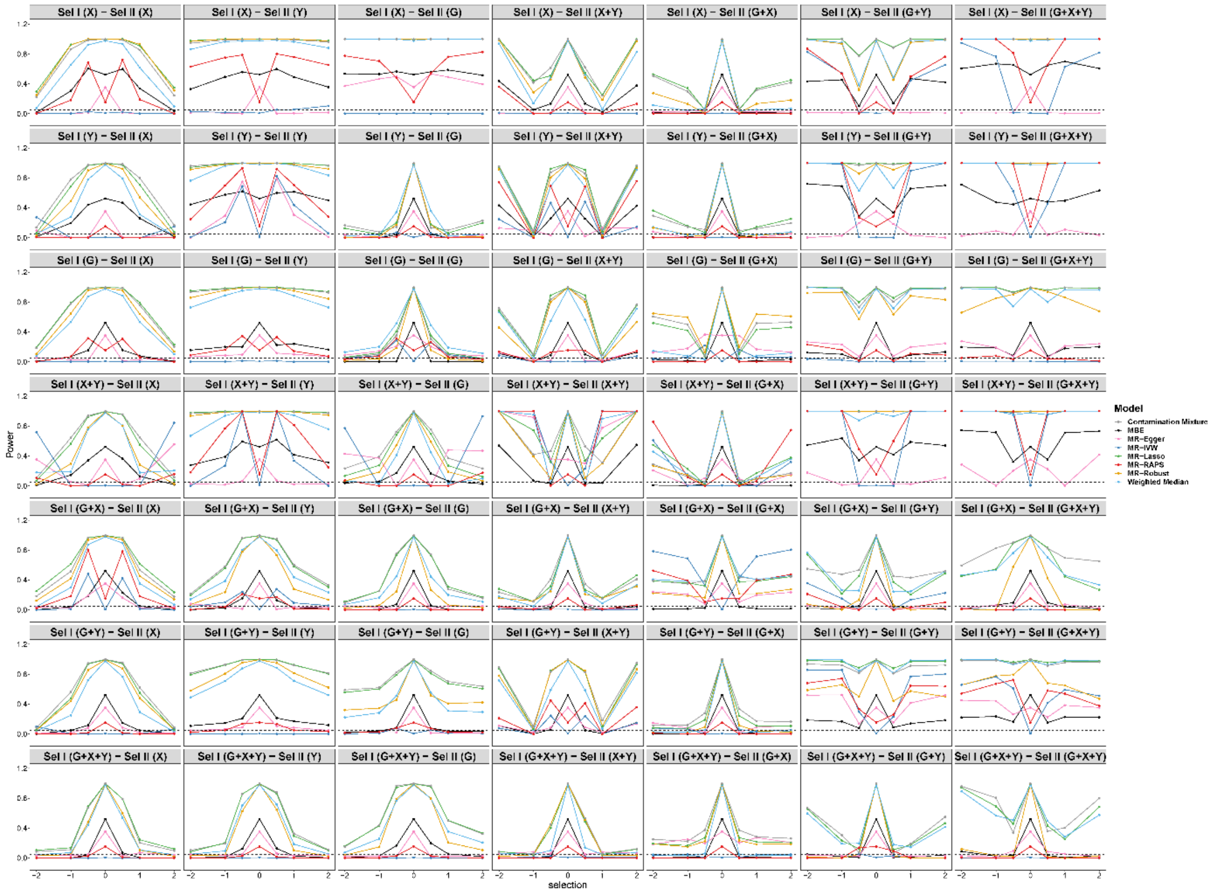


**Fig F.** Simulation results for statistic power of eight Pleiotropy-robust MR Methods varying across selection effect from -2 to 2 under different selection mechanisms with Positive causal effect in scenario 1 (30% invalid variants, 50 genetic variants).

Figs G-L (30% invalid variants, 100 variants), Figs M-R (70% invalid variants, 50 variants) and Figs S-X (70% invalid variants, 100 variants) show similarly tendency of estimations, SEs, type I error rates and statistic power under different selection mechanisms and simulation situations when varying across selection effects of $X$, $Y$ or $G$ on selection ($S$) in scenario 2 (Balanced pleiotropy, InSIDE satisfied). In summary, the biases of all models increases with proportion of invalid variants increasing.

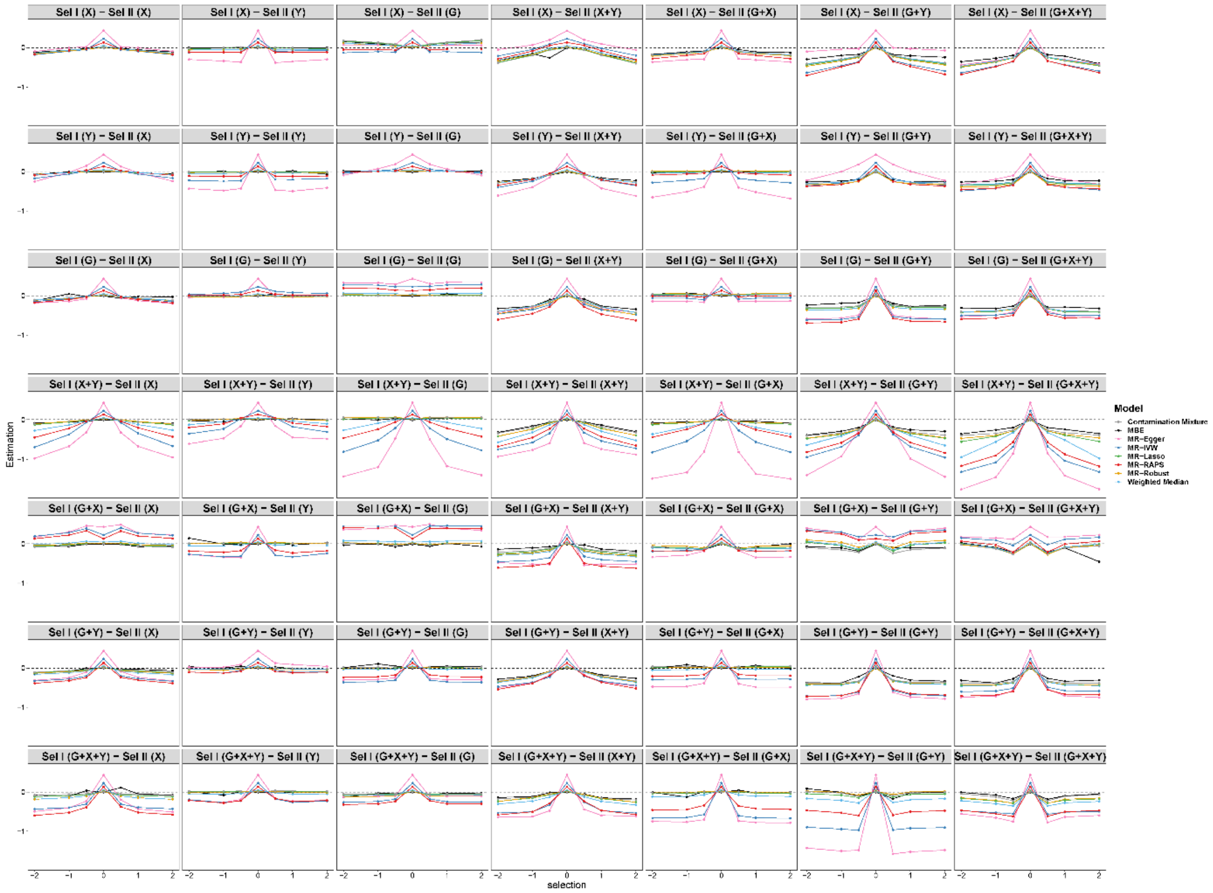

**Fig G.** Simulation results for causal estimations of eight Pleiotropy-robust MR Methods varying across selection effect from -2 to 2 under different selection mechanisms with Null causal effect in scenario 2 (30% invalid variants, 100 genetic variants).

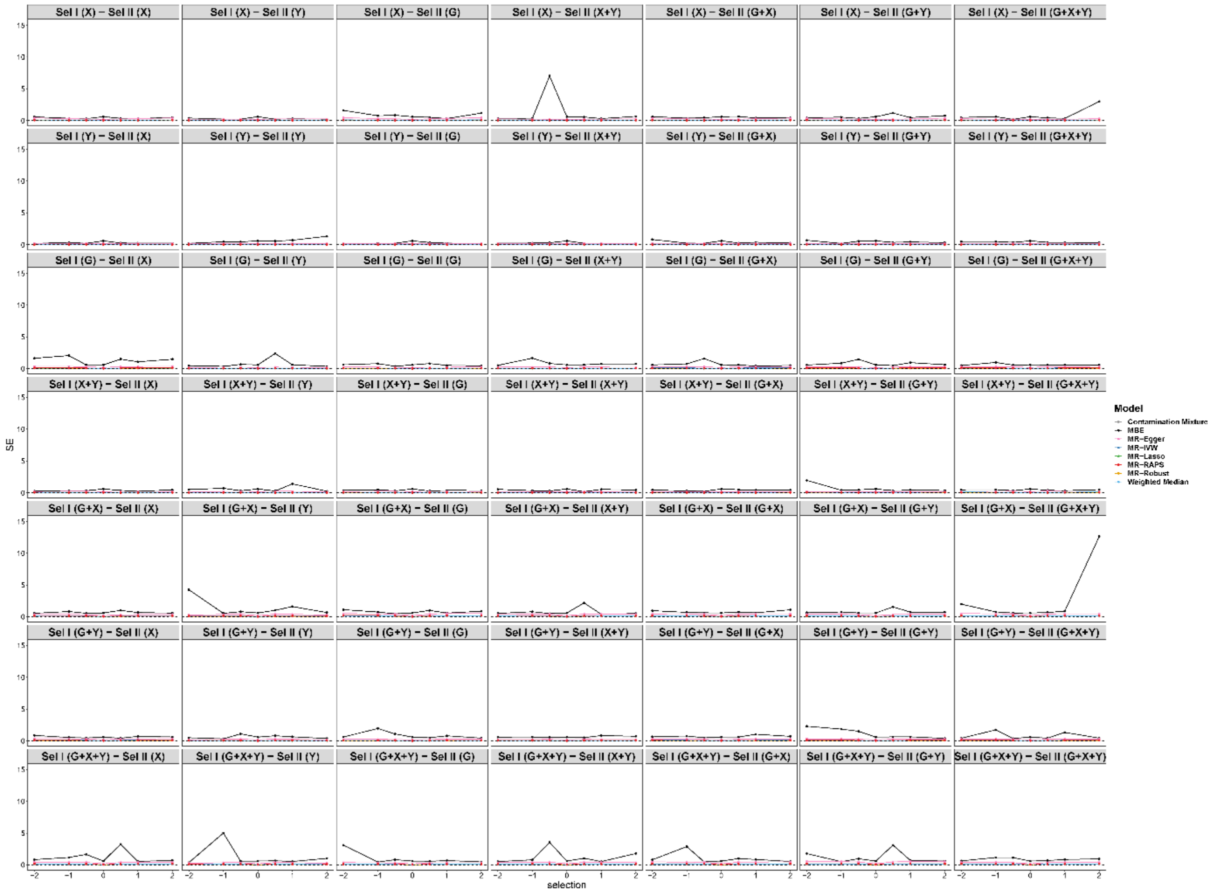


**Fig H.** Simulation results for SEs of eight Pleiotropy-robust MR Methods varying across selection effect from -2 to 2 under different selection mechanisms with Null causal effect in scenario 2 (30% invalid variants, 100 genetic variants).

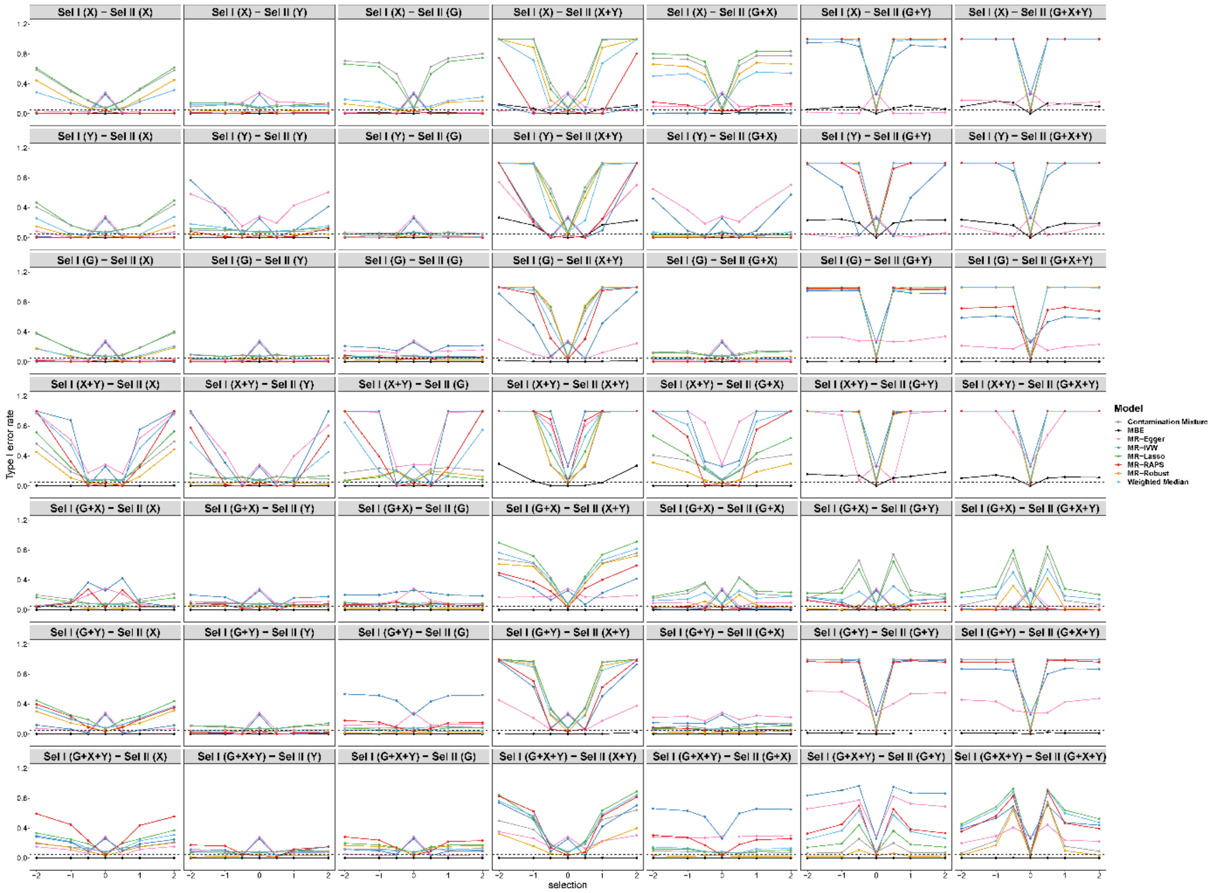


**Fig I.** Simulation results for type I error rates of eight Pleiotropy-robust MR Methods varying across selection effect from -2 to 2 under different selection mechanisms with Null causal effect in scenario 2 (30% invalid variants, 100 genetic variants).

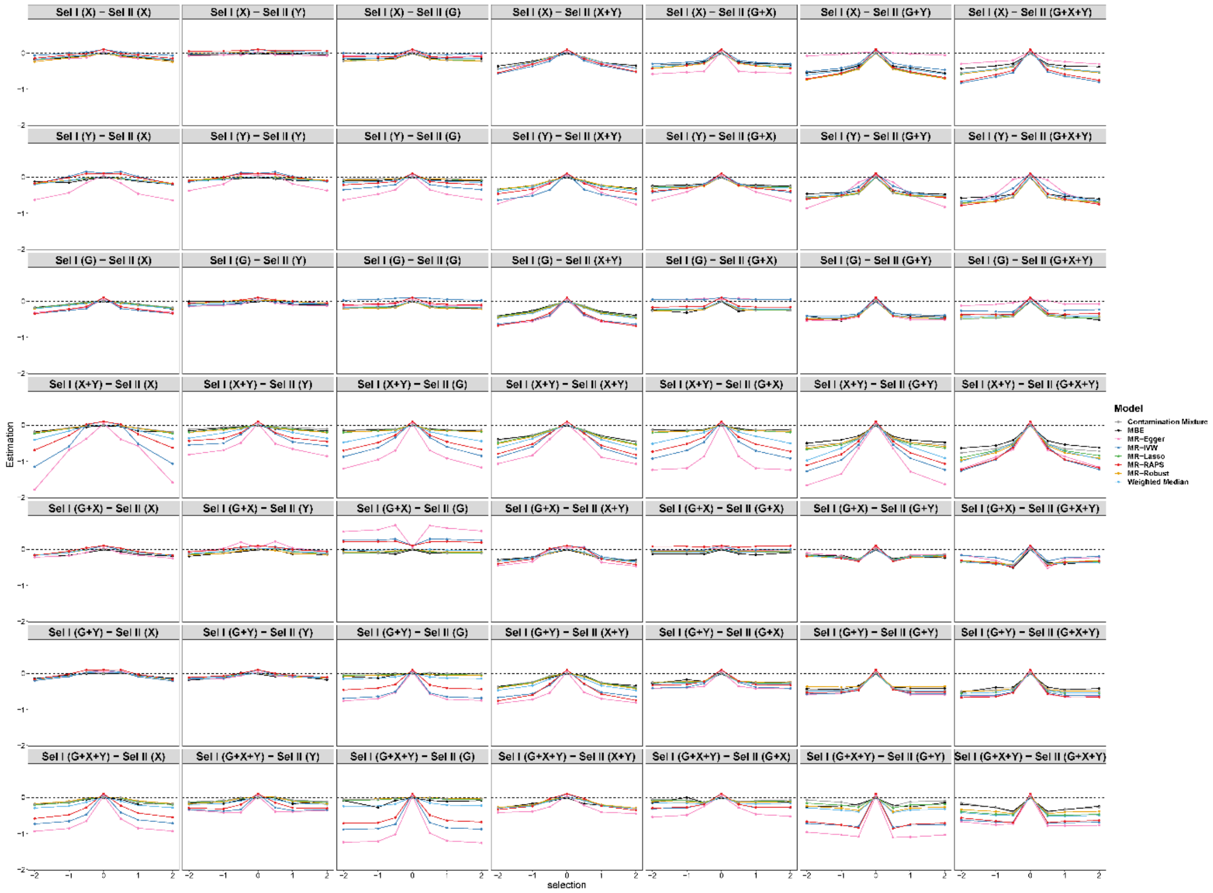


**Fig J.** Simulation results for causal estimations of eight Pleiotropy-robust MR Methods varying across selection effect from -2 to 2 under different selection mechanisms with Positive causal effect in scenario 2 (30% invalid variants, 100 genetic variants).

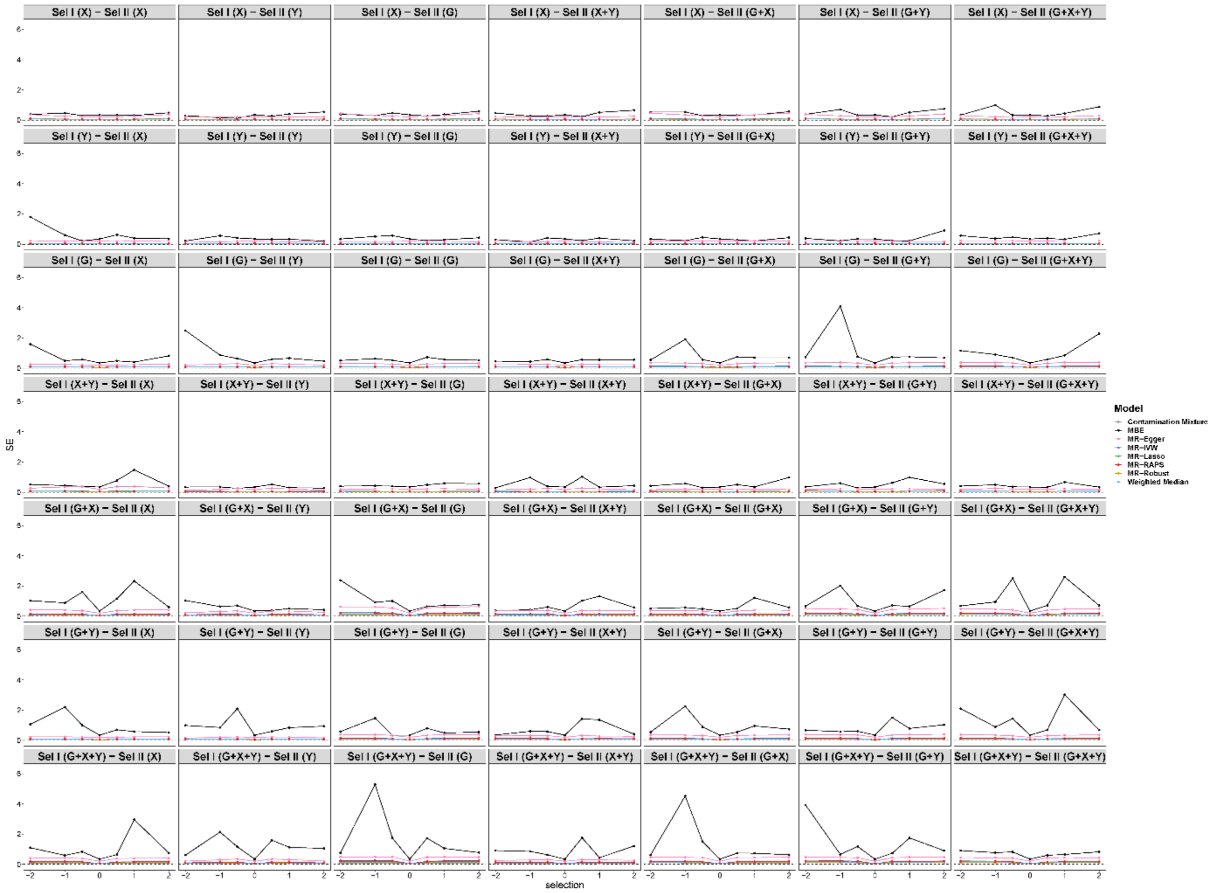

**Fig K.** Simulation results for SEs of eight Pleiotropy-robust MR Methods varying across selection effect from -2 to 2 under different selection mechanisms with Positive causal effect in scenario 2 (30% invalid variants, 100 genetic variants).

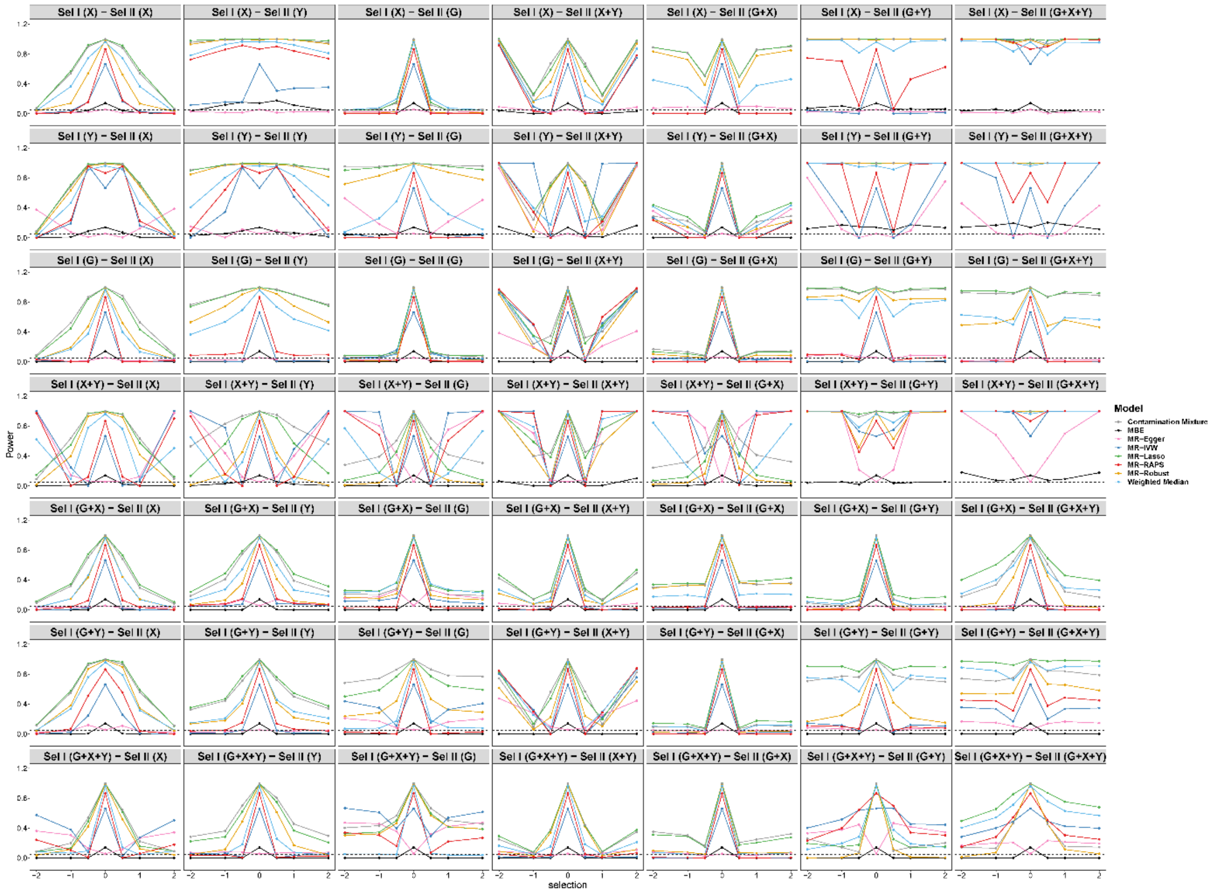


**Fig L**. Simulation results for type I error rates of eight Pleiotropy-robust MR Methods varying across selection effect from -2 to 2 under different selection mechanisms with Positive causal effect in scenario 2 (30% invalid variants, 100 genetic variants).

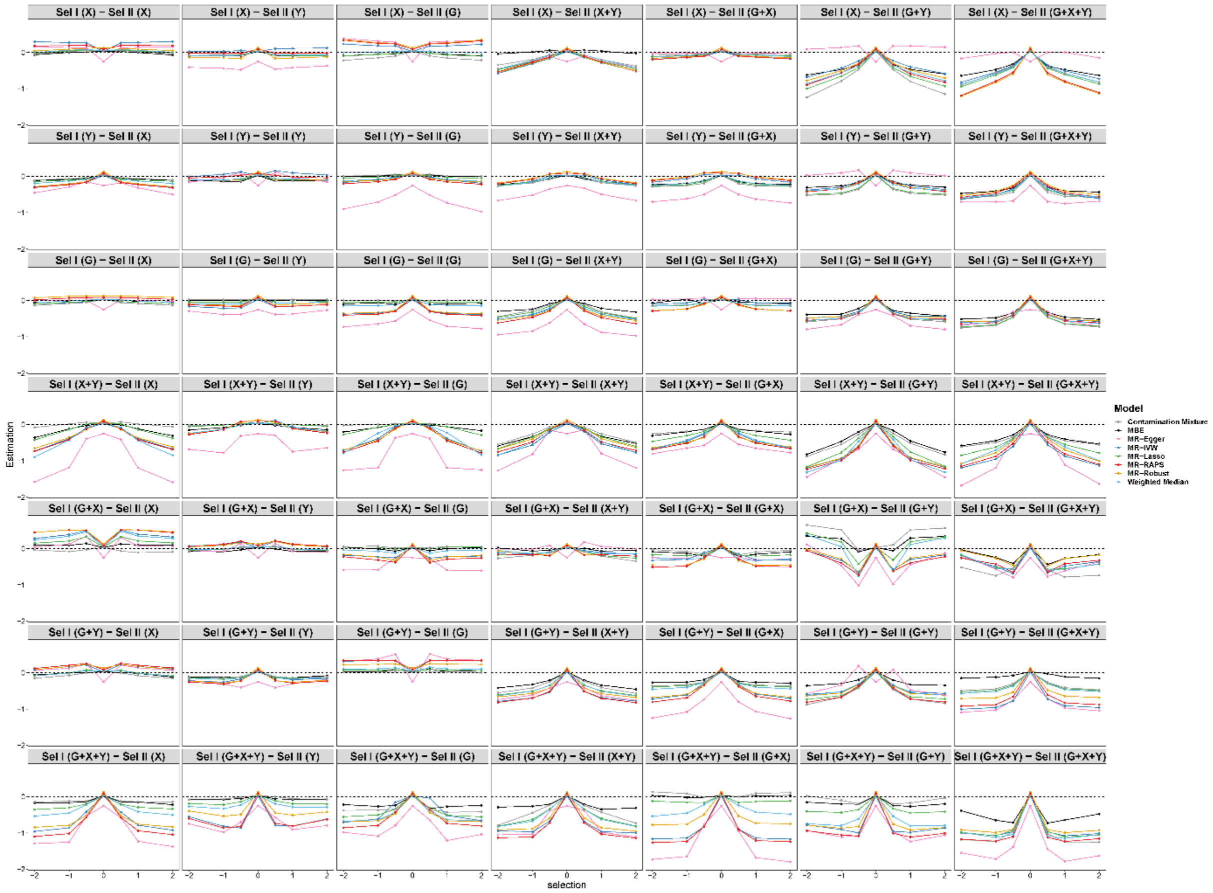


**Fig M.** Simulation results for causal estimations of eight Pleiotropy-robust MR Methods varying across selection effect from -2 to 2 under different selection mechanisms with Null causal effect in scenario 2 (70% invalid variants, 50 genetic variants).

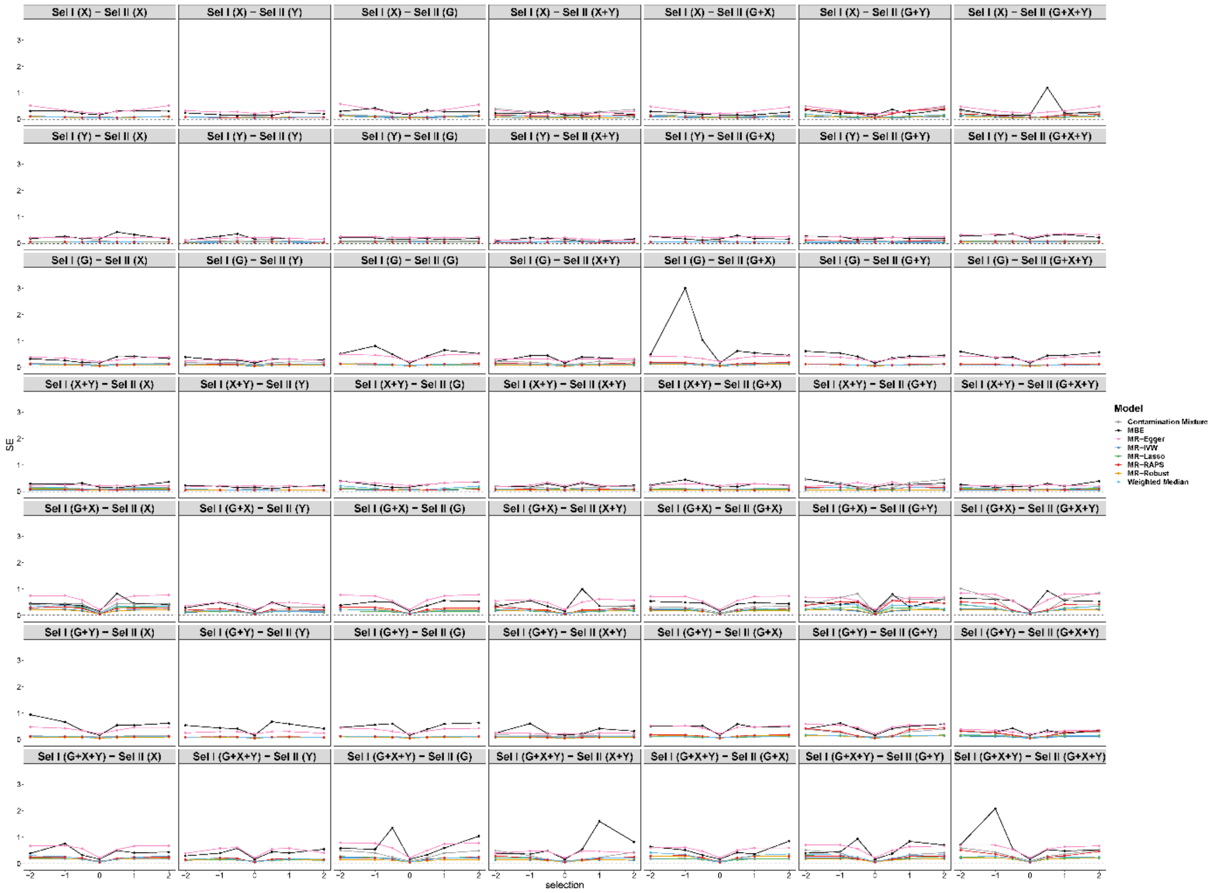

**Fig N.** Simulation results for SEs of eight Pleiotropy-robust MR Methods varying across selection effect from -2 to 2 under different selection mechanisms with Null causal effect in scenario 2 (70% invalid variants, 50 genetic variants).

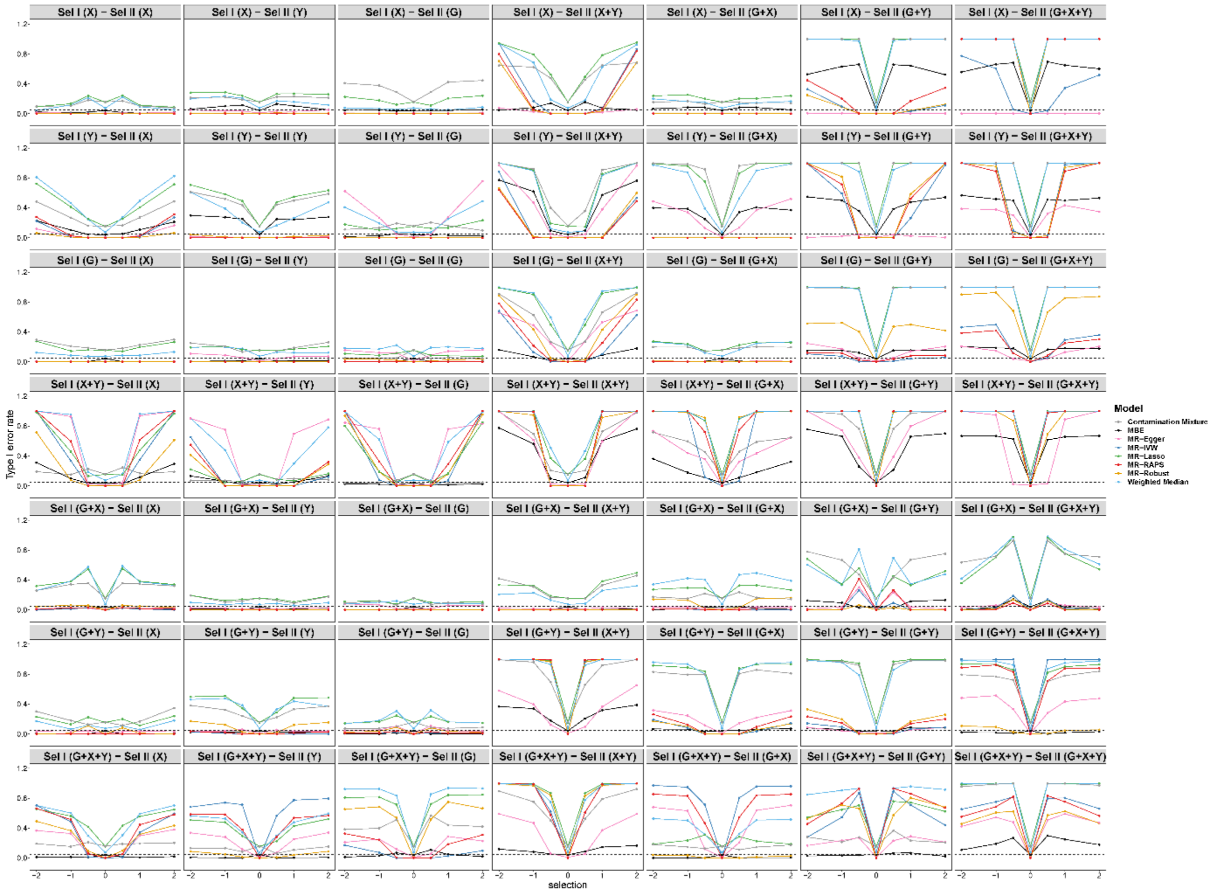


**Fig O.** Simulation results for type I error rates of eight Pleiotropy-robust MR Methods varying across selection effect from -2 to 2 under different selection mechanisms with Null causal effect in scenario 2 (70% invalid variants, 50 genetic variants).

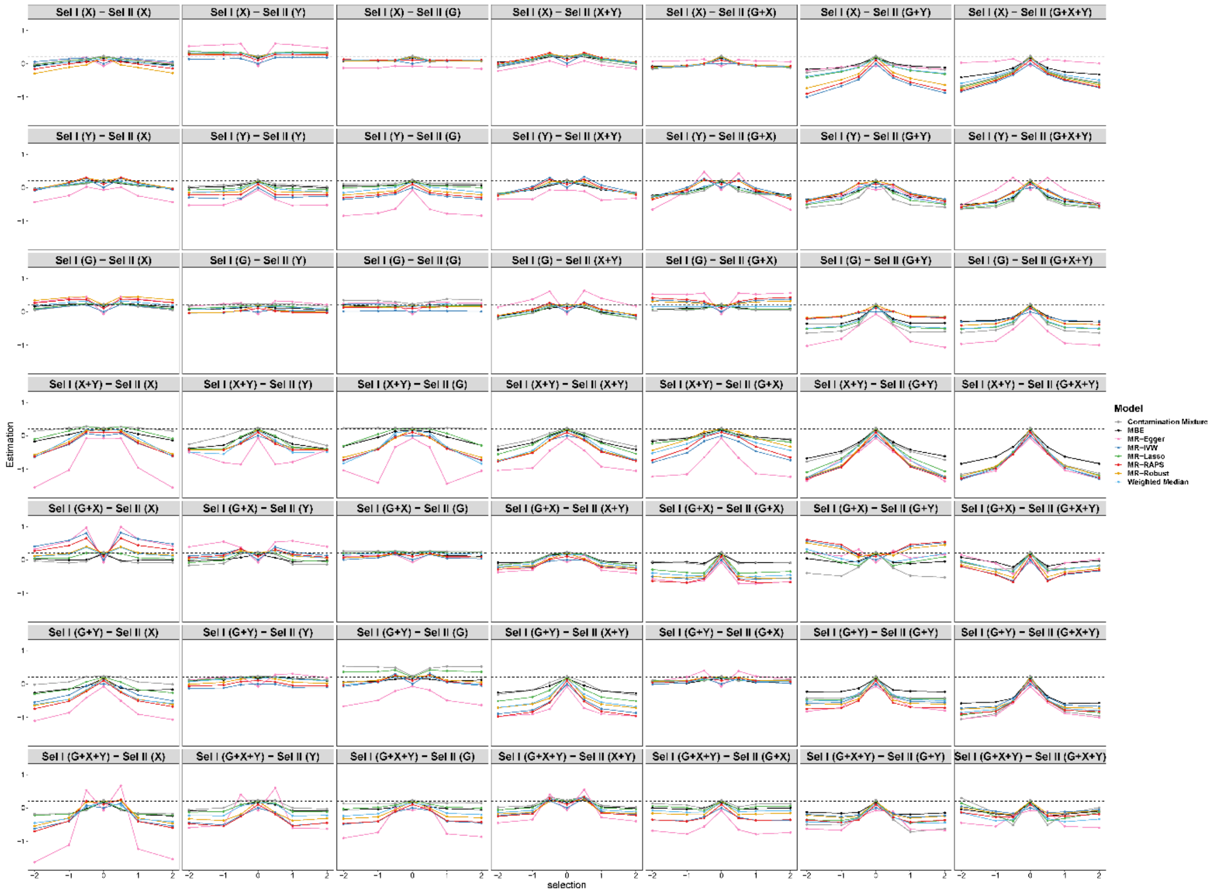

**Fig P.** Simulation results for causal estimations of eight Pleiotropy-robust MR Methods varying across selection effect from -2 to 2 under different selection mechanisms with Positive causal effect in scenario 2 (70% invalid variants, 50 genetic variants).

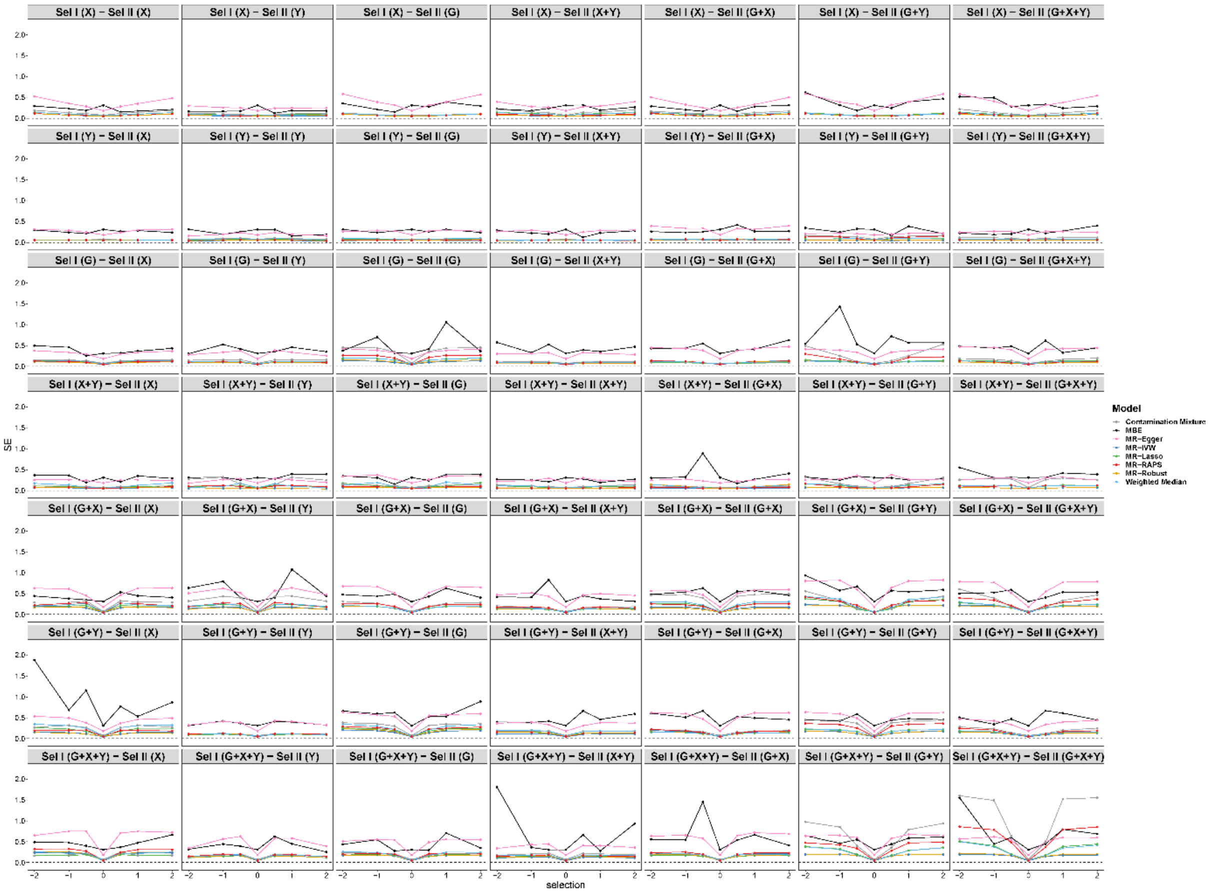


**Fig Q.** Simulation results for SEs of eight Pleiotropy-robust MR Methods varying across selection effect from -2 to 2 under different selection mechanisms with Positive causal effect in scenario 2 (70% invalid variants, 50 genetic variants).

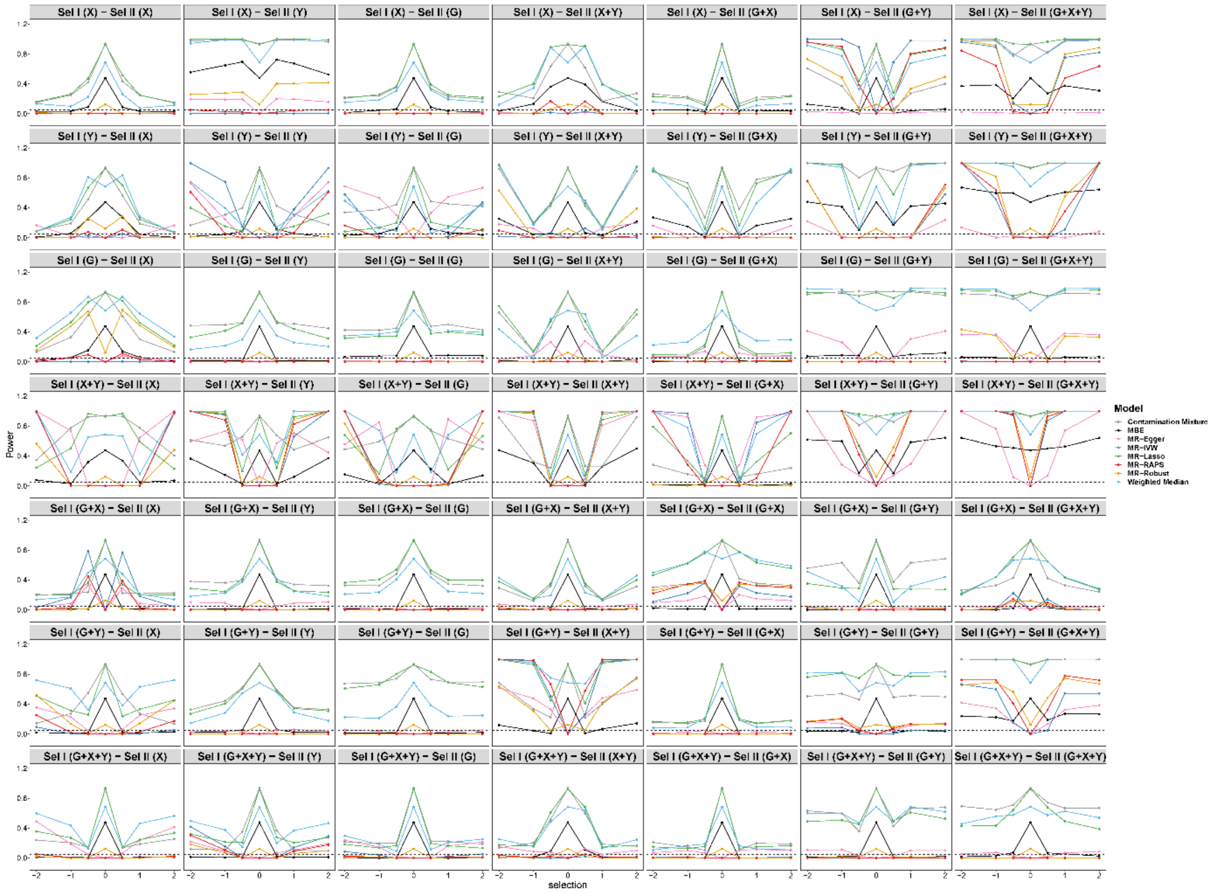


**Fig R.** Simulation results for statistic power of eight Pleiotropy-robust MR Methods varying across selection effect from -2 to 2 under different selection mechanisms with Positive causal effect in scenario 2 (70% invalid variants, 50 genetic variants).

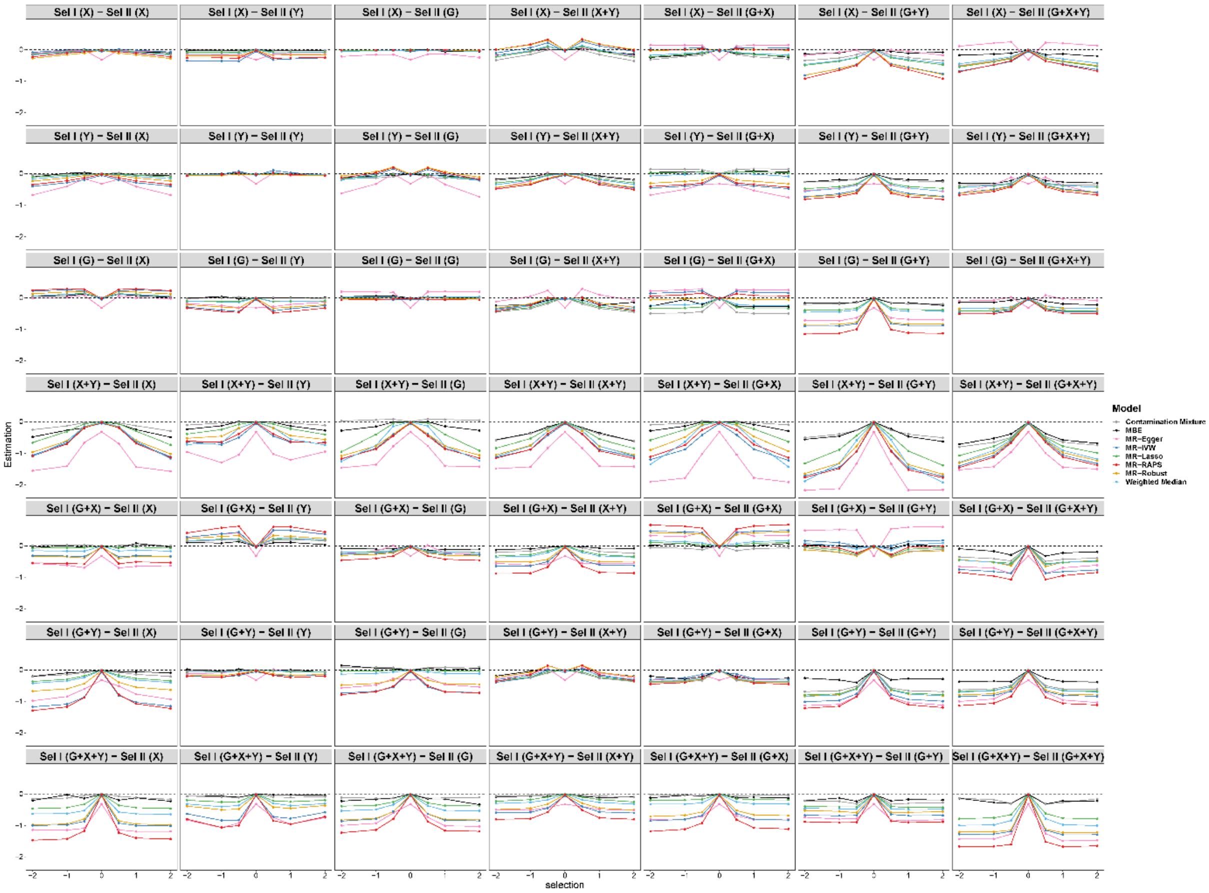


**Fig S.** Simulation results for causal estimations of eight Pleiotropy-robust MR Methods varying across selection effect from -2 to 2 under different selection mechanisms with Null causal effect in scenario 2 (70% invalid variants, 100 genetic variants).

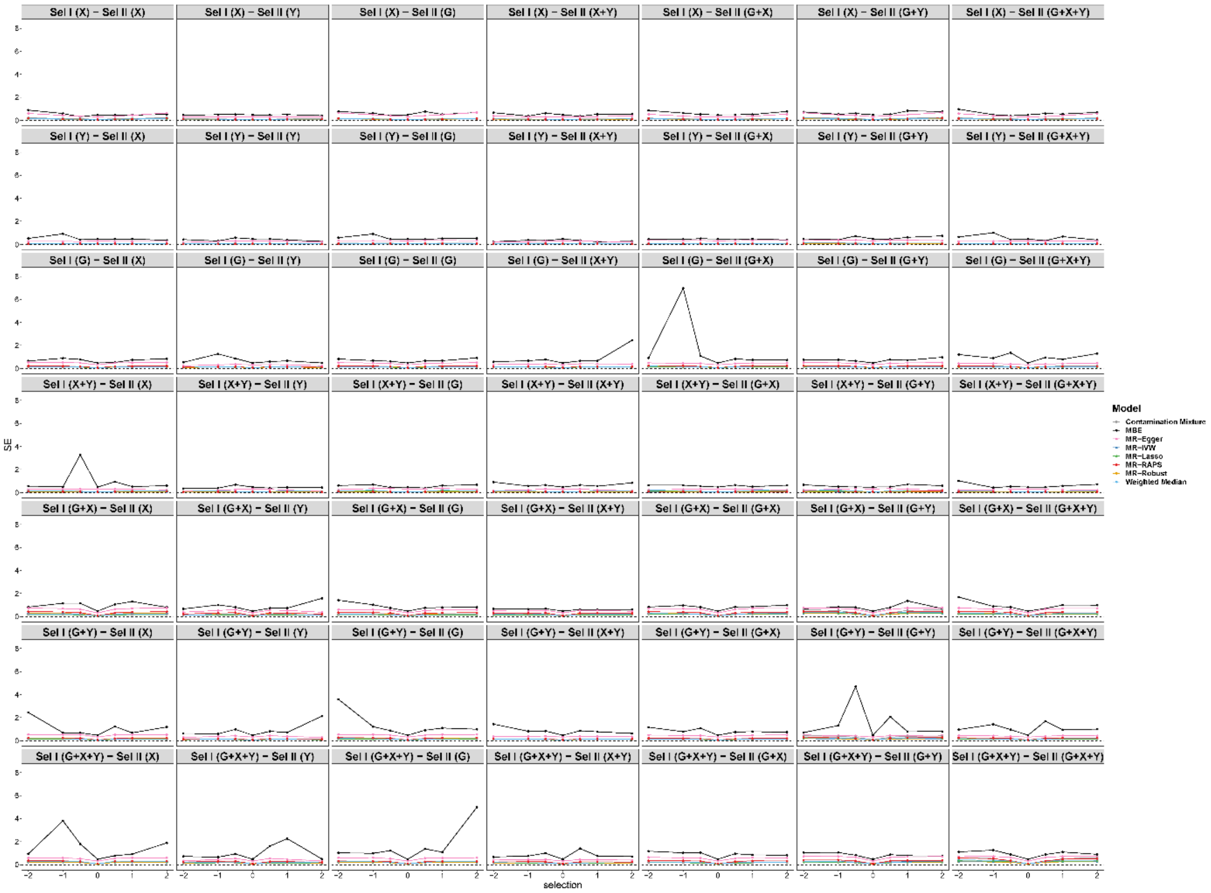


**Fig T.** Simulation results for SEs of eight Pleiotropy-robust MR Methods varying across selection effect from -2 to 2 under different selection mechanisms with Null causal effect in scenario 2 (70% invalid variants, 100 genetic variants).

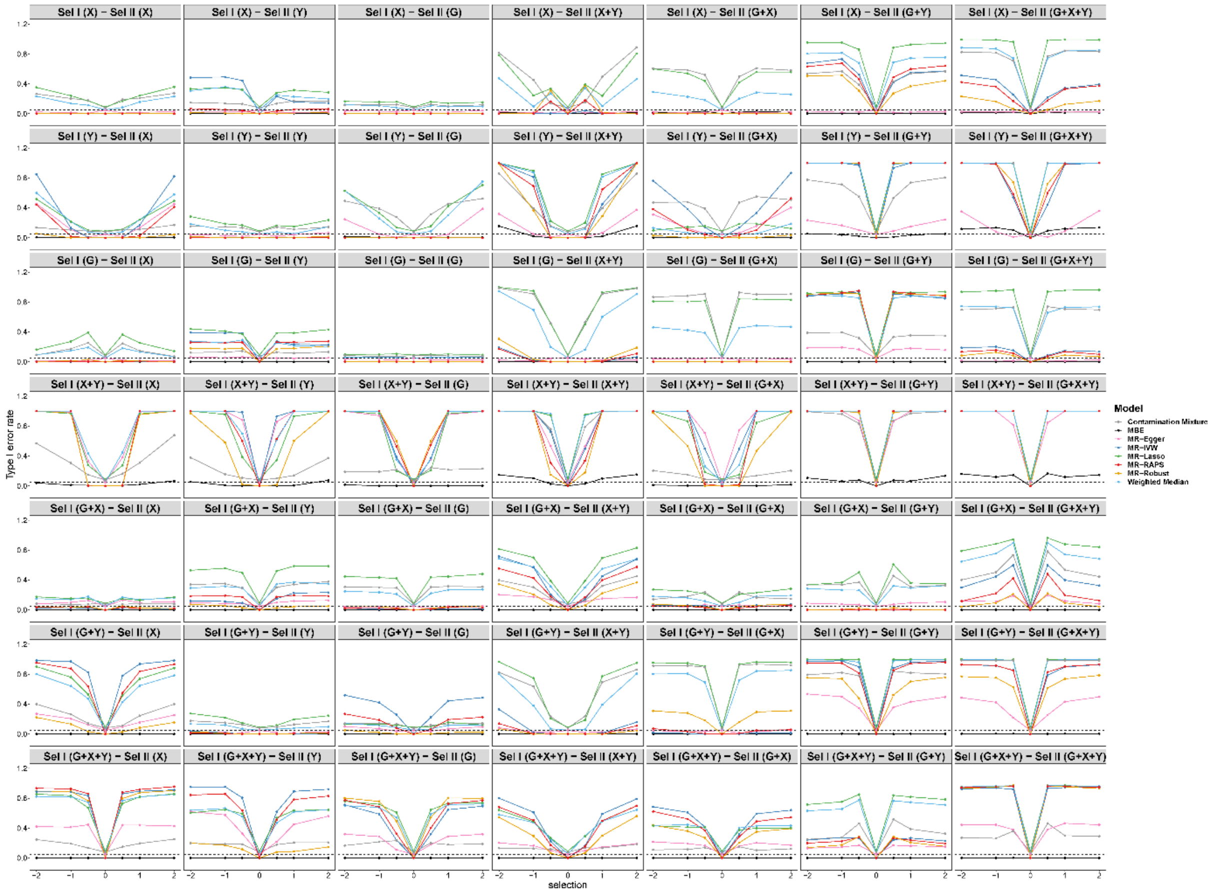

**Fig U.** Simulation results for type I error rates of eight Pleiotropy-robust MR Methods varying across selection effect from -2 to 2 under different selection mechanisms with Null causal effect in scenario 2 (70% invalid variants, 100 genetic variants).

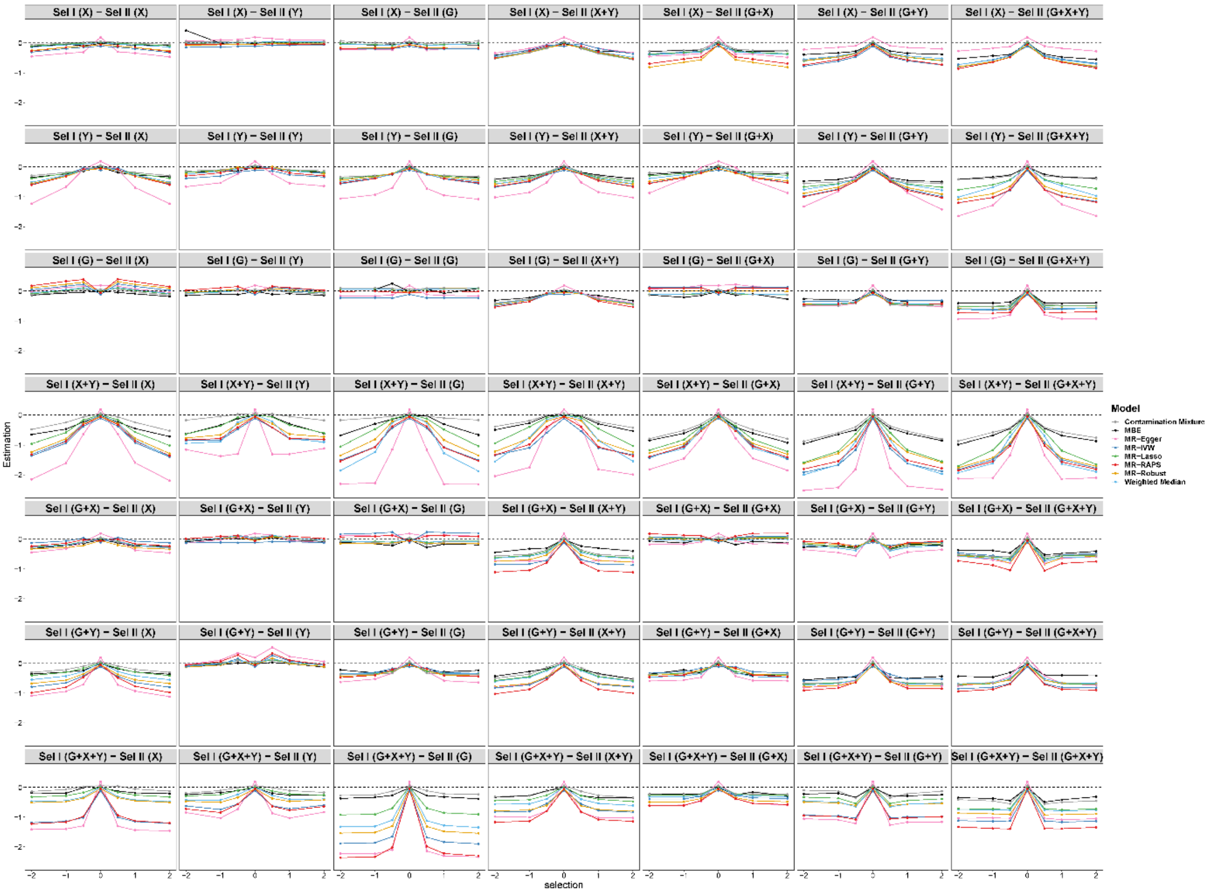


**Fig V.** Simulation results for causal estimation of eight Pleiotropy-robust MR Methods varying across selection effect from -2 to 2 under different selection mechanisms with Positive causal effect in scenario 2 (70% invalid variants, 100 genetic variants).

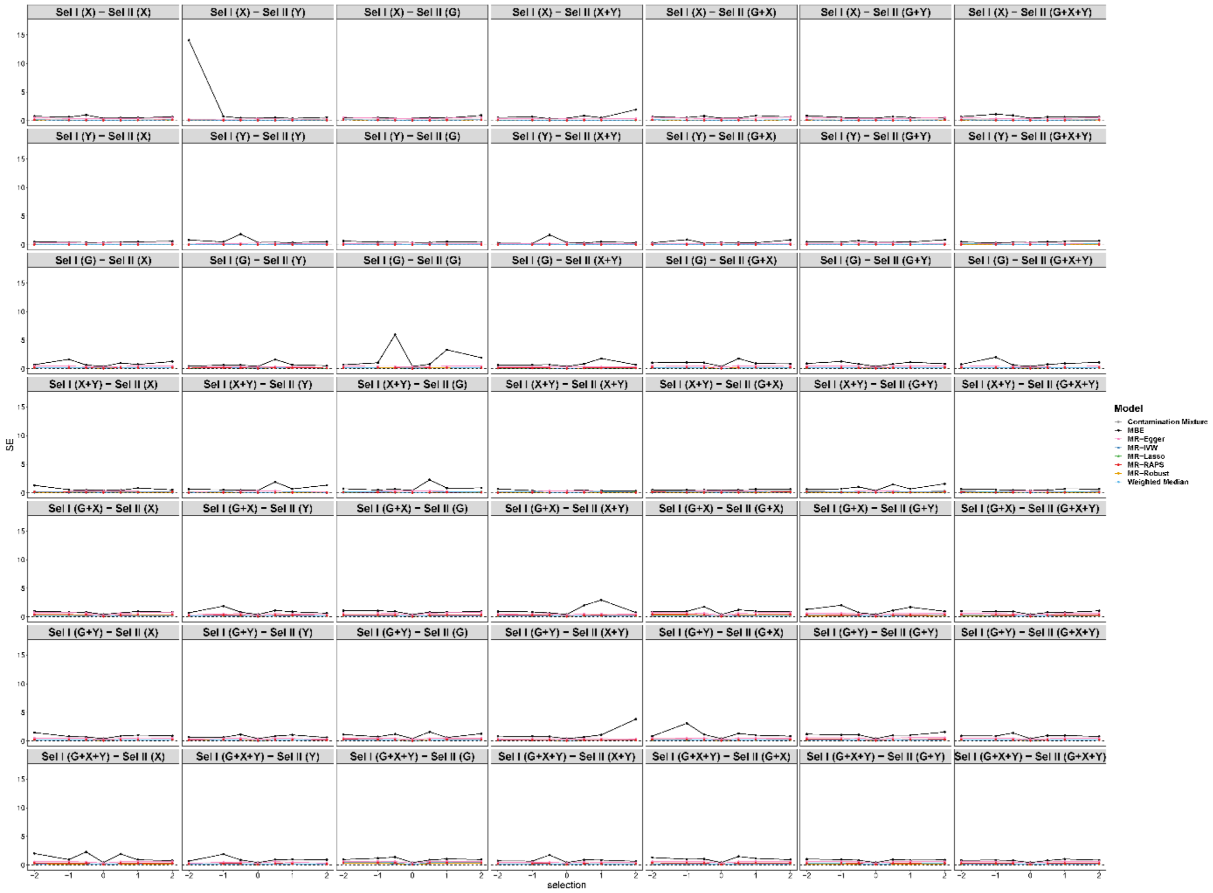


**Fig W.** Simulation results for SEs of eight Pleiotropy-robust MR Methods varying across selection effect from -2 to 2 under different selection mechanisms with Positive causal effect in scenario 2 (70% invalid variants, 100 genetic variants).

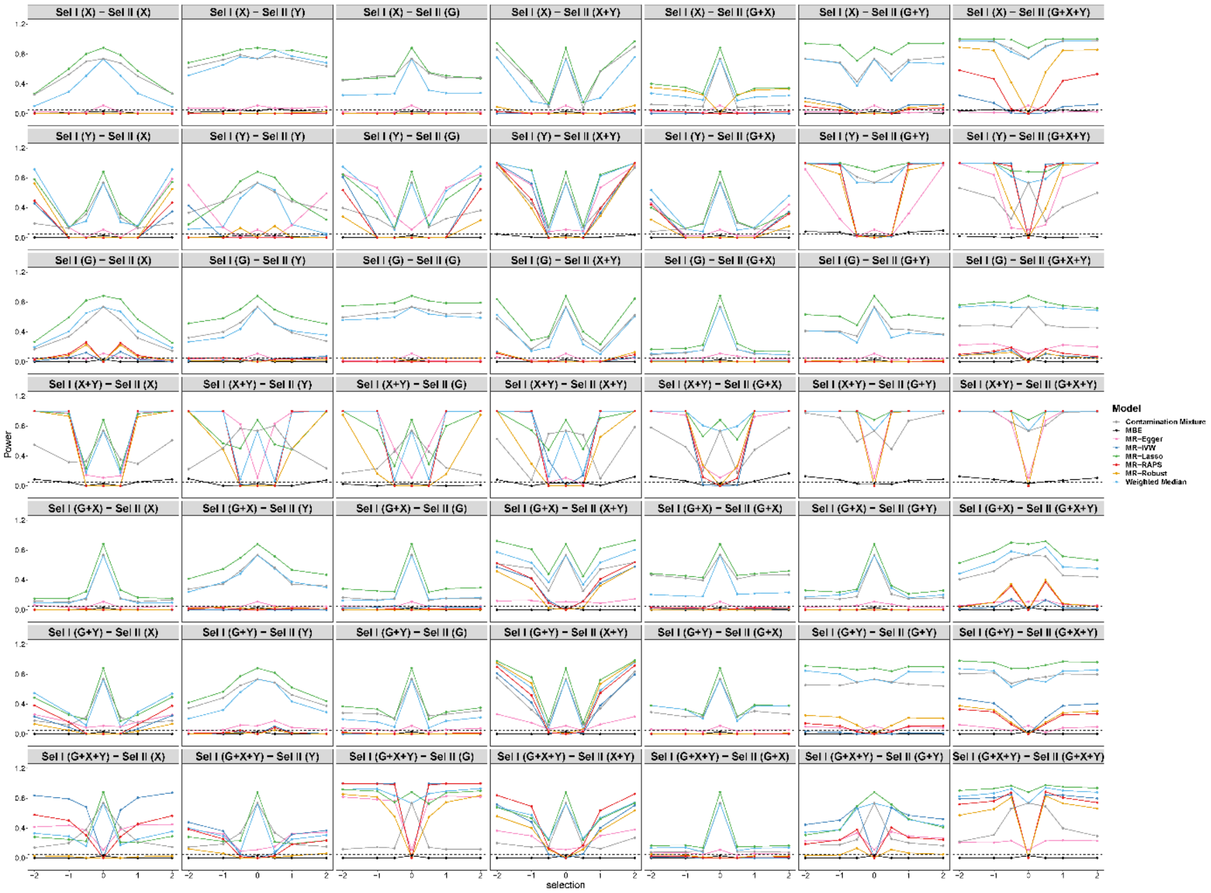


**Fig X.** Simulation results for power of eight Pleiotropy-robust MR Methods varying across selection effect from -2 to 2 under different selection mechanisms with Positive causal effect in scenario 2 (70% invalid variants, 100 genetic variants).
