# Supplementary material for: Impact of nonrandom selection mechanisms on the causal effect estimation for two-sample Mendelian randomization methods
Source: PLoS Genet. 2022 Mar 17;18(3):e1010107. doi: 10.1371/journal.pgen.1010107 (PMC8963545; doi:10.1371/journal.pgen.1010107)
Supplement: S7 Text — (PDF) [file pgen.1010107.s007.pdf]

S7 Text

Simulation results of eight Pleiotropy-robust MR Methods in scenario 3 (Directional pleiotropy, InSIDE satisfied)

Figs A-F (30% invalid variants, 50 variants) show the tendency of estimations, SEs, type I error rates and statistic power under different selection mechanisms and simulation situations when varying across selection effects of *X*, *Y* or *G* on selection (*S*) in scenario 3 (Directional pleiotropy, InSIDE satisfied). Due to directional pleiotropy and selection mechanisms, almost all models show biased causal effect estimations (Fig A). The biases for selection mechanism depending on *G*+*Y* and *G*+*X*+*Y* in sample II are larger than other selection mechanisms regardless of the selection mechanism in sample I. Fig B (30% invalid variants, 50 variants) shows the tendency of SEs under different selection mechanisms and simulation situations with a null causal effect ($\theta = 0$) when varying across selection effects of *X*, *Y* or *G* on selection (*S*). In general, the SEs of MBE model and MR-egger are larger than other models. The SEs for selection mechanism depending on *G*+*Y* and *G*+*X*+*Y* in sample II are larger than other selection mechanisms regardless of the selection mechanism in sample I. Fig C (30% invalid variants, 50 variants) displays the tendency of Type I error rates under different selection mechanisms and simulation situations with a null causal effect ($\theta = 0$) when varying across selection effects of *X*, *Y* or *G* on selection (*S*) in scenario 3. The type I error inflation can be observed under different selection mechanisms due to the biased causal effect estimations (Fig A) of exposure on outcome. Figs D and E (30% invalid variants, 50 variants) display similarly tendency of estimations and SEs with Figs A and B under different selection mechanisms with a positive causal effect ($\theta = 0.2$) when varying across selection effects of *X*, *Y* or *G* on selection (*S*). Fig F shows all eight methods cannot effectively reject the null hypothesis due to the selection effect in some situations.

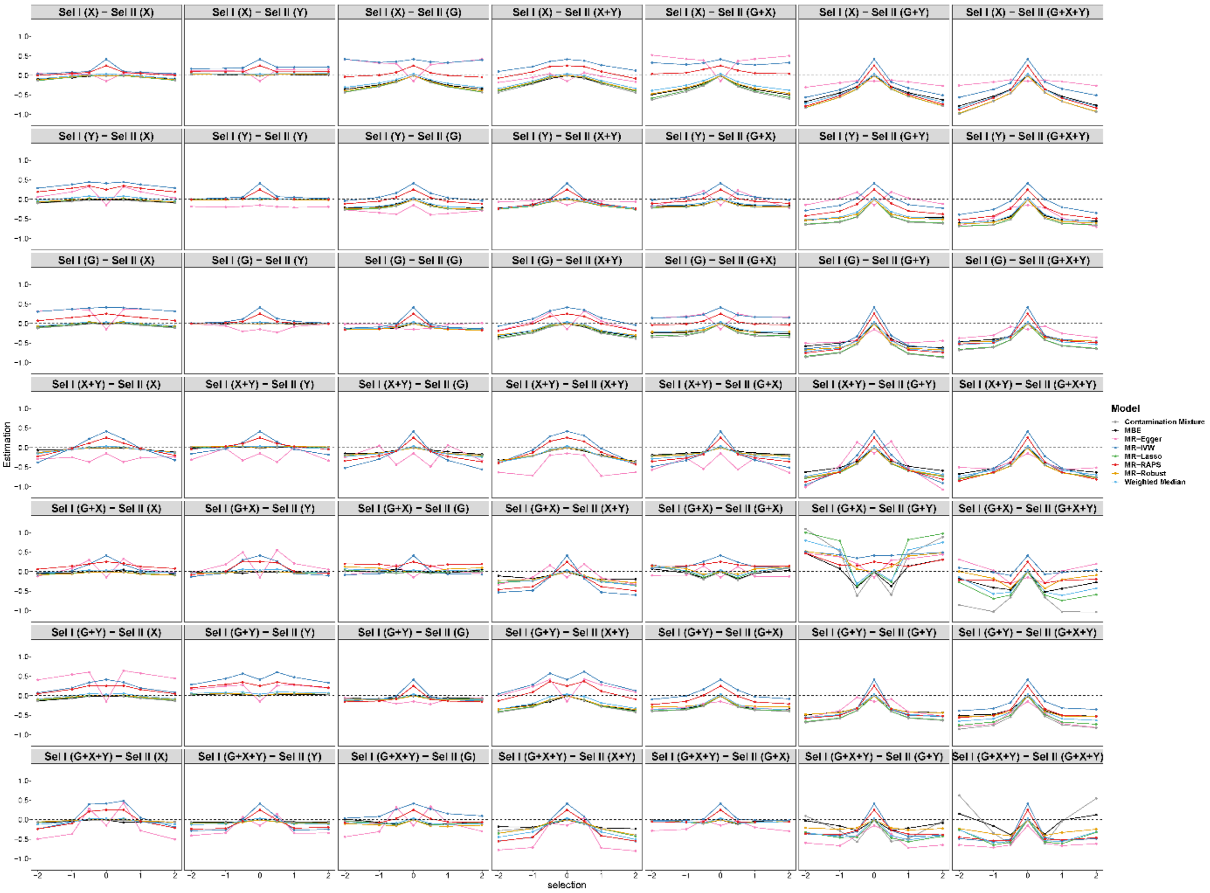

Fig A. Simulation results for causal estimations of eight Pleiotropy-robust MR Methods varying across selection effect from -2 to 2 under different selection mechanisms with Null causal effect in scenario 3 (30% invalid variants, 50 variants).

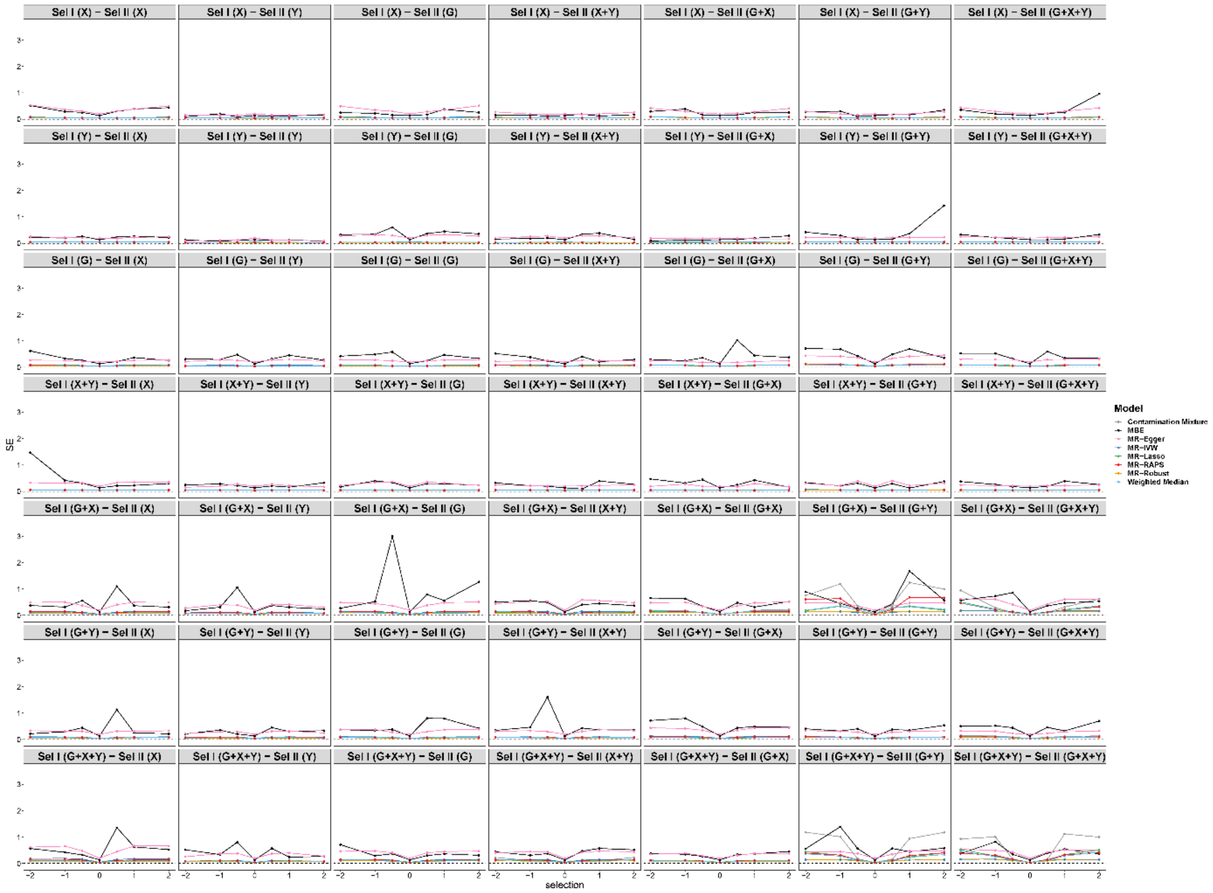

Fig B. Simulation results for SEs of eight Pleiotropy-robust MR Methods varying across selection effect from -2 to 2 under different selection mechanisms with Null causal effect in scenario 3 (30% invalid variants, 50 genetic variants).

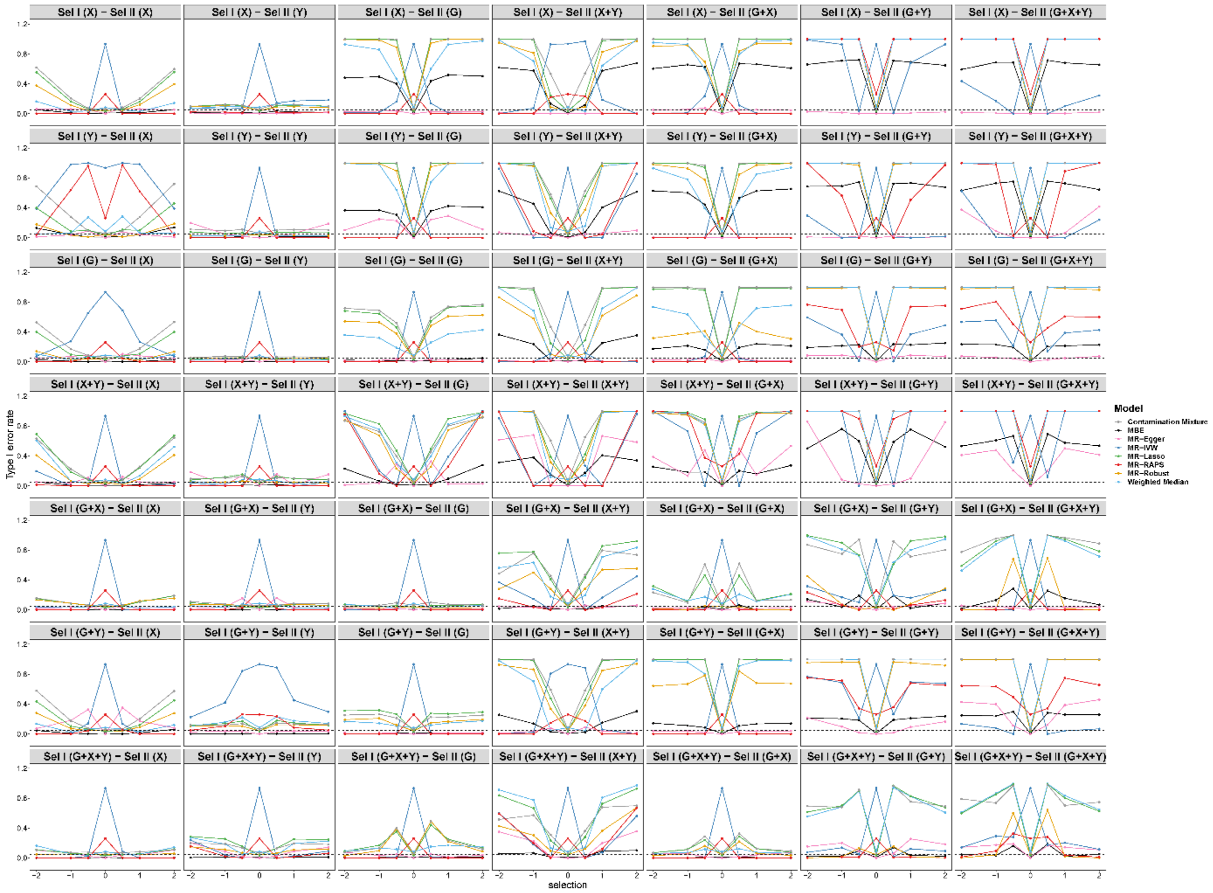

Fig C. Simulation results for type I error rates of eight Pleiotropy-robust MR Methods varying across selection effect from -2 to 2 under different selection mechanisms with Null causal effect in scenario 3 (30% invalid variants, 50 genetic variants).

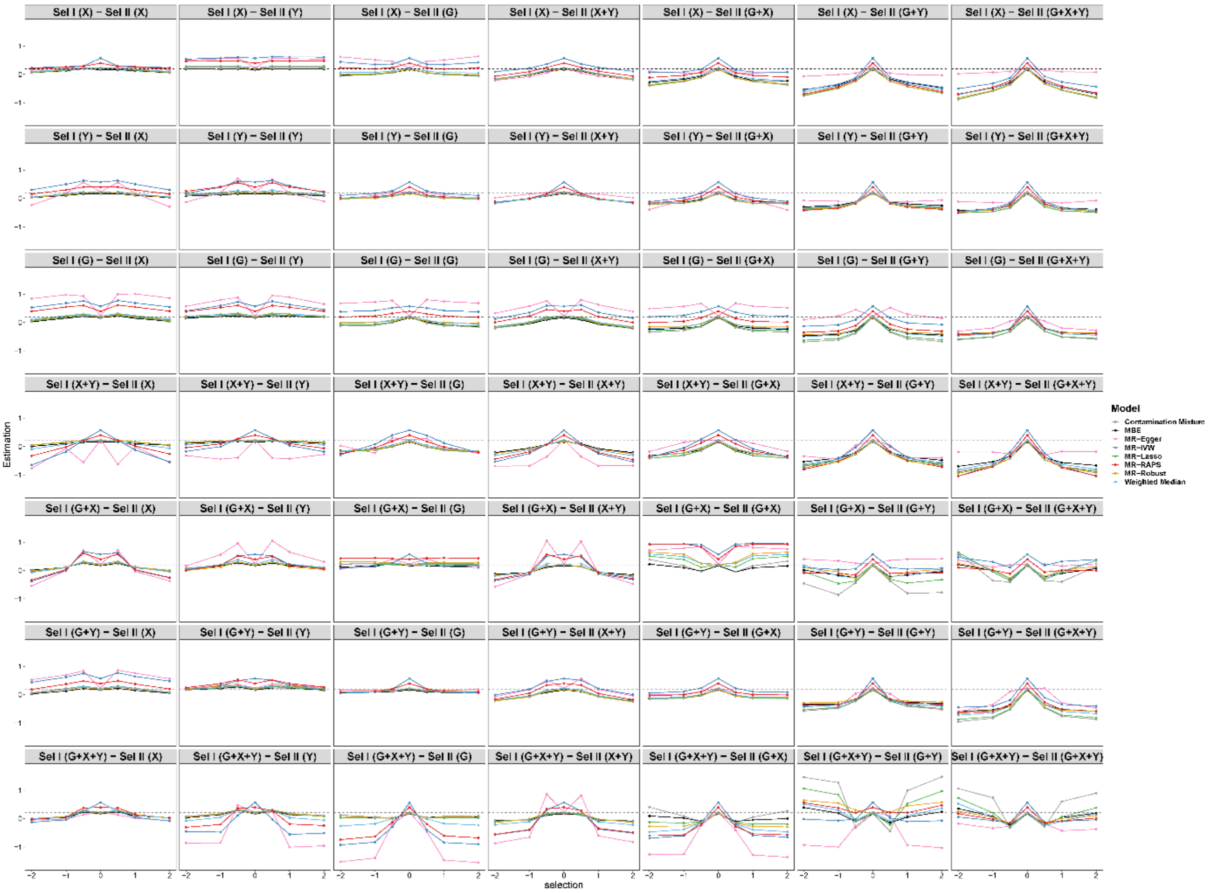

Fig D. Simulation results for causal estimations of eight Pleiotropy-robust MR Methods varying across selection effect from -2 to 2 under different selection mechanisms with Positive causal effect in scenario 3 (30% invalid variants, 50 genetic variants).

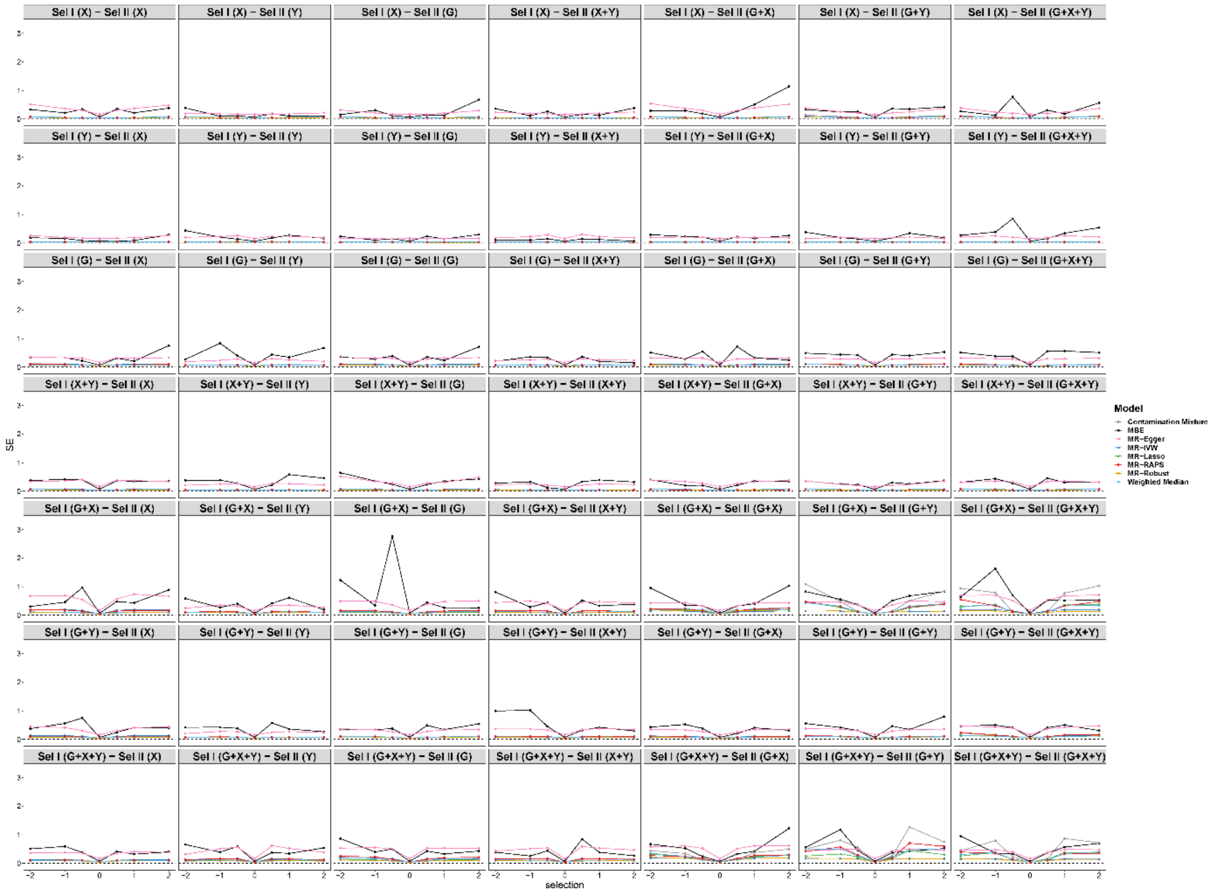

Fig E. Simulation results for SEs of eight Pleiotropy-robust MR Methods varying across selection effect from -2 to 2 under different selection mechanisms with Positive causal effect in scenario 3 (30% invalid variants, 50 genetic variants).

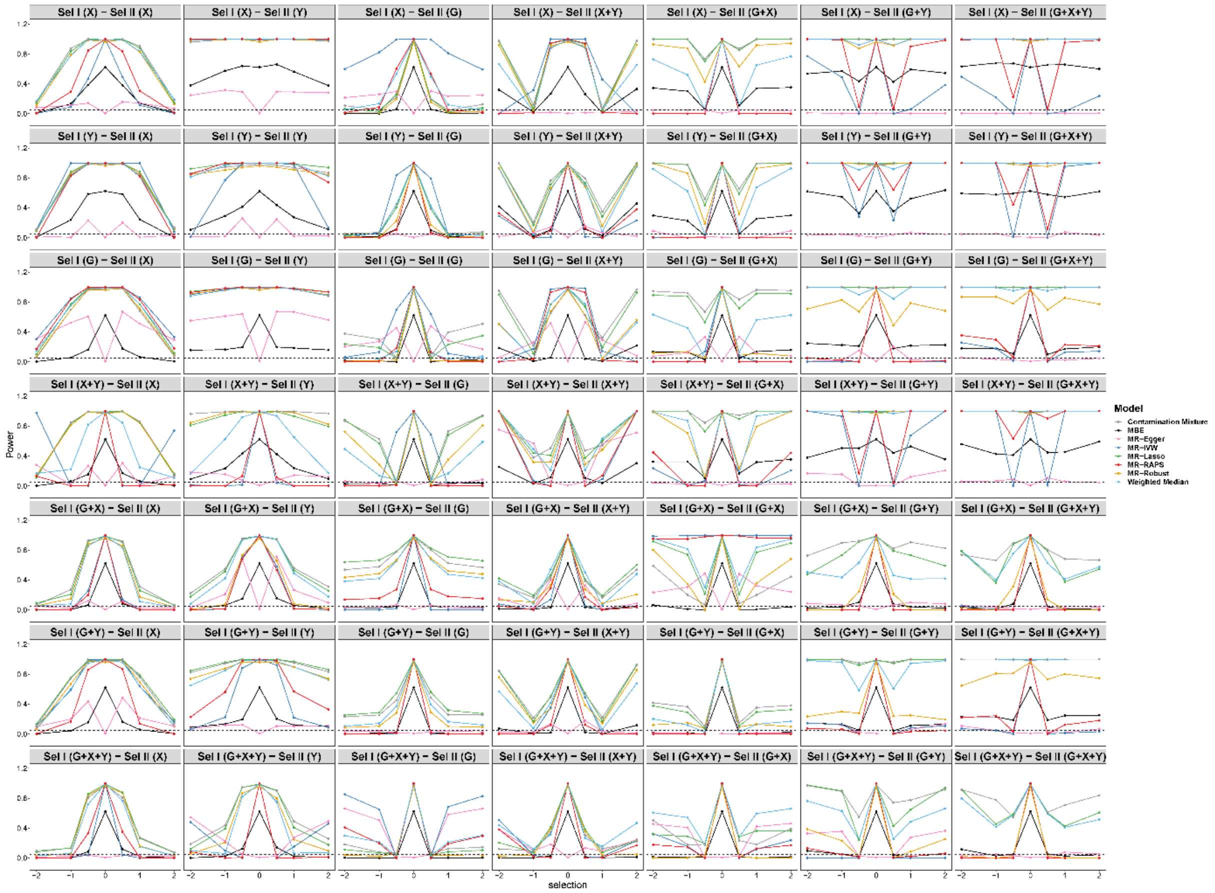

Fig F. Simulation results for statistic power of eight Pleiotropy-robust MR Methods varying across selection effect from -2 to 2 under different selection mechanisms with Positive causal effect in scenario 3 (30% invalid variants, 50 genetic variants).

Figs G-L (30% invalid variants, 100 variants), Figs M-R (70% invalid variants, 50 variants) and Figs S-X (70% invalid variants, 100 variants) show the tendency of estimations, SEs, type I error rates and statistic power under different selection mechanisms and simulation situations when varying across selection effects of *X*, *Y* or *G* on selection (*S*) in scenario 3 (Directional pleiotropy, InSIDE satisfied). In summary, the biases of all models increases with proportion of invalid variants increasing.

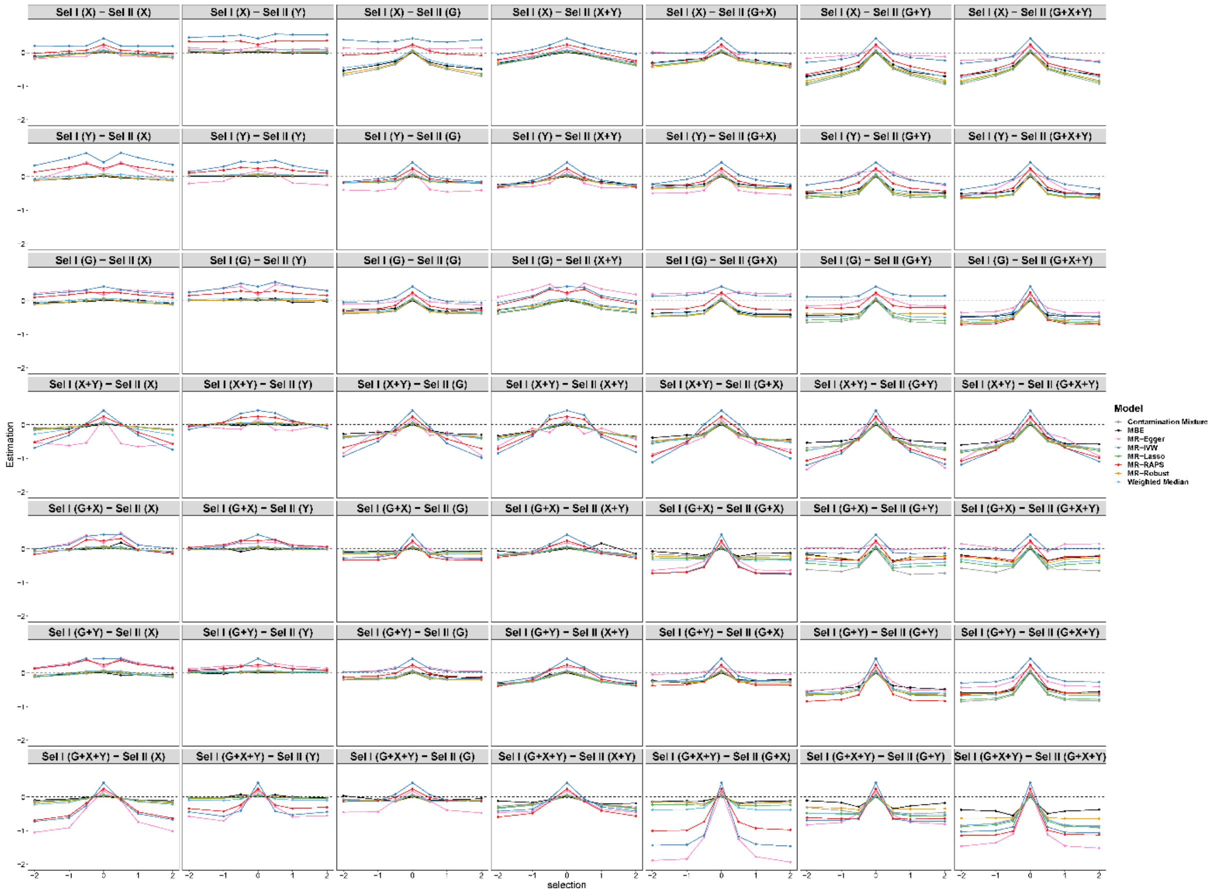

Fig G. Simulation results for causal estimations of eight Pleiotropy-robust MR Methods varying across selection effect from -2 to 2 under different selection mechanisms with Null causal effect in scenario 3 (30% invalid variants, 100 variants).

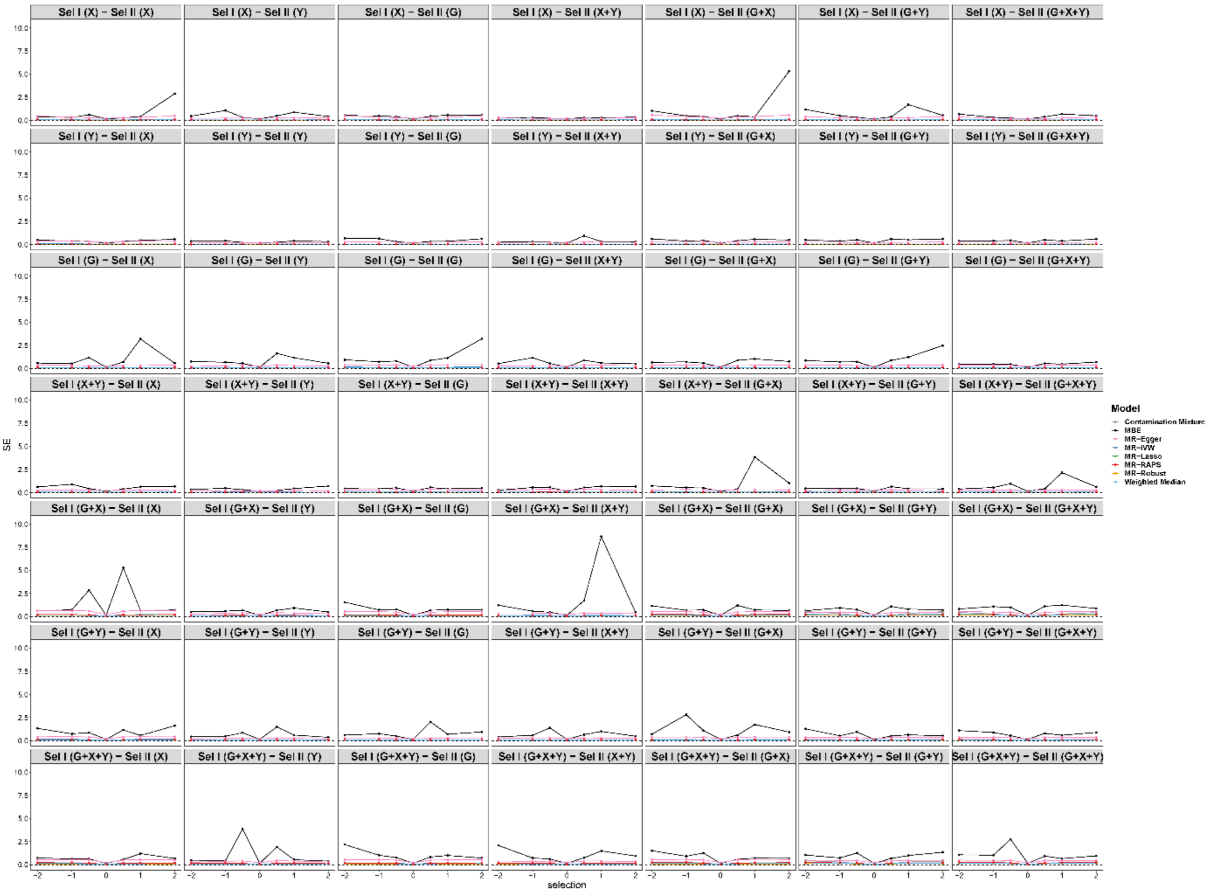

Fig H. Simulation results for SEs of eight Pleiotropy-robust MR Methods varying across selection effect from -2 to 2 under different selection mechanisms with Null causal effect in scenario 3 (30% invalid variants, 100 genetic variants).

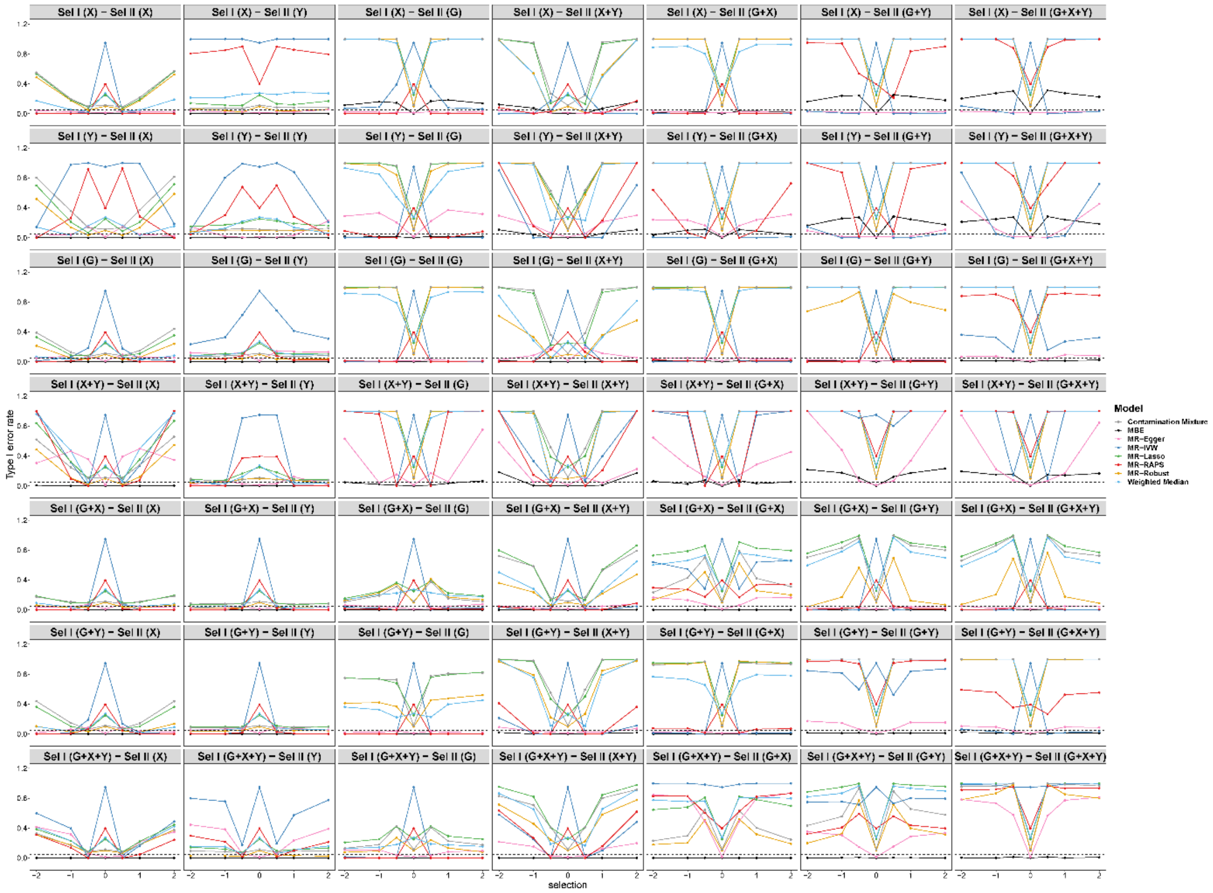

Fig I. Simulation results for type I error rates of eight Pleiotropy-robust MR Methods varying across selection effect from -2 to 2 under different selection mechanisms with Null causal effect in scenario 3 (30% invalid variants, 100 genetic variants).

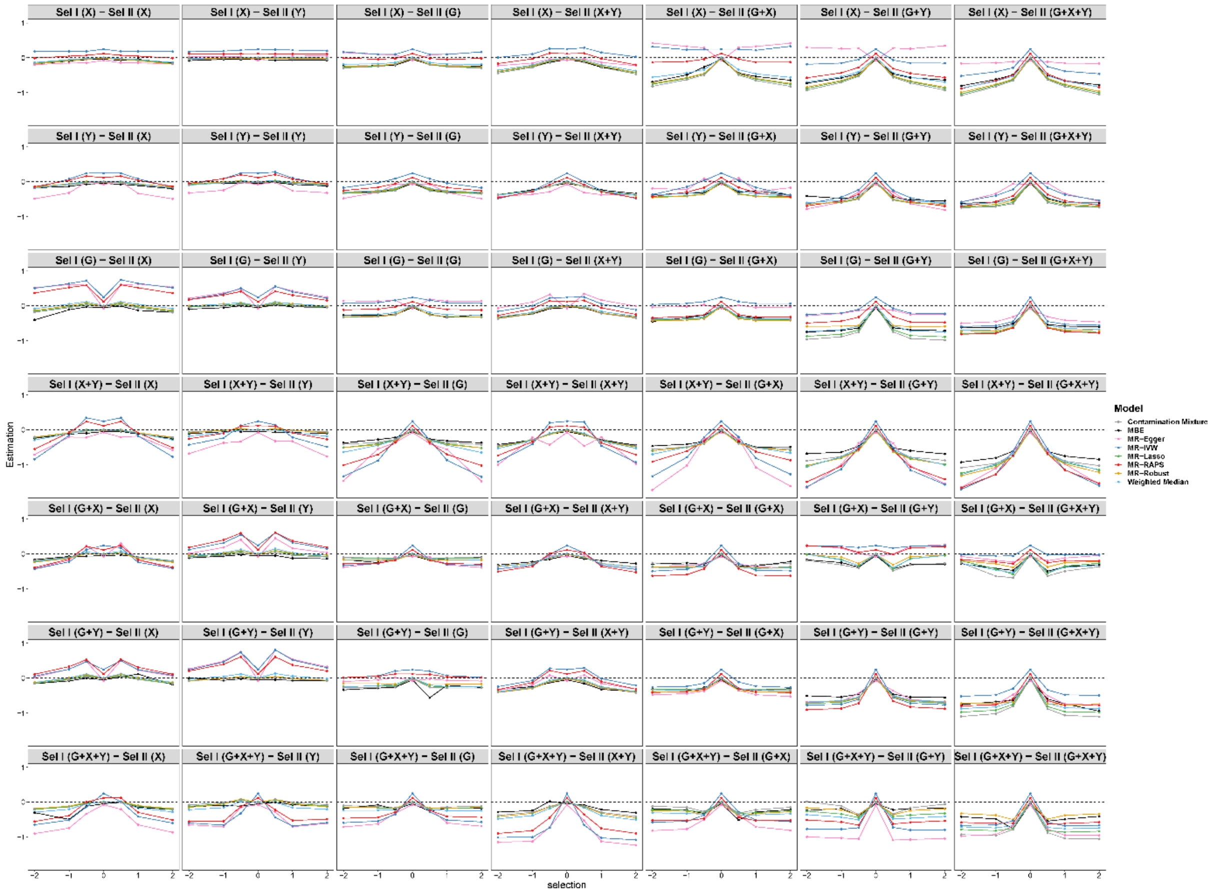

Fig J. Simulation results for causal estimations of eight Pleiotropy-robust MR Methods varying across selection effect from -2 to 2 under different selection mechanisms with Positive causal effect in scenario 3 (30% invalid variants, 100 genetic variants).

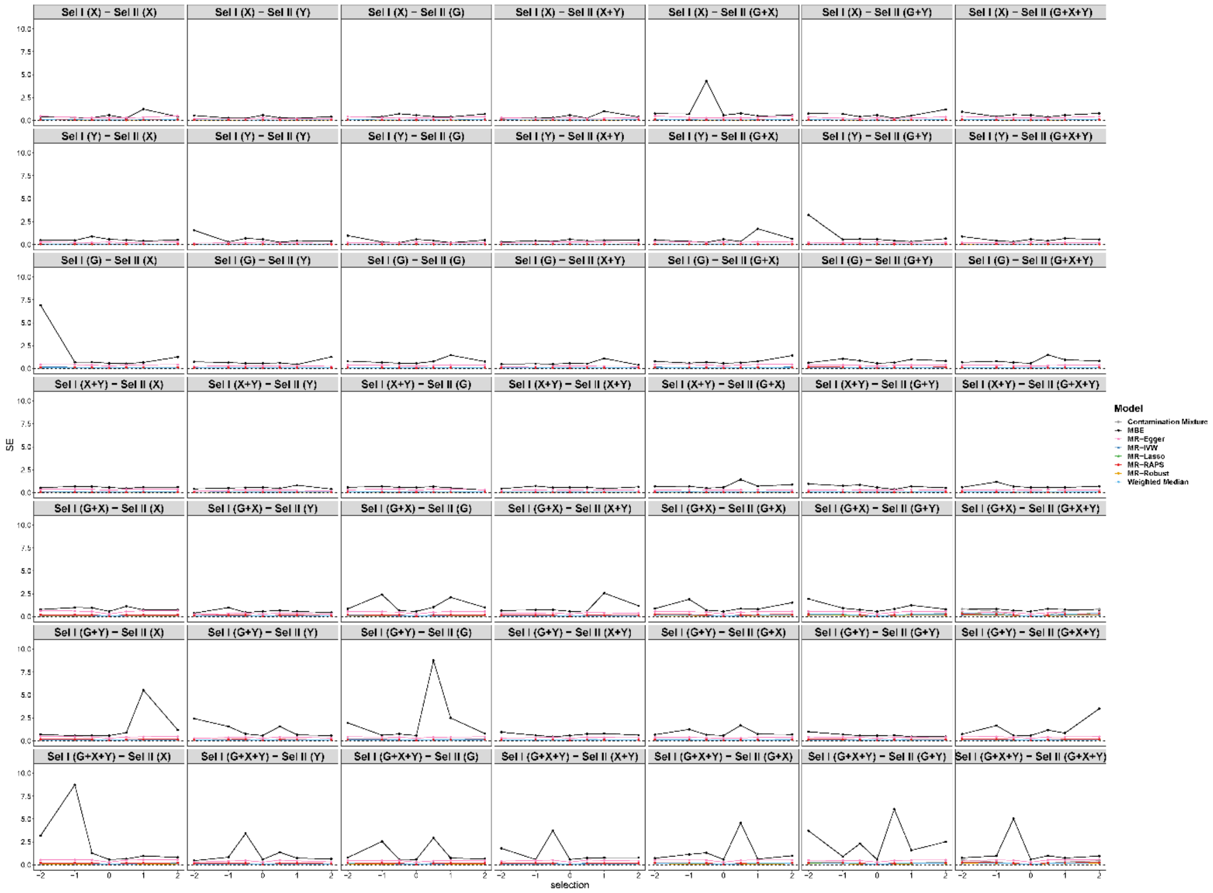

Fig K. Simulation results for SEs of eight Pleiotropy-robust MR Methods varying across selection effect from -2 to 2 under different selection mechanisms with Positive causal effect in scenario 3 (30% invalid variants, 100 genetic variants).

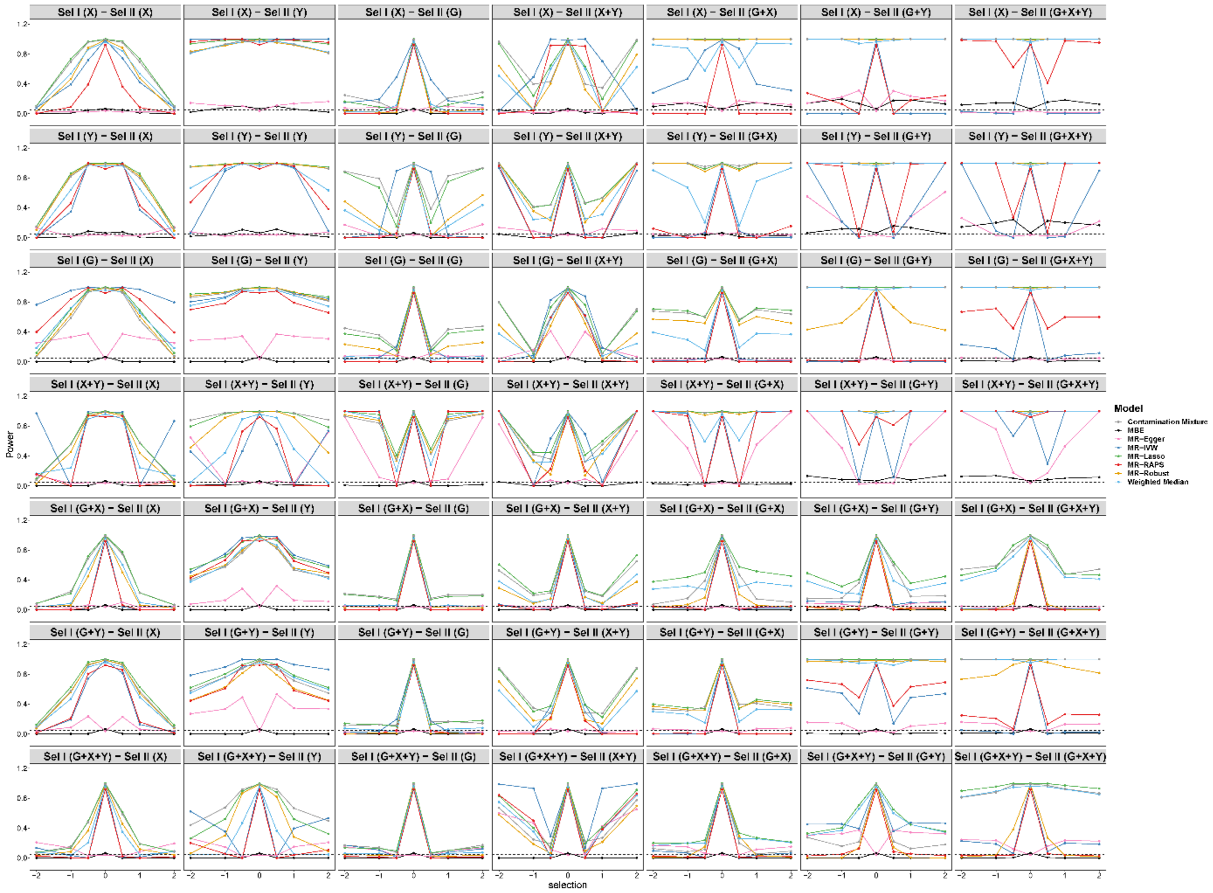

Fig L. Simulation results for statistic power of eight Pleiotropy-robust MR Methods varying across selection effect from -2 to 2 under different selection mechanisms with Positive causal effect in scenario 3 (30% invalid variants, 100 genetic variants).

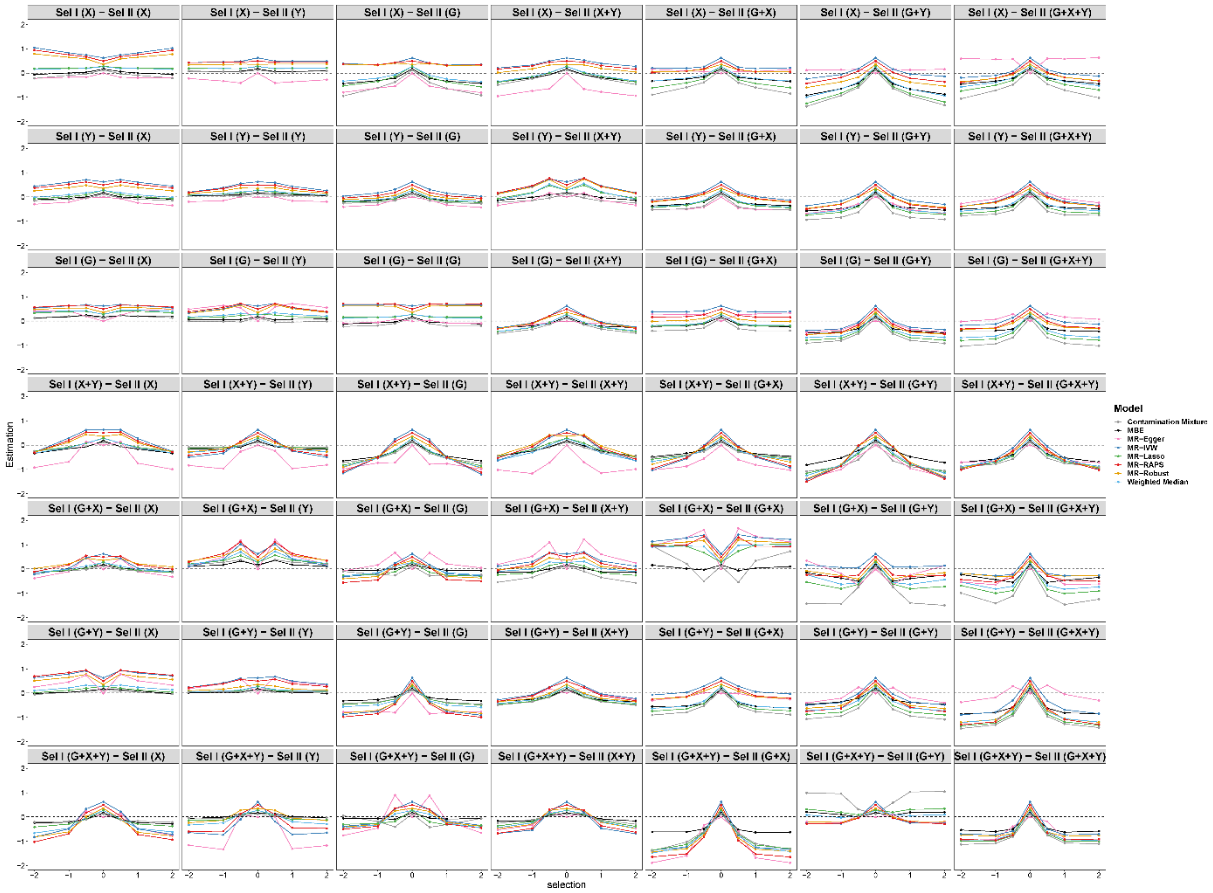

Fig M. Simulation results for causal estimations of eight Pleiotropy-robust MR Methods varying across selection effect from -2 to 2 under different selection mechanisms with Null causal effect in scenario 3 (70% invalid variants, 50 variants).

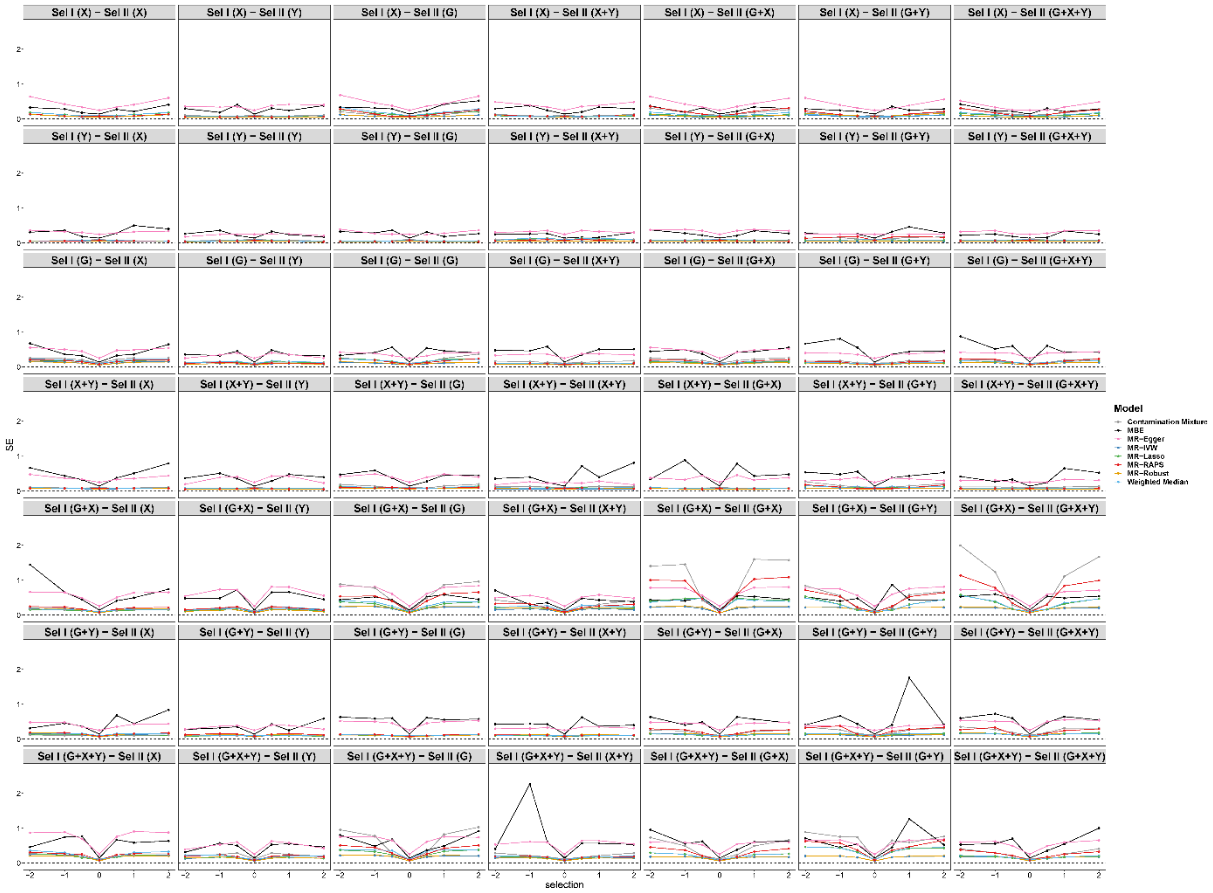

Fig N. Simulation results for SEs of eight Pleiotropy-robust MR Methods varying across selection effect from -2 to 2 under different selection mechanisms with Null causal effect in scenario 3 (70% invalid variants, 50 genetic variants).

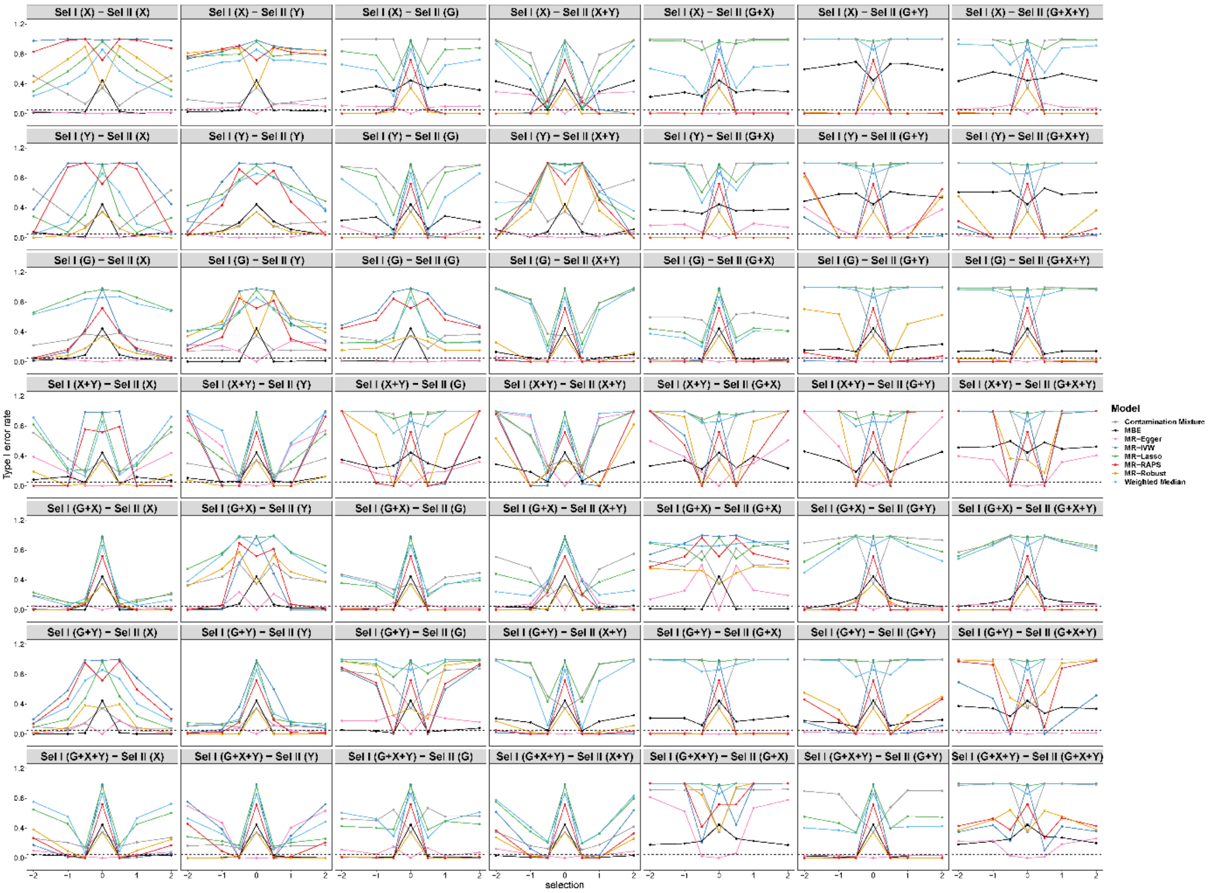

Fig O. Simulation results for type I error rates of eight Pleiotropy-robust MR Methods varying across selection effect from -2 to 2 under different selection mechanisms with Null causal effect in scenario 3 (70% invalid variants, 50 genetic variants).

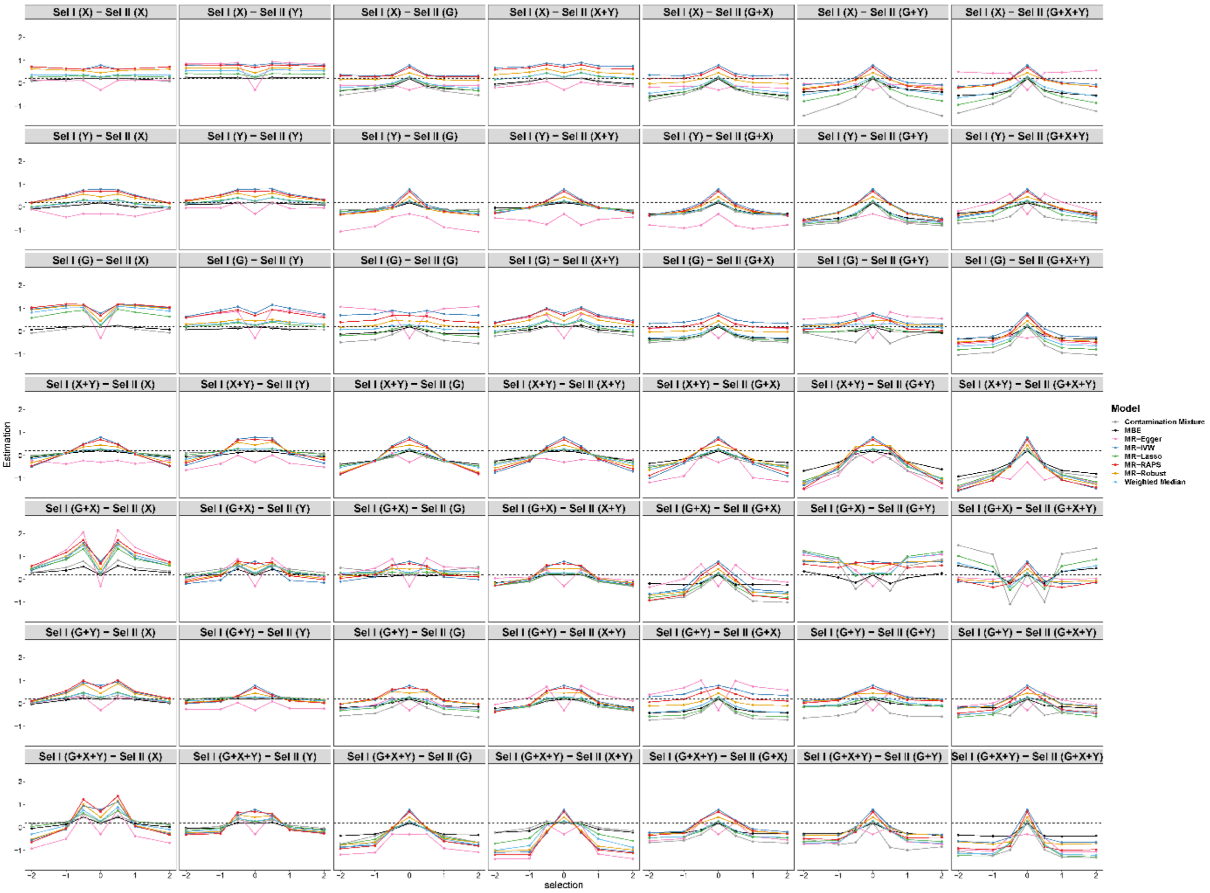

Fig P. Simulation results for causal estimations of eight Pleiotropy-robust MR Methods varying across selection effect from -2 to 2 under different selection mechanisms with Positive causal effect in scenario 3 (70% invalid variants, 50 genetic variants).

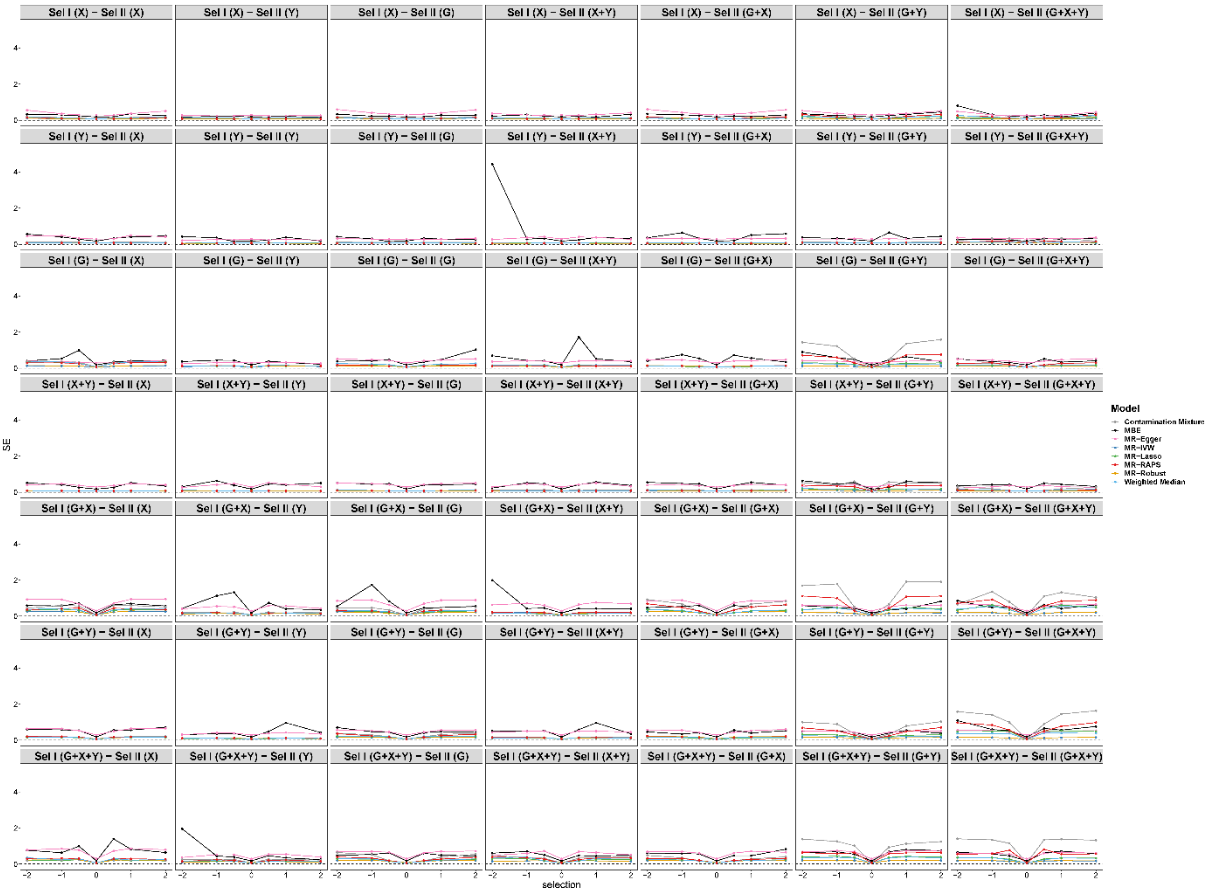

Fig Q. Simulation results for SEs of eight Pleiotropy-robust MR Methods varying across selection effect from -2 to 2 under different selection mechanisms with Positive causal effect in scenario 3 (70% invalid variants, 50 genetic variants).

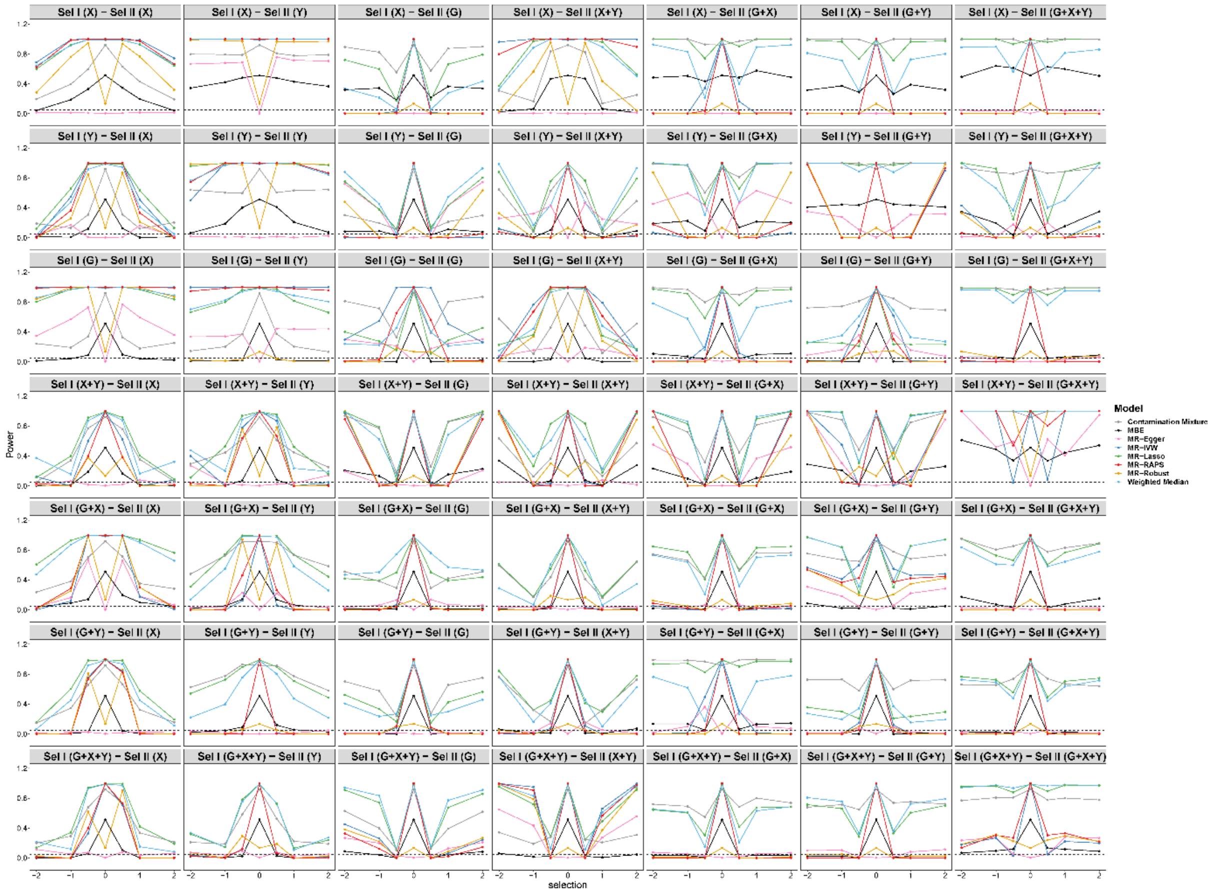

Fig R. Simulation results for statistic power of eight Pleiotropy-robust MR Methods varying across selection effect from -2 to 2 under different selection mechanisms with Positive causal effect in scenario 3 (70% invalid variants, 50 genetic variants).

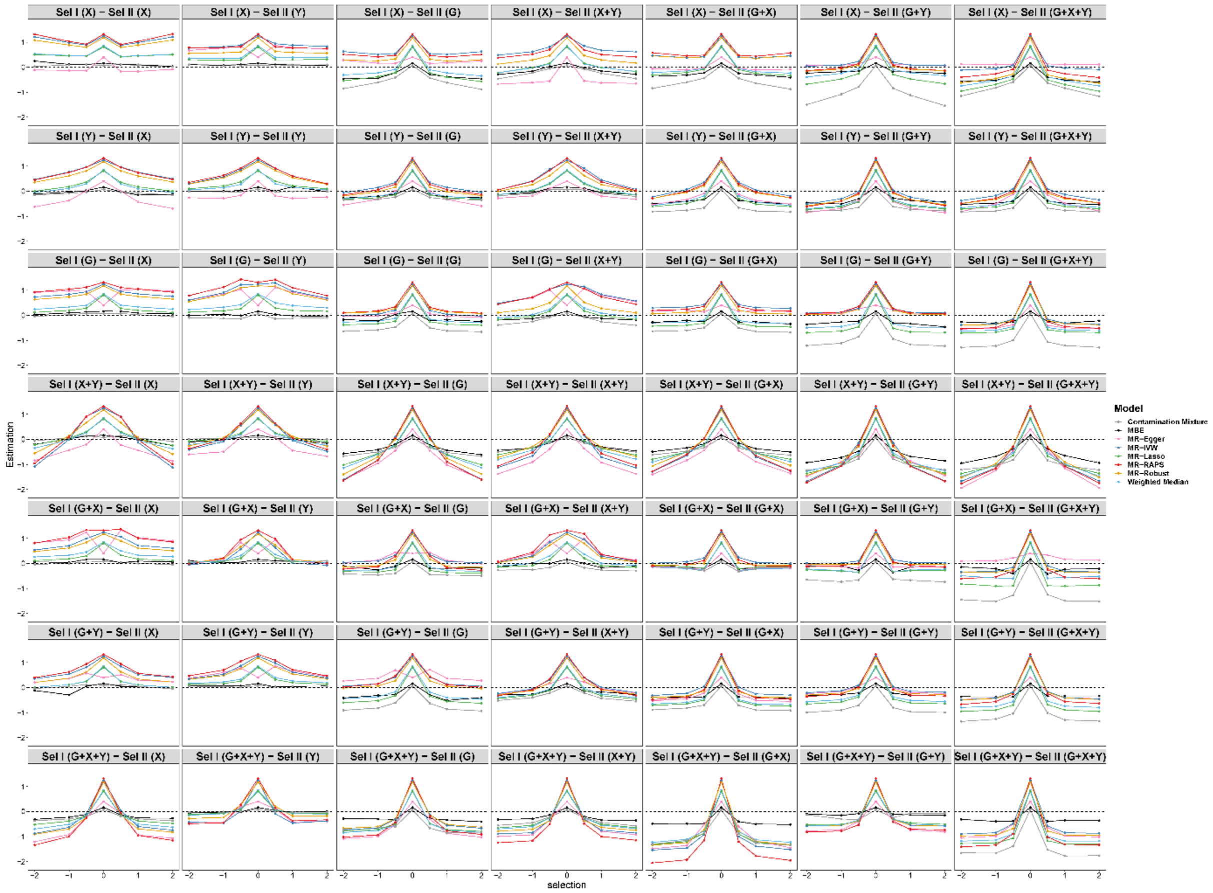

Fig S. Simulation results for causal estimations of eight Pleiotropy-robust MR Methods varying across selection effect from -2 to 2 under different selection mechanisms with Null causal effect in scenario 3 (70% invalid variants, 100 variants).

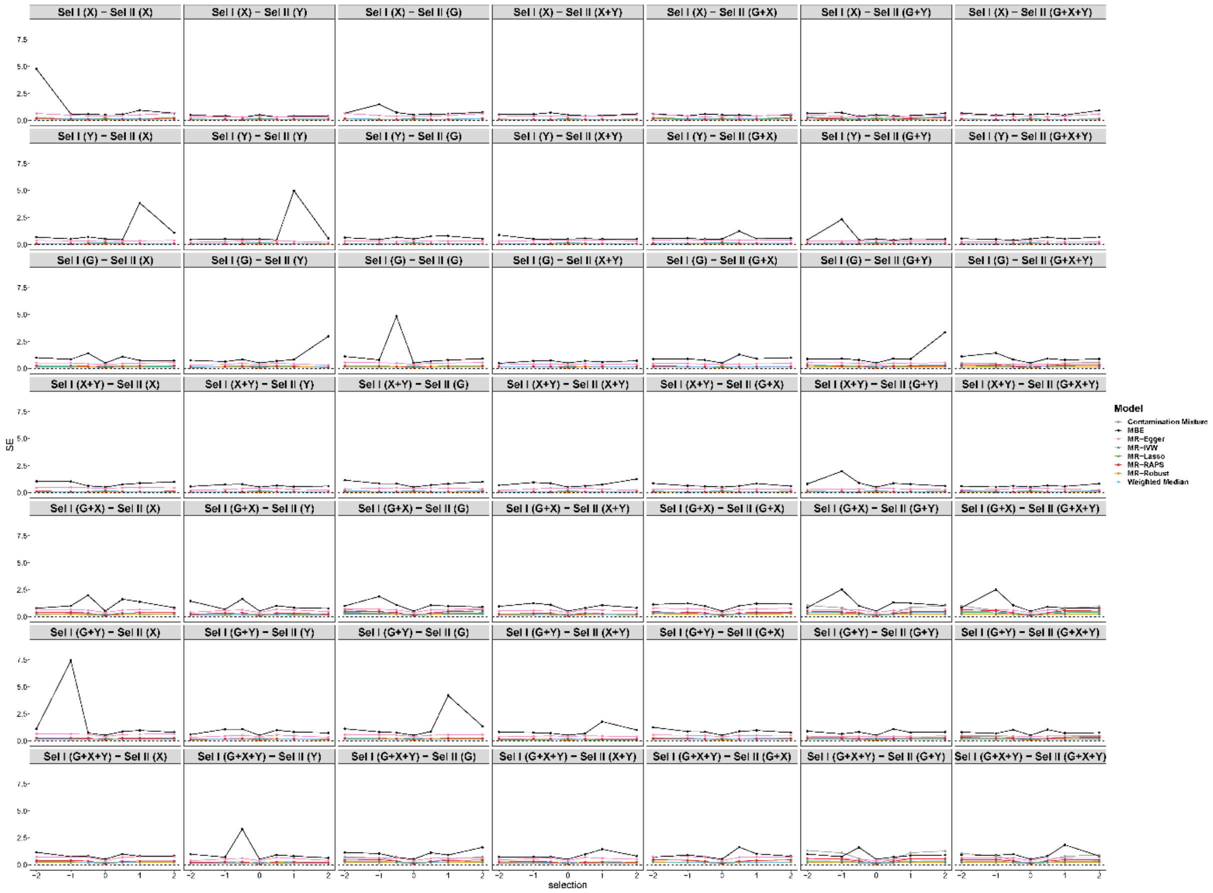

Fig T. Simulation results for SEs of eight Pleiotropy-robust MR Methods varying across selection effect from -2 to 2 under different selection mechanisms with Null causal effect in scenario 3 (70% invalid variants, 100 genetic variants).

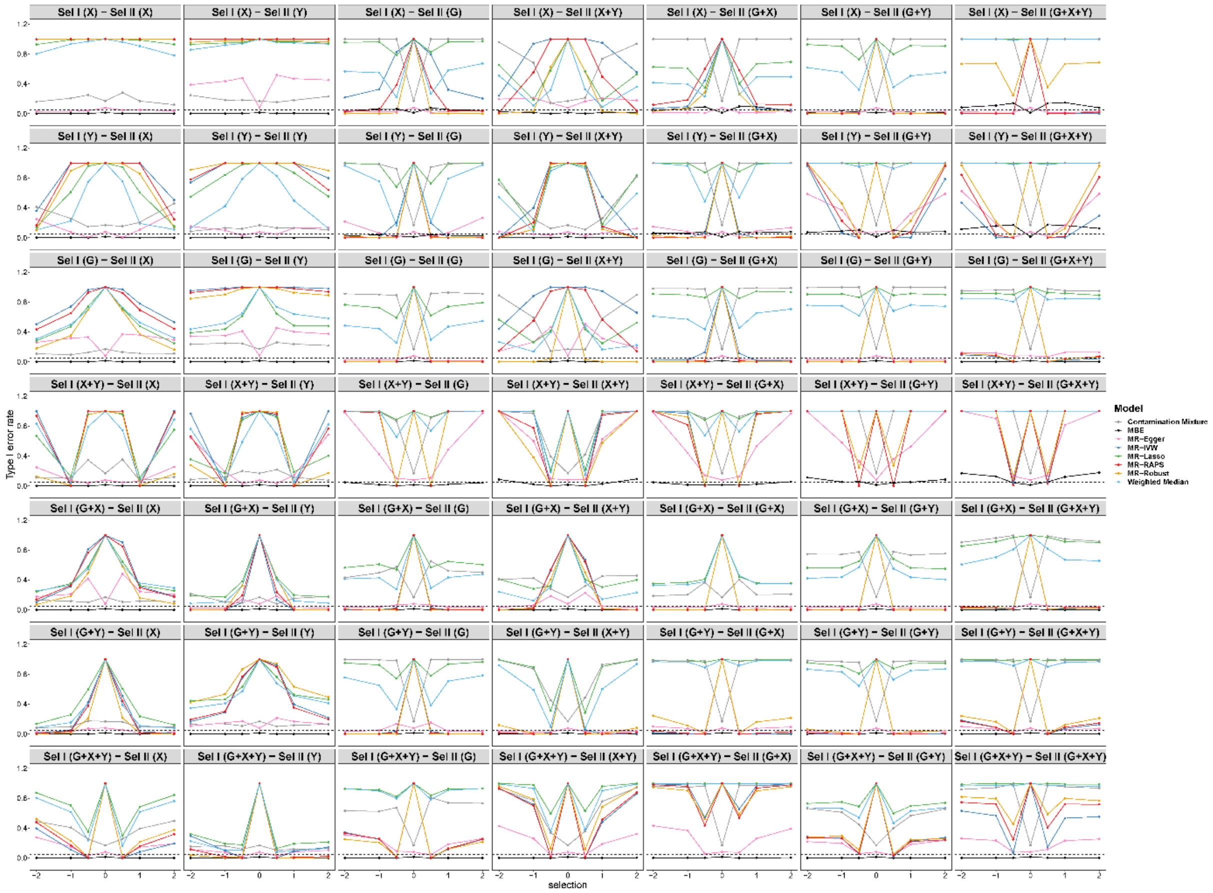

Fig U. Simulation results for type I error rates of eight Pleiotropy-robust MR Methods varying across selection effect from -2 to 2 under different selection mechanisms with Null causal effect in scenario 3 (70% invalid variants, 100 genetic variants).

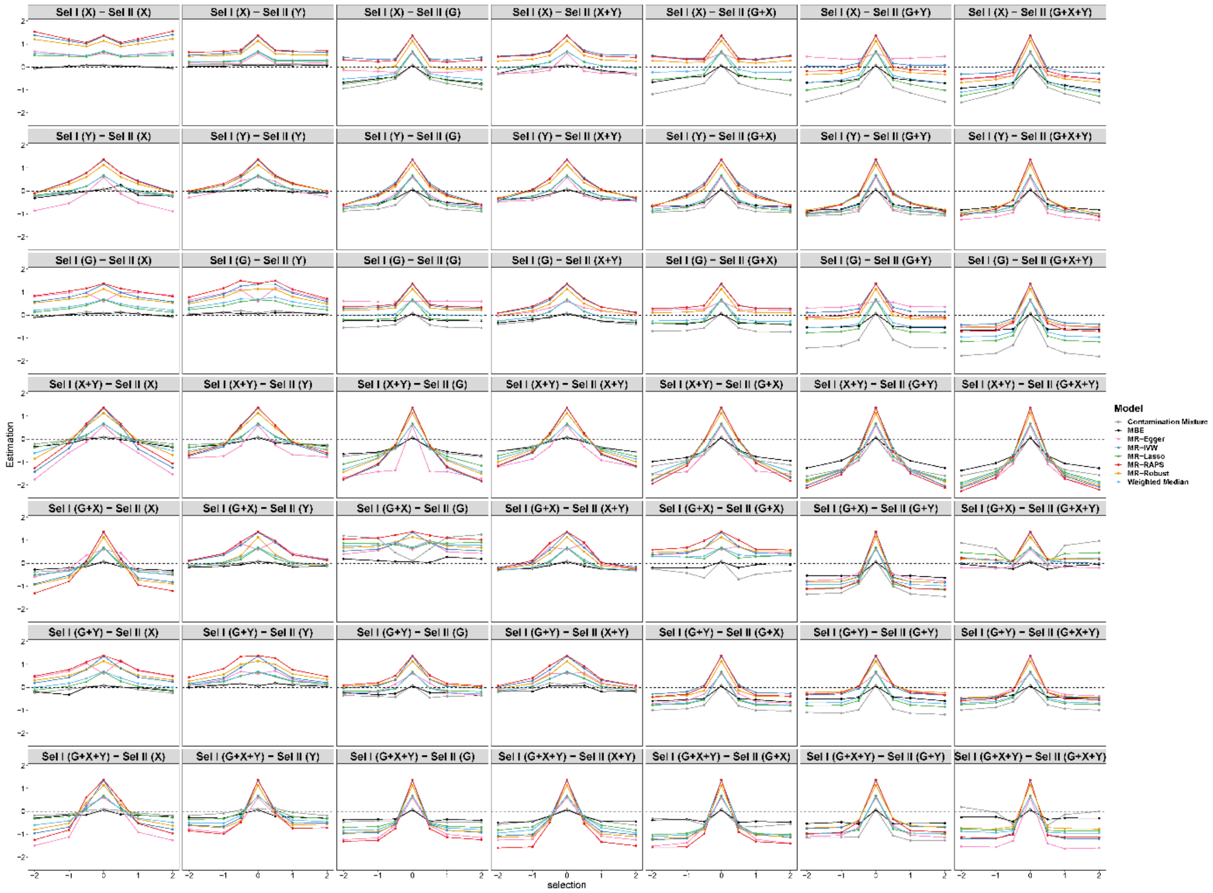

Fig V. Simulation results for causal estimations of eight Pleiotropy-robust MR Methods varying across selection effect from -2 to 2 under different selection mechanisms with Positive causal effect in scenario 3 (70% invalid variants, 100 genetic variants).

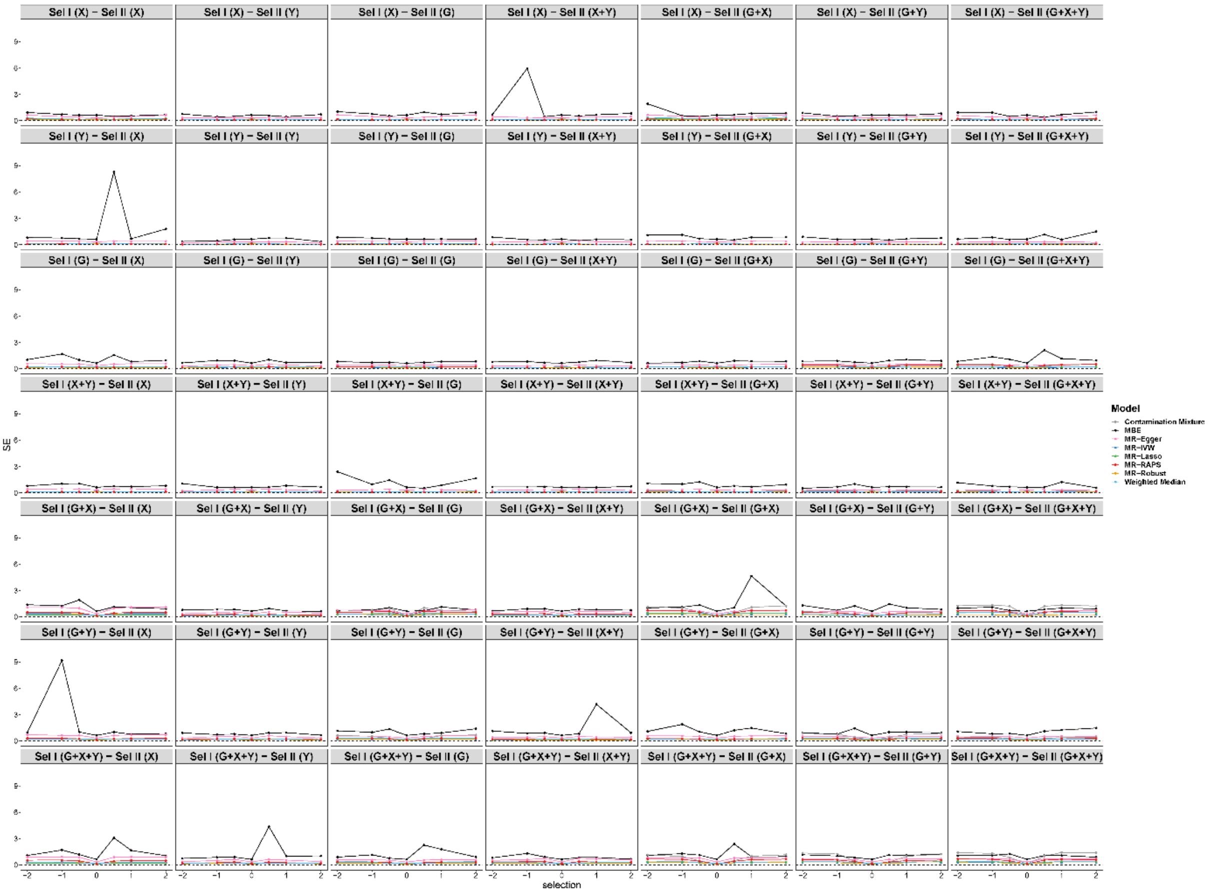

Fig W. Simulation results for SEs of eight Pleiotropy-robust MR Methods varying across selection effect from -2 to 2 under different selection mechanisms with Positive causal effect in scenario 3 (70% invalid variants, 100 genetic variants).

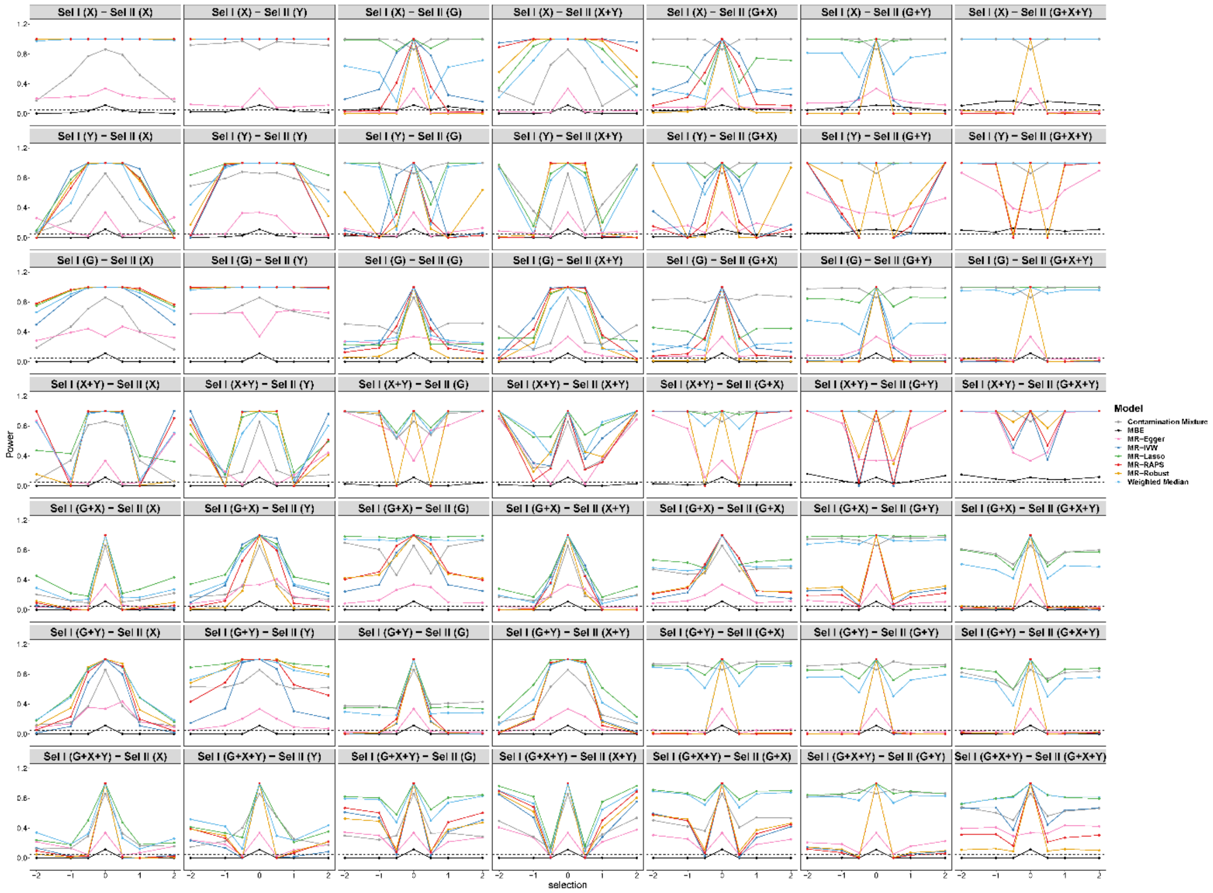

Fig X. Simulation results for statistic power of eight Pleiotropy-robust MR Methods varying across selection effect from -2 to 2 under different selection mechanisms with Positive causal effect in scenario 3 (70% invalid variants, 100 genetic variants).
