# Supplementary material for: Impact of nonrandom selection mechanisms on the causal effect estimation for two-sample Mendelian randomization methods
Source: PLoS Genet. 2022 Mar 17;18(3):e1010107. doi: 10.1371/journal.pgen.1010107 (PMC8963545; doi:10.1371/journal.pgen.1010107)
Supplement: S9 Text — (PDF) [file pgen.1010107.s009.pdf]

S9 Text

Code to implement the method and reproduce all simulations and analyses

```
library("MASS")
library("penalized")
library("R.utils")


MeanF <- function(data){
   resul <- mean(data,na.rm = T)
   return(resul)
}
SDF <- function(data){
   resul <- sd(data,na.rm = T)
   return(resul)
}
SumF <- function(data){
   resul <- sum(data,na.rm = T)
   return(resul)
}



#define function for MR-Lasso with heterogeneity criterion
MR_lasso<-function(betaYG,betaXG,sebetaYG){

   betaYGw = betaYG/sebetaYG # dividing the association estimates by  sebetaYG is equivalent
   betaXGw = betaXG/sebetaYG # to weighting by sebetaYG^-2
   pleio = diag(rep(1, length(betaXG)))
   l1grid = c(seq(from=0.1, to=5, by=0.1), seq(from=5.2, to=10, by=0.2))
   # values of lambda for grid search
   l1grid_rse = NULL; l1grid_length = NULL; l1grid_beta = NULL;
   l1grid_se = NULL
   for (i in 1:length(l1grid)) {
      l1grid_which = which(attributes(penalized(betaYGw, pleio,
                                                    betaXGw,           lambda1=l1grid[i],
trace=FALSE))$penalized==0)
      l1grid_rse[i]        =        summary(lm(betaYG[l1grid_which]~betaXG[l1grid_which]-1,
weights=sebetaYG[l1grid_which]^-2))$sigma
      l1grid_length[i] = length(l1grid_which)
      l1grid_beta[i] = lm(betaYG[l1grid_which]~betaXG[l1grid_which]-1,
```

```
                                weights=sebetaYG[l1grid_which]^-2)$coef[1]
    l1grid_se[i]        =       summary(lm(betaYG[l1grid_which]~betaXG[l1grid_which]-1,
weights=sebetaYG[l1grid_which]^-2))$coef[1,2]/
      min(summary(lm(betaYG[l1grid_which]~betaXG[l1grid_which]-
1,weights=sebetaYG[l1grid_which]^-2))$sigma, 1)

  }
  l1which_hetero = c(which(l1grid_rse[1:(length(l1grid)-1)]>1& diff(
    l1grid_rse)>qchisq(0.95, df=1)/l1grid_length[2:length(l1grid)])
    , length(l1grid))[1]
  # heterogeneity criterion for choosing lambda

  l1hetero_beta = l1grid_beta[l1which_hetero]

  l1hetero_se = l1grid_se[l1which_hetero]
  list(ThetaEstimate=l1hetero_beta, ThetaSE=l1hetero_se )

}

DateGenerate <- function(a,b,d,c,ex,ey,eg,n,seln,ua,ya,sel){
  g <- length(a)

  G <- NULL
  for(i in 1:g){
    G <- cbind(G,rbinom(n,2,0.3))
  }
  new_G <- G

  colnames(new_G) <- paste0('G',1:g)

  U <- new_G %*% ua+ rnorm(n,0,1)
  x <- new_G %*% a + b*U + rnorm(n,0,1)
  y <- d*x + c*U + rnorm(n,0,1) + new_G %*% ya

  e <- sel*c(ex,ey,eg)
  qq <- summary(ceiling(e[1]*x+e[2]*y+apply(e[3]*new_G,1,SumF)))[3]
  qq <- strsplit(qq,':')[[1]][2]
  qq <- as.numeric(qq)
  ps              <-              exp(e[1]*x+e[2]*y+apply(e[3]*new_G,1,SumF)-
qq)/(1+exp(e[1]*x+e[2]*y+apply(e[3]*new_G,1,SumF)-qq))

  s <- rbinom(n,1,ps)

  data <- data.frame(new_G,x,y,s)
```

```
if (e[1]!=0|e[2]!=0|e[3]!=0){
    selsamp <- data[data$s==1,]
}else{
    selsamp <- data
}

samdata <- selsamp[sample(1:dim(selsamp)[1],seln,replace = F),]



formulaR2 <- paste0('x~G1')

for(iform in 2:g){
    formulaR2 <- paste0(formulaR2,'+',colnames(samdata)[iform])
}



formulaR2 <- as.formula(formulaR2)
R2 <- lm(formulaR2,data=samdata)
R2_out <- summary(R2)$r.squared
betaXG <- rep(NA,g)
betaYG <- rep(NA,g)
SEXG <- rep(NA,g)
SEYG <- rep(NA,g)

for(j in 1:g){

    modelxG <- lm(samdata$x~samdata[,j])



    betaXG [j] <- summary(modelxG)$coefficients[2,1]
    SEXG [j] <- summary(modelxG)$coefficients[2,2]



    modelYG <- lm(samdata$y~samdata[,j])



    betaYG [j] <- summary(modelYG)$coefficients[2,1]
    SEYG [j] <- summary(modelYG)$coefficients[2,2]

}



end_data <- data.frame(betaXG=betaXG,
                                             betaYG=betaYG,
```

```
                                    SEXG=SEXG,
                                    SEYG=SEYG,
                                    R2=R2_out)
   return(end_data)

}

Method_CP <- function(bx, bxse, by, byse){

   result <- list()

   mr_frame<-as.data.frame(cbind(bx,bxse,by,byse))
   names(mr_frame)<- c('bx','bxse','by','byse')
   mr_object<- MendelianRandomization::mr_input(bx, bxse, by, byse)

   #perform weighted median
   result[['weighted median']] <- MendelianRandomization::mr_median(mr_object, seed = NA)

   #perform Mode based estimation
   result[['Mode based estimation']] <- MendelianRandomization::mr_mbe(mr_object, weighting =
"weighted", stderror = "delta",
                                                                                    phi = 1,
seed = NA, iterations = 2000,

distribution = "normal", alpha = 0.05)

   #perform MR-Robust
   result[['MR-Robust']] <- MendelianRandomization::mr_ivw(mr_object,"random", robust =
TRUE)

   #perform MR-Egger
   result[['MR-Egger']] <- MendelianRandomization::mr_egger(mr_object)

   #perform MR-IVW
   result[['MR-IVW']] <- MendelianRandomization::mr_ivw(mr_object)



   #perform MR-Lasso
   result[['MR-Lasso']] <- MR_lasso(by,bx,byse)

   result[['contamination mixture']] <- MendelianRandomization::mr_conmix(mr_object)

   result[['MR-RAPS']] <- TwoSampleMR::mr_raps(bx, by, bxse, byse)
```

```
    return(result)

}


Simulation <-
function(a=a,b=b,d=d,c=c,ex=ex,ey=ey,eg=eg,n=n,seln=seln,ua=ua,ya=ya,sel1=sel1,sel2=sel2,Two_S=Two_S){

    data1 <- DateGenerate (a,b,d,c,ex,ey,eg,n,seln,ua,ya,sel=sel1)
    data2 <- DateGenerate (a,b,d,c,ex,ey,eg,n,seln,ua,ya,sel=sel2)
    if(Two_S==T){
        bx=data1$betaXG
        bxse=data1$SEXG
        by=data2$betaYG
        byse=data2$SEYG
    }else{
        bx=data1$betaXG
        bxse=data1$SEXG
        by=data1$betaYG
        byse=data1$SEYG
    }
    result <- Method_CP (bx=bx, bxse=bxse, by=by, byse=byse)
    name <- names(result)

    result1 <- c(result$`weighted median`@Estimate,result$`weighted median`@StdError)
    names(result1) <- paste0(name[1],c(' Estimate',' StdError'))

    CI1 <- c(result$`weighted median`@CILower,result$`weighted median`@CIUpper)
    if(CI1[1] <0 & CI1[2]>0){
        power1 <- 0
    }else{
        power1 <- 1
    }

    result2 <- c(result$`Mode based estimation`@Estimate,result$`Mode based
estimation`@StdError)
    names(result2) <- paste0(name[2],c(' Estimate',' StdError'))


    CI2 <- c(result$`Mode based estimation`@CILower,result$`Mode based estimation`@CIUpper)
    if(CI2[1] <0 & CI2[2]>0){
        power2 <- 0
```

```
}else{
   power2 <- 1
}

result3 <- c(result$`MR-Robust`@Estimate,result$`MR-Robust`@StdError)
names(result3) <- paste0(name[3],c(' Estimate',' StdError'))

CI3 <- c(result$`MR-Robust`@CILower,result$`MR-Robust`@CIUpper)
if(CI3[1] <0 & CI3[2]>0){
   power3 <- 0
}else{
   power3 <- 1
}


result4 <- c(result$`MR-Egger`@Estimate,result$`MR-Egger`@StdError.Est)
names(result4) <- paste0(name[4],c(' Estimate',' StdError'))


CI4 <- c(result$`MR-Egger`@CILower.Est,result$`MR-Egger`@CIUpper.Est)
if(CI4[1] <0 & CI4[2]>0){
   power4 <- 0
}else{
   power4 <- 1
}


result5 <- c(result$`MR-IVW`@Estimate,result$`MR-IVW`@StdError)
names(result5) <- paste0(name[5],c(' Estimate',' StdError'))


CI5 <- c(result$`MR-IVW`@CILower,result$`MR-IVW`@CIUpper)
if(CI5[1] <0 & CI5[2]>0){
   power5 <- 0
}else{
   power5 <- 1
}

result7 <- c(result$`MR-Lasso`$ThetaEstimate,result$`MR-Lasso`$ThetaSE)
names(result7) <- paste0(name[7],c(' Estimate',' StdError'))


p7 <- pnorm(result7[1], mean = 0, sd = result7[2], lower.tail = TRUE, log.p = FALSE)
p77 <- 2*min (p7,1-p7)
```

```
  if(p77 < 0.05){
    power7 <- 1
  }else{
    power7 <- 0
  }

  result8 <- c(result$`contamination mixture`@Estimate,result$`contamination mixture`@CILower,
               result$`contamination mixture`@CIUpper)
  names(result8) <- paste0(name[8],c(' Estimate',' Lower','Upper'))


  if(result8[2] <0 & result8[3]>0){
    power8 <- 0
  }else{
    power8 <- 1
  }


  result10 <- c(result$`MR-RAPS`$b,result$`MR-RAPS`$se)
  names(result10) <- paste0(name[9],c(' Estimate',' StdError'))

  p10 <- pnorm(result10[1], mean = 0, sd = result10[2], lower.tail = TRUE, log.p = FALSE)
  p100 <- 2*min (p10,1-p10)
  if(p100 < 0.05){
    power10 <- 1
  }else{
    power10 <- 0
  }


  R2 <- mean(data1$R2)
  names(R2) <- 'R2'


  end_result <- c(result1,result2,result3,result4,result5,
                  result7,result8,result10,R2)
  estimate <- end_result [c(1,3,5,7,9, 11,13,16)]

  power_result <- c(power1,power2,power3,power4,power5,
                    power7,power8,power10)
  names(power_result) <- paste0(name,'power')
  all_all_result <- c(estimate,power_result,end_result[length(end_result)])
  return(all_all_result)
```

```
}


simu_function <-
function(a=a,b=b,d=d,c=c,ex=ex,ey=ey,eg=eg,n=n,seln=seln,sim,ua=ua,ya=ya,sel1=sel1,sel2=sel2
,Two_S=Two_S){

  result_simu_all <- NULL
  for(i in 1:sim){
    cat(i)
    result_simu_qian <- try(withTimeout({Simulation
(a=a,b=b,d=d,c=c,ex=ex,ey=ey,eg=eg,n=n,seln=seln,ua=ua,ya=ya,sel1=sel1,

sel2=sel2,Two_S=Two_S)},timeout=1000,onTimeout="silent"),silent = F)
    if ('try-error' %in% class(result_simu_qian)) {

      next

    }else{
      result_simu <- result_simu_qian
    }
    result_simu_all <- rbind(result_simu_all,result_simu)
  }
  bias <- apply(result_simu_all[,1:8],2,MeanF)-d
  sd <- apply(result_simu_all[,1:8],2,SDF)
  power_r <- apply(result_simu_all[,9:16], 2, MeanF)

  use_name <- names(bias)

  names(bias) <- paste0(use_name,' bias')
  names(sd) <- paste0(use_name,' sd')
  names(power_r) <- paste0(use_name,' power')
  return_reslt <- c(bias,sd,power_r,mean(result_simu_all[,17]))
  names(return_reslt)[25] <- 'R2'


  return(return_reslt)
}
```
