# Supplementary material for: Impact of nonrandom selection mechanisms on the causal effect estimation for two-sample Mendelian randomization methods
Source: PLoS Genet. 2022 Mar 17;18(3):e1010107. doi: 10.1371/journal.pgen.1010107 (PMC8963545; doi:10.1371/journal.pgen.1010107)
Supplement: Attachment — Submitted filename: Yu MR selection.docx [file pgen.1010107.s015.docx]

The authors consider selection bias in two-sample Mendelian randomisation studies, showing unsurprisingly that selection either sample can lead to bias, increased type-1 error and/or loss in power, but also interestingly that selection in the second, outcome sample is the more serious issue. They present two theorems giving sufficient conditions for unbiased estimation in selected samples. They consider a wide range of selection structures, and a good range of currently popular MR methods. This is an interesting study that informs the field.

Comments on the main text:

1. This work is related to the paper of Hartwig et al, Int J Epidemiol 2021, who considered conditioning on covariates rather than sample selection, but which is logically the same problem. Reassuringly, both sets of authors have found that selection in the second sample is the more severe problem. Please discuss the Hartwig et al work.
2. Readers would benefit from more discussion of actual selection mechanisms, so that they know which situations their studies might be reflective of. Please give a more complete list of relevant mechanisms (case/control studies, disease progression, non-representative cohorts etc, and include those where selection on G may occur, since you consider this later) and review extant methods to correct for selection in these situations.
3. L67, refs 5 and 6 are blatant self-references, and not really pertinent to the context. Similarly for ref 7 on L68; however, a relevant reference is needed to back up that statement (eg Paternoster et al 2017, in this journal).
4. L76-79 I didn’t understand these sentences. What collider is Y a descendent of, when there is a causal effect, but not when there isn’t a causal effect?
5. L108 eqn 1 treats G as a random variable, but it was just defined as a set of random variables.
6. L125 Having just discussed the linearity assumption, it is now odd to see discussion of logistic models and odds ratios.
7. L126 “genetic correlation” is the wrong expression.
8. L134 This is the first occurrence of “exchangeability assumption”, previously termed independence assumption. Please use a consistent terminology.
9. L138 S1 and S2 have not been properly defined. So far they are just called the “selection mechanism”, but here they seem to be treated as random variables. Why not define random variables taking the value 1 is a subject is selected into the study? Then the “dot” notation in your subscripts, which is non-standard and odd, could be replaced by the conditioning symbol.
10. In the main text, please direct the reader to the supplement for proofs of the theorems. The two theorems are identical except for the models estimating the causal effects, and so could be combined into a single theorem. It is not clear what is the value of these theorems as they play no further part in the paper. Readers would also benefit from a working definition of “s-recoverable”.
11. L196, 201 the binomial distributed variables are defined as “Binomial” in the first case and “Bernoulli” in the second; please use a consistent term.
12. L202 this text is not needed given the early structural equations and figure 1.
13. L234ff the numbering of selection mechanisms makes the paper harder to read as one has to constantly refer back to these definitions. Why not just say selection by X, G+X, G+X+Y etc?
14. Could you provide a spreadsheet as supplemental material giving the data points for all the figures?
15. L327 please remind the reader that selection was by smoking status.
16. L339 The attenuation may also be because fewer SNPs were used in the smoking sample, 77 rather than 317. So there is more weak instrument bias in the selected sample, which attenuates estimates in two-sample MR. Why did you not use the same SNPs in both analyses? By selecting SNPs separately in the two analyses, winner’s curse also comes into play which created further weak instrument bias. If you used the same SNPs in both analyses, you would be more confident that the attenuation is indeed due to selection bias.
17. L455 ref 16 is mis-formatted, and is now published in Annals of Statistics.

Comments on supplemental text:

18. The two theorems claim s-recoverability. This property has a technical definition but there is no argument that this definition is met. You merely show that the numerators and denominators of each ratio are the same under selection, under the conditions given. This seems to be a sufficient condition, but not necessary: please clarify. Could these results be obtained simply by arguing from the DAG, without the algebra?
19. Top p3, what is \sigma in the first equation? How is the first equation obtained?
20. P3, line starting “On the other hand”, it is stated that E(Y)=0 under two conditions, but this is incorrect. It is the change in E(Y) that is 0.
21. P7, ZEMPA is not a condition on the ratio estimates; it is a condition on the true ratios.
