# Supplementary material for: Impact of nonrandom selection mechanisms on the causal effect estimation for two-sample Mendelian randomization methods
Source: PLoS Genet. 2022 Mar 17;18(3):e1010107. doi: 10.1371/journal.pgen.1010107 (PMC8963545; doi:10.1371/journal.pgen.1010107)
Supplement: Attachment — Submitted filename: Point by point responses to editor and reviewers.docx [file pgen.1010107.s016.docx]

**Point by point responses to editor and reviewers**

We thank the reviewers and the editor for their positive and constructive comments that greatly improved our manuscript. We really appreciate their precious time and great efforts on reviewing the manuscript. We have now carefully addressed all the review comments. Please find below our detailed responses to the comments. In addition, we point out lines and pages numbers in the version of manuscript without highlight.

**Comments from Editor:**

Although the standard of scientific English is generally very good, particularly in Section 3.2 there were sentences that were confusing to me, such as "... there was an evidence that it more likely to be a morning person (chronotype) were associated with a lower odds of smoking cessation. Therefore, the population enriched for smokers is a selected population depends on chronotype." As a result, I find it hard to understand the significance of this application: are you claiming that there have been published errors? Or just the potential for error due to selection? This application was also not mentioned in the Discussion, making its impact unclear. Can you point to any possible or likely errors in published analyses due to the selection effects that you describe?

**Answer:**

Thanks very much for your remarks. Your comments have led to great improvement of our manuscript, and we have carefully revised the manuscript following your suggestions. The aim of this application is to illustrate the huge influence of selection on causal effect estimation. To give readers a more intuitive understanding of this application, we demonstrate the DAGs of selection mechanism in this application (Figure S81 in the S1 File, see below). We have further illustrated this application in the Section Discussion (lines 398-404, page 17).

**Figure S81.** DAG for application

Gibson et al. [1] have found that chronotype was associated with a lower odds of smoking cessation. In our application, chronotype is the exposure of interest, and smoking maybe the S in sample I (Figure S81 in the S1 File). In other words, we restricted the analysis in smokers, that is, conditioning S=1, which depends on the exposure. In this situation, this selection mechanism has a large influence on the causal effect estimation.

In addition, in the Discussion section, we make a short review of relevant selection mechanisms in GWASs and examples corresponding to selection mechanisms in published two-sample MR studies, as well as existing methods to correct for selection in these situations (see Table 1). Because the two samples used in MR analysis are both from GWAS analysis, we only list possible selections in one GWAS sample, including three cases: selection depending on genetic variants, phenotype (exposure X or outcome Y in MR analysis) and both of them. The first case includes case-control study [2], survival bias and disease progression [3], etc. For example, a case-only study is used to explore the causal effect of BMI on breast cancer survival for ER-positive breast cancer under a MR framework [4]; genetic associations of alcohol-related variants with esophageal cancer have been considered separately in non-drinkers, moderate drinkers and heavy drinkers [5], etc. Several methods such as collider-corrected regression [6], inverse probability weighting method [7-9] and bayesian approaches [10-11] can correct selection bias. For the second case, misalignment in time zero [12] is a possible selection depending on genetic variants. For example, e.g. a MR study of the effects of late-life cholesterol levels on dementia risk could be biased if earlier cholesterol levels affect living long enough to be at risk of dementia [12]. Inverse probability weighting method [7-9] and bayesian approaches [10-11] can also correct selection bias. Thirdly, population stratification and dynastic effects [13] may be selected variables, such as individuals recruited in the analysis include siblings, grandparents, uncles/aunts and cousins [13]. Adjusting for the first 10 principle components in GWAS analysis may correct the bias caused by population stratification.

**Reference**

[1] Gibson M, Munafò MR, Taylor AE, Treur JL. Evidence for Genetic Correlations and Bidirectional, Causal Effects Between Smoking and Sleep Behaviors. Nicotine Tob Res. 2019 May 21;21(6):731-738. doi: 10.1093/ntr/nty230. PMID: 30365022; PMCID: PMC6528151.

[2]. Munafò MR, Tilling K, Taylor AE, Evans DM, Davey Smith G. Collider scope: when selection bias can substantially influence observed associations. Int J Epidemiol. 2018 Feb 1;47(1):226-235. doi: 10.1093/ije/dyx206. PMID: 29040562; PMCID: PMC5837306.

[3]. Gkatzionis A, Burgess S. Contextualizing selection bias in Mendelian randomization: how bad is it likely to be? Int J Epidemiol. 2019 Jun 1;48(3):691-701. doi: 10.1093/ije/dyy202. PMID: 30325422; PMCID: PMC6659463.

[4]. Guo Q, Burgess S, Turman C, et al. Body mass index and breast cancer survival: a Mendelian randomization analysis. Int J Epidemiol. 2017;46(6):1814-1822. doi:10.1093/ije/dyx131

[5]. Lewis SJ, Davey Smith G. Alcohol, ALDH2, and esophageal cancer: a meta-analysis which illustrates the potentials and limitations of a Mendelian randomization approach. Cancer Epidemiol Biomarkers Prev 2005;14:1967–71.

[6]. Barry C, Liu J, Richmond R, Rutter MK, Lawlor DA, Dudbridge F, Bowden J. Exploiting collider bias to apply two-sample summary data Mendelian randomization methods to one-sample individual level data. PLoS Genet. 2021 Aug 9;17(8):e1009703. doi: 10.1371/journal.pgen.1009703. PMID: 34370750; PMCID: PMC8376220.

[7]. Hughes RA, Davies NM, Davey Smith G, Tilling K. Selection Bias When Estimating Average Treatment Effects Using One-sample Instrumental Variable Analysis. Epidemiology. 2019 May;30(3):350-357. doi: 10.1097/EDE.0000000000000972. PMID: 30896457; PMCID: PMC6525095.

[8]. Fu M, Bakulski KM, Higgins C, Ware EB. Mendelian Randomization of Dyslipidemia on Cognitive Impairment Among Older Americans. Front Neurol. 2021 Jun 23;12:660212. doi: 10.3389/fneur.2021.660212. PMID: 34248819; PMCID: PMC8260932.

[9]. Swanson SA. A Practical Guide to Selection Bias in Instrumental Variable Analyses. Epidemiology. 2019 May;30(3):345-349. doi: 10.1097/EDE.0000000000000973. PMID: 30896458.

[10]. Wiemann, Paul FV, Nadja Klein, and Thomas Kneib. "Correcting for sample selection bias in Bayesian distributional regression models." Computational Statistics & Data Analysis (2021): 107382.

[11]. Mokatrin L . Bayesian approach for selection bias correction in regression.[D]. The American University. 2011.

[12]. Swanson SA, Tiemeier H, Ikram MA, Hernán MA. Nature as a Trialist?: Deconstructing the Analogy Between Mendelian Randomization and Randomized Trials. Epidemiology. 2017 Sep;28(5):653-659. doi: 10.1097/EDE.0000000000000699. PMID: 28590373; PMCID: PMC5552969.

[13]. Brumpton B, Sanderson E, Heilbron K, et al. Avoiding dynastic, assortative mating, and population stratification biases in Mendelian randomization through within-family analyses. Nat Commun. 2020;11(1):3519. Published 2020 Jul 14. doi:10.1038/s41467-020-17117-4

**Comments from Reviewers:**

The authors consider selection bias in two-sample Mendelian randomization studies, showing unsurprisingly that selection either sample can lead to bias, increased type-1 error and/or loss in power, but also interestingly that selection in the second, outcome sample is the more serious issue. They present two theorems giving sufficient conditions for unbiased estimation in selected samples. They consider a wide range of selection structures, and a good range of currently popular MR methods. This is an interesting study that informs the field.

**Answer:**

Thank you very much. We sincerely appreciate your effort in reviewing our manuscript. Your comments are very constructive and we have revised our manuscript accordingly. Responses to your specific comments are given below.

**Comments on the main text:**

1.	This work is related to the paper of Hartwig et al, Int J Epidemiol 2021, who considered conditioning on covariates rather than sample selection, but which is logically the same problem. Reassuringly, both sets of authors have found that selection in the second sample is the more severe problem. Please discuss the Hartwig et al work.

**Answer:**

Thanks very much for your profound comments. We have read the paper of Hartwig et al [1] carefully and their work is closely related to our study. We discussed the common points between our study and the Hartwig et al work in depth in the Discussion section (lines 372-381, page 16).

Hartwig et al’s work [1] also revealed that the causal structure in the sample II had a larger influence on the causal effect estimation. Their work aimed to assess whether different covariable-adjusted summary associations in two-sample MR could distort causal effect estimation. They found that using covariable-adjusted summary associations might bias MR analyses. Particularly, the presence of an unmeasured confounder between the covariate and the outcome in the sample II would render the covariate a collider. This type of collider bias is called *analytical colliding bias* [2]. Their work is similar as our work’s conclusion in that *S* is also a collider and restricting the analysis on the selected population will lead to another type of collider bias *sampling colliding bias* [2]. Both types of collider bias will distort the true relationship between the collider’s common causes.

**Reference**

[1]. Hartwig FP, Tilling K, Davey Smith G, Lawlor DA, Borges MC. Bias in two-sample Mendelian randomization when using heritable covariable-adjusted summary associations. Int J Epidemiol. 2021 Feb 23:dyaa266. doi: 10.1093/ije/dyaa266. Epub ahead of print. PMID: 33619569.

[2]. Shahar, E. , and D. J. Shahar . Causal Diagrams and Three Pairs of Biases. InTech, 2012.

2.	Readers would benefit from more discussion of actual selection mechanisms, so that they know which situations their studies might be reflective of. Please give a more complete list of relevant mechanisms (case/control studies, disease progression, non-representative cohorts etc, and include those where selection on G may occur, since you consider this later) and review extant methods to correct for selection in these situations.

**Answer:**

Thanks very much for your profound remarks. This really improved our manuscript a lot.

Following your suggestion, in the Discussion section, we make a short review of relevant selection mechanisms in GWASs and examples corresponding to selection mechanisms in published two-sample MR studies, as well as existing methods to correct for selection in these situations (see Table 1). Because the two samples used in MR analysis are both from GWAS analysis, we only list possible selections in one GWAS sample, including three cases: selection depending on genetic variants, phenotype (exposure X or outcome Y in MR analysis) and both of them. The first case includes case-control study [1], survival bias and disease progression [2], etc. For example, a case-only study is used to explore the causal effect of BMI on breast cancer survival for ER-positive breast cancer under a MR framework [3]; genetic associations of alcohol-related variants with esophageal cancer have been considered separately in non-drinkers, moderate drinkers and heavy drinkers [4], etc. Several methods such as collider-corrected regression [5], inverse probability weighting method [6-8] and bayesian approaches [9-10] can correct selection bias. For the second case, misalignment in time zero [11] is a possible selection depending on genetic variants. For example, e.g. a MR study of the effects of late-life cholesterol levels on dementia risk could be biased if earlier cholesterol levels affect living long enough to be at risk of dementia [12]. Inverse probability weighting method [6-8] and bayesian approaches [9-10] can also correct selection bias. Thirdly, population stratification and dynastic effects [12] may be selected variables, such as individuals recruited in the analysis include siblings, grandparents, uncles/aunts and cousins [12]. Adjusting for the first 10 principle components in GWAS analysis may correct the bias caused by population stratification.

**Reference**

[1]. Munafò MR, Tilling K, Taylor AE, Evans DM, Davey Smith G. Collider scope: when selection bias can substantially influence observed associations. Int J Epidemiol. 2018 Feb 1;47(1):226-235. doi: 10.1093/ije/dyx206. PMID: 29040562; PMCID: PMC5837306.

[2]. Gkatzionis A, Burgess S. Contextualizing selection bias in Mendelian randomization: how bad is it likely to be? Int J Epidemiol. 2019 Jun 1;48(3):691-701. doi: 10.1093/ije/dyy202. PMID: 30325422; PMCID: PMC6659463.

[3]. Guo Q, Burgess S, Turman C, et al. Body mass index and breast cancer survival: a Mendelian randomization analysis. Int J Epidemiol. 2017;46(6):1814-1822. doi:10.1093/ije/dyx131

[4]. Lewis SJ, Davey Smith G. Alcohol, ALDH2, and esophageal cancer: a meta-analysis which illustrates the potentials and limitations of a Mendelian randomization approach. Cancer Epidemiol Biomarkers Prev 2005;14:1967–71.

[5]. Barry C, Liu J, Richmond R, Rutter MK, Lawlor DA, Dudbridge F, Bowden J. Exploiting collider bias to apply two-sample summary data Mendelian randomization methods to one-sample individual level data. PLoS Genet. 2021 Aug 9;17(8):e1009703. doi: 10.1371/journal.pgen.1009703. PMID: 34370750; PMCID: PMC8376220.

[6]. Hughes RA, Davies NM, Davey Smith G, Tilling K. Selection Bias When Estimating Average Treatment Effects Using One-sample Instrumental Variable Analysis. Epidemiology. 2019 May;30(3):350-357. doi: 10.1097/EDE.0000000000000972. PMID: 30896457; PMCID: PMC6525095.

[7]. Fu M, Bakulski KM, Higgins C, Ware EB. Mendelian Randomization of Dyslipidemia on Cognitive Impairment Among Older Americans. Front Neurol. 2021 Jun 23;12:660212. doi: 10.3389/fneur.2021.660212. PMID: 34248819; PMCID: PMC8260932.

[8]. Swanson SA. A Practical Guide to Selection Bias in Instrumental Variable Analyses. Epidemiology. 2019 May;30(3):345-349. doi: 10.1097/EDE.0000000000000973. PMID: 30896458.

[9]. Wiemann, Paul FV, Nadja Klein, and Thomas Kneib. "Correcting for sample selection bias in Bayesian distributional regression models." Computational Statistics & Data Analysis (2021): 107382.

[10]. Mokatrin L . Bayesian approach for selection bias correction in regression.[D]. The American University. 2011.

[11]. Swanson SA, Tiemeier H, Ikram MA, Hernán MA. Nature as a Trialist?: Deconstructing the Analogy Between Mendelian Randomization and Randomized Trials. Epidemiology. 2017 Sep;28(5):653-659. doi: 10.1097/EDE.0000000000000699. PMID: 28590373; PMCID: PMC5552969.

[12]. Brumpton B, Sanderson E, Heilbron K, et al. Avoiding dynastic, assortative mating, and population stratification biases in Mendelian randomization through within-family analyses. Nat Commun. 2020;11(1):3519. Published 2020 Jul 14. doi:10.1038/s41467-020-17117-4

3.	L67, refs 5 and 6 are blatant self-references, and not really pertinent to the context. Similarly for ref 7 on L68; however, a relevant reference is needed to back up that statement (eg Paternoster et al 2017, in this journal).

**Answer:**

Thanks for your suggestions. We are sorry for these self-references. We found three other references (listed below) closely related to the context, including Paternoster et al 2017 in this journal. New reference as follows added to the main text are listed below.

**Reference**

[1]. Dimou NL, Tsilidis KK. A Primer in Mendelian Randomization Methodology with a Focus on Utilizing Published Summary Association Data. Methods Mol Biol. 2018;1793:211-230. doi: 10.1007/978-1-4939-7868-7_13. PMID: 29876899.

[2]. Sanderson E, Davey Smith G, Windmeijer F, Bowden J. An examination of multivariable Mendelian randomization in the single-sample and two-sample summary data settings. Int J Epidemiol. 2019 Jun 1;48(3):713-727. doi: 10.1093/ije/dyy262. Erratum in: Int J Epidemiol. 2020 Jun 1;49(3):1057. PMID: 30535378; PMCID: PMC6734942.

[3]. Paternoster L, Tilling K, Davey Smith G. Genetic epidemiology and Mendelian randomization for informing disease therapeutics: Conceptual and methodological challenges. PLoS Genet. 2017 Oct 5;13(10):e1006944. doi: 10.1371/journal.pgen.1006944. PMID: 28981501; PMCID: PMC5628782.

4.	L76-79 I didn’t understand these sentences. What collider is Y a descendent of, when there is a causal effect, but not when there isn’t a causal effect?

**Answer:**

We are sorry for not expressing this sentence clearly. What we want to state is that when there is a causal effect, *Y* is a descendant of collider *X*, selection depending on *Y* will not only open the path *X*→*Y*←*U*, but also open the path *G*→*X*←*U*→*Y*. We have now clarified this in the main text (lines 75-76, page 4).

5.	L108 eqn 1 treats G as a random variable, but it was just defined as a set of random variables.

**Answer:**

Thank you very much. We are sorry for overlooking this mistake. Let $G\text{=\{}G_{1},G_{2},...,G_{J}\text{\}}$ denote *J* genetic variants that are mutually independent, and *X*, *Y* and *U* be the exposure, outcome and unmeasured confounder, respectively. The model underlying Figure 1 should be:

$$\begin{matrix} E[Y|X,U,G_{1},...,G_{J}]=\theta X+U+\sum_{j=1}^{J} \gamma_{j}G_{j} \\ E[X|U,G_{1},...,G_{J}]=\sum_{j=1}^{J} \alpha_{j}G_{j}+U \end{matrix}$$

We have corrected this in the main text (line 107, page 6).

6.	L125 Having just discussed the linearity assumption, it is now odd to see discussion of logistic models and odds ratios.

**Answer:**

Thanks very much for your remarks. We completely agree that it is odd to discuss logistic models and odds ratios after linearity assumption. In order to maintain the consistency of the article, we have kept the Theorem 1 in the main text (lines 137-141, page 7) and moved Theorem 2 to S1 File 2.2.

It is common to perform two-sample MR analysis with binary variables. When exposure and outcome are binary, traditional MR methods can be used to judge whether there is a causal effect, but cannot estimate causal effect accurately. It is necessary to provide sufficient conditions for existence of causal relationship. Therefore, we have provided Theorem 2 and its proof as well as DAGs satisfying condition (a-b) in the Supporting information (S1 File, Section 2.2). We have also clarified the model assumptions for the binary variable setting.

7.	L126 “genetic correlation” is the wrong expression.

**Answer:**

Thank you for pointing it out. We have corrected this as “The genetic association statistics with exposure ($\hat{\beta}_{G_{j}X}$,$j=1,2,...,J$) and outcome ($\hat{\beta}_{G_{j}Y}$,$j=1,2,...,J$) can be obtained from sample I and sample II, respectively.”. (line 124, page 6)

8.	L134 This is the first occurrence of “exchangeability assumption”, previously termed independence assumption. Please use a consistent terminology.

**Answer:**

Thanks very much. We have corrected this.

9.	L138 S1 and S2 have not been properly defined. So far they are just called the “selection mechanism”, but here they seem to be treated as random variables. Why not define random variables taking the value 1 is a subject is selected into the study? Then the “dot” notation in your subscripts, which is non-standard and odd, could be replaced by the conditioning symbol.

**Answer:**

Thanks very much for your profound comments. We highly agree with you points. We have now redefined $S_{1}$ and $S_{2}$ as follows “$S_{1}$ and $S_{2}$ are binary variables indicating whether a participant is selected or unselected in sample I and sample II, respectively. Restricting the analysis to the selected sample implies conditioning on $S_{1}$ or $S_{2}$ equaling to 1, which is represented by a box around $S_{1}$ or $S_{2}$.” (lines 128-129, page 6). In addition, we have changed the dot notation to the conditioning symbol(lines 137-141, page 7).

10.	In the main text, please direct the reader to the supplement for proofs of the theorems. The two theorems are identical except for the models estimating the causal effects, and so could be combined into a single theorem. It is not clear what is the value of these theorems as they play no further part in the paper. Readers would also benefit from a working definition of “s-recoverable”.

**Answer:**

Thank you for your remarks. Following your suggestion, we have now directed the readers to the proof of the theorem as follows “Proof of this theorem is provided in the Supporting information (S1 File, Section 2.1)” (line 147, page 7).

Secondly, the conditions (a) in the two theorems are not exactly the same. The condition (a) in Theorem 1 is “for each valid instrumental variable *G_j_*, $S_{1}\perp G_{j}$or $S_{1}\perp X|G_{j}$in sample I and $S_{2}\perp G_{j}$or $S_{2}\perp Y|G_{j}$ in sample II, respectively”. For Theorem 2, in logistic model, due to non-collapsibility, $S_{1}\perp G_{j}$ and $S_{2}\perp G_{j}$ in condition (a) are changed to $S_{1}\perp G_{j}|X$ and $S_{2}\perp G_{j}|Y$, respectively. Thus the condition (a) in Theorem 2 can be written as “for each valid instrumental variable *G_j_*, $S_{1}\perp G_{j}|X$or $S_{1}\perp X|G_{j}$in sample I and $S_{2}\perp G_{j}|Y$or $S_{2}\perp Y|G_{j}$ in sample II, respectively”. In order to maintain the consistency of the article, we have kept the Theorem 1 in the main text (lines 137-141, page 7) and moved Theorem 2 to S1 File 2.2.

Thirdly, these two theorems provide sufficient conditions for causal effect invariance. We provide corresponding DAGs for these conditions. DAGs satisfying condition (a-b) in **Theorem 1** are listed in Figure 2-3. Three scenarios including no nonrandom selection mechanism, selection depending on unmeasured confounder as well as selection depending on genetic variants (Figure 2), satisfy condition (a) in sample I or sample II. Two scenarios including selection depending on the outcome or genetic variants (Figure 3) in sample II satisfy condition (b). The differences for the DAGs satisfying Theorem 2 are that selection depending on unmeasured confounder no more satisfy the condition (a) in either sample I or sample II. Instead, selection depending on exposure in sample I and outcome in sample II satisfy condition (a) in Theorem 2.

Finally, we agree it is confusing to use “s-recoverable” without a clear definition. We have directly modified it to “The sufficient conditions for causal effect invariance” in Theorem 1, and “sufficient conditions for existence of causal relationship” in Theorem 2.

11.	L196, 201 the binomial distributed variables are defined as “Binomial” in the first case and “Bernoulli” in the second; please use a consistent term.

**Answer:**

Thanks very much. We have corrected this and used “Binomial” through all manuscript.

12.	L202 this text is not needed given the early structural equations and figure 1.

**Answer:**

Thanks very much. We have removed this.

13.	L234 the numbering of selection mechanisms makes the paper harder to read as one has to constantly refer back to these definitions. Why not just say selection by *X, G+X, G+X+Y* etc?

**Answer:**

Thanks very much. We appreciate the concise notation suggested by the reviewer. We have removed the numbering of selection mechanisms throughout the manuscript and used selection by *X, G+X, G+X+Y* instead.

14.	Could you provide a spreadsheet as supplemental material giving the data points for all the figures?

**Answer:** Thanks for your comments. We have provided 4 spreadsheets (Supplementary Table 1-4) as supplemental materials giving the data points for all the figures.

15.	L327 please remind the reader that selection was by smoking status.

**Answer:**

Thanks for your comments. It does need to be emphasized that selection was by smoking status. We have now added the statement that “To examine the bias of the causal effect estimation under nonrandom selection mechanism, we also use a selected sample enriched for smokers with 128,266 individuals as the selected population.” in the Section Methods 2.6 (lines 263-265, page 12). In addition, we restated that “we use the same SNPs in the general population and the selected population, for the latter population we select individuals who are smokers.” in the Section Results. (lines 330-331, page 15)

16.	L339 The attenuation may also be because fewer SNPs were used in the smoking sample, 77 rather than 317. So there is more weak instrument bias in the selected sample, which attenuates estimates in two-sample MR. Why did you not use the same SNPs in both analyses? By selecting SNPs separately in the two analyses, winner’s curse also comes into play which created further weak instrument bias. If you used the same SNPs in both analyses, you would be more confident that the attenuation is indeed due to selection bias.

**Answer:**

Thanks very much for your profound remarks. Yes, using the same SNPs in general and smoking population can avoid the influence of winner’s curse, and we will be more confident that the attenuation is indeed due to selection bias.

To avoid the influence of winner’s curse, we have now used 310 SNPs in both analysis. Figure 11 (new) are results using the same number of SNPs in general and smoking population. We found that the attenuation still exists. Thus we are more confident that the attenuation is indeed due to selection bias.

**Figure 11.** Results of MR analysis of chronotype on the risk of breast cancer.

17.	L455 ref 16 is mis-formatted, and is now published in Annals of Statistics.

**Answer:**

We are sorry for this mistake. We have corrected this.

**Comments on supplemental text:**

18.	The two theorems claim s-recoverability. This property has a technical definition but there is no argument that this definition is met. You merely show that the numerators and denominators of each ratio are the same under selection, under the conditions given. This seems to be a sufficient condition, but not necessary: please clarify. Could these results be obtained simply by arguing from the DAG, without the algebra?

**Answer:** Thanks for your comments.

We have now modified the term “s-recoverable” to “The sufficient conditions for causal effect invariance” in Theorem 1, and “sufficient conditions for existence of causal relationship” in Theorem 2 in S1 File Section 2.2.

Secondly, we also provide DAGs satisfying condition (a-b) in Theorem 1 (Figure 2-3) and Theorem 2 (Figure S1-S2 in S1 File). Three scenarios including no nonrandom selection mechanism, selection depending on unmeasured confounder as well as selection depending on genetic variants (Figure 2), satisfy the condition (a) in sample I or sample II. Two scenarios including selection depending on the outcome or genetic variants (Figure 3) in sample II satisfy the condition (b). The differences for the DAGs satisfying Theorem 2 are that selection depending on unmeasured confounder no more satisfy the condition (a) in either sample I or sample II. Instead, selection depending on exposure in sample I and outcome in sample II satisfy condition (a) in Theorem 2.

Figure 2. The possible causal diagrams for condition (a) of Theorem 1.

Figure 3. The possible causal diagrams for condition (b) of Theorem 1.

Figure S1. The possible causal diagrams for condition (a) of Theorem 2.

Figure S2. The possible causal diagrams for condition (b) of Theorem 2.

19.	Top p3, what is \sigma in the first equation? How is the first equation obtained?

**Answer:** We are sorry for the unclear expressions. $\sigma$ is the correlation coefficient. For example, $\sigma_{GX}$ is the correlation coefficient between $G$ and $X$. However, it is not standard to use $\sigma$ to indicate the correlation coefficient. Therefore, we modified it as $\rho$.

The first equation can be obtained by the following procedure. And we have added it in the supplemental text (S1 File Section 2.1).

For simplicity, each variable is assumed to follow an normal distribution *N*(0,1). The partial regression coefficient $\beta_{GX|S_{1}}$ can be estimated by $\frac{Sd_{X}}{Sd_{G}}\frac{\rho_{GX}-\rho_{XS_{1}}\rho_{GS_{1}}}{1-\rho_{GS_{1}}^{2}}$, where $Sd$ is the standard deviation and $\rho$ is the correlation coefficient. For example, $Sd_{X}$ is the standard deviation of *X* and $\rho_{GX}$ is the correlation coefficient between $G$ and $X$. Because both *X* and *G* follows standard normal distributions, $Sd_{X}=Sd_{G}=1$, i.e. $\beta_{GX|S_{1}}\text{=}\frac{\rho_{GX}-\rho_{XS_{1}}\rho_{GS_{1}}}{1-\rho_{GS_{1}}^{2}}$.

Furthermore, $\rho_{GS_{1}}=\frac{\mathrm{cov}(G,S_{1})}{Sd_{G}Sd_{S_{1}}}=\frac{E(GS_{1})-E(G)E(S_{1})}{Sd_{G}Sd_{S_{1}}}=E(GS_{1})$.

Assume that $E(G)=E(\rho_{GX}X)$and $E(S_{1})=E(\rho_{XS_{1}}X)$,

$E(GS_{1})=E(\rho_{GX}\rho_{XS_{1}}X^{2})=\rho_{GX}\rho_{XS_{1}}E(X^{2})=\rho_{GX}\rho_{XS_{1}}$.

Thus, $\beta_{GX|S_{1}}\text{=}\frac{\rho_{GX}-\rho_{XS_{1}}\rho_{GS_{1}}}{1-\rho_{GS_{1}}^{2}}\text{=}\frac{\rho_{GX}-\rho_{XS_{1}}^{2}\rho_{GX}}{1-\rho_{GX}^{2}\rho_{XS_{1}}^{2}}$.

In addition, $\beta_{GX}=\rho_{GX}$，$\beta_{XS_{1}}=\rho_{XS_{1}}$, $\beta_{GS_{1}}=\rho_{GS_{1}}$due to the normal distributions of $S_{1}$, $G$ and $X$.

Therefore, $\beta_{GX|S_{1}}\text{=}\frac{\rho_{GX}-\rho_{XS_{1}}\rho_{GS_{1}}}{1-\rho_{GS_{1}}^{2}}=\frac{\beta_{GX}-\beta_{XS_{1}}^{2}\beta_{GX}}{1-\beta_{GX}^{2}\beta_{XS_{1}}^{2}}\text{=}\frac{\beta_{GX}(1-\beta_{XS_{1}}^{2})}{1-\beta_{GX}^{2}\beta_{XS_{1}}^{2}}$.

20.	P3, line starting “On the other hand”, it is stated that E(Y)=0 under two conditions, but this is incorrect. It is the change in E(Y) that is 0.

**Answer:** We are sorry for this mistake. We have corrected this as follows.

If $G\perp Y|S_{2}$, we have $\frac{\partial E(Y|G,S_{2}=1)}{\partial G}\text{=}0$. If $G\perp Y$, then $\frac{\partial E(Y|G)}{\partial G}\text{=}0$.

21.	P7, ZEMPA is not a condition on the ratio estimates; it is a condition on the true ratios.

**Answer:** We are sorry for this mistake. Following the paper of Hartwig et al [1], We have corrected it.

[1]. Hartwig FP, Davey Smith G, Bowden J. Robust inference in summary data Mendelian randomization via the zero modal pleiotropy assumption. Int J Epidemiol. 2017 Dec 1;46(6):1985-1998. doi: 10.1093/ije/dyx102. PMID: 29040600; PMCID: PMC5837715.
