# Supplementary material for: Impact of nonrandom selection mechanisms on the causal effect estimation for two-sample Mendelian randomization methods
Source: PLoS Genet. 2022 Mar 17;18(3):e1010107. doi: 10.1371/journal.pgen.1010107 (PMC8963545; doi:10.1371/journal.pgen.1010107)
Supplement: Attachment — Submitted filename: Point_by_point_responses_to_the_reviewer-20220112.docx [file pgen.1010107.s017.docx]

**Reviewer’s Responses to Questions**

We would like to thank the reviewer and Editor for their positive and constructive comments, which greatly improved our manuscript. We appreciate their valuable time and great effort in reviewing the manuscript. We have carefully addressed all the review comments. Please find our detailed responses to their comments below. In addition, we point out the line and page numbers in the version of the manuscript without highlight.

Reviewer #1: The authors have responded to my comments and improved the paper. I have some further comments arising.

**Answer:**

Thank you very much. Your comments were very constructive, and we have revised our manuscript accordingly. The responses to your specific comments are provided below.

1. Table 1. You mention collider-corrected regression as a method to correct for selection bias, but it is not.

**Answer:**

We apologize for this mistake. Collider-corrected regression is a method to correct collider bias between genetic instruments and unmeasured confounders owing to conditioning on the observed exposure. It cannot be used to correct for selection bias. We have deleted this sentence (Refer Table 1).

2. Table 1. it is not clear how population stratification and dynastic effects lead to selection bias. They are well known as confounders of genetic associations, but selection effects are not so clear; please explain.

**Answer:**

We are sorry for this mistake. Population stratification is actually the confounder between the genotype and phenotype. The diverse ancestral origins of many human populations can lead to systematic allele frequency differences across the study populations [1]. The dynastic effect also cannot lead to selection bias. The dynastic effect is an indirect effect of the parent’s genotype on their offspring that is mediated via the parents’ phenotype, i.e. effects that are not mediated via the direct inheritance of DNA [1]. It is also a confounder in SNP–phenotype association. We think “restrict population by specific features” is a relevant selection mechanism in GWASs corresponding to the third DAG in Table 1. For example, in the following figure, if genotype and maternal folate status affect embryo failure, selection bias will occur in the population with embryo failure. Spurious associations between genetic variants and folate levels will lead to biased MR estimates [1, 2].

This figure adapted from Neil D, et al. (2019) [1].

**Reference**

[1].	Davies NM, Howe LJ, Brumpton B, Havdahl A, Evans DM, Davey Smith G. Within family Mendelian randomization studies. Hum Mol Genet. 2019;28: R170–R179. doi: [10.1093/hmg/ddz204](https://doi.org/10.1093/hmg/ddz204).

[2].	Nadeau JH. Do gametes woo? Evidence of nonrandom union at fertilization Genetics. 2017;207: 369–387. doi: [10.1534/genetics.117.300109](https://doi.org/10.1534/genetics.117.300109).

3. Table 1 row 1 col 3. ref 48 does not use inverse probability weighting.

**Answer:**

Thank you for pointing this out to us. We found two other references (listed below) that were closely related to inverse probability weighting. The new references added to the main text are listed below (Refer Table 1).

**References**

[1].	Canan C, Lesko C, Lau B. Instrumental variable analyses and selection bias. Epidemiology. 2017;28: 396-398. doi: [10.1097/EDE.0000000000000639](https://doi.org/10.1097/ede.0000000000000639).

[2].	Gkatzionis A, Burgess S. Contextualizing selection bias in Mendelian randomization: how bad is it likely to be? Int J Epidemiol. 2019;48: 691-701. doi: [10.1093/ije/dyy202](https://doi.org/10.1093/ije/dyy202), PMID: [30325422](http://www.ncbi.nlm.nih.gov/pubmed/30325422), PMCID: [PMC6659463](https://www.ncbi.nlm.nih.gov/pmc/articles/PMC6659463).

4. The author summary could be improved, it is not really at a lay level, X Y and G are undefined, etc.

**Answer:**

Thank you for your remarks. We have modified it as follows.

It is well known that nonrandom selection in one-sample Mendelian Randomization (MR) can result in biased estimates and inflated type I error rates. Actually, two-sample MR analyses are more prone to be affected by nonrandom selection than one-sample MR analyses, because two samples for genome-wide association studies (GWAS) may be selected each under different mechanisms from the source population. Summary-level genetic association statistics in two-sample MR may be derived from different study designs such as case-control, case-only and cohort studies, which further inevitably affect the causal effect estimation of exposure on outcome. In this study, we firstly propose a theorem for causal effect invariance under different selection mechanisms. In the simulation, we design 49 combinations of nonrandom selection mechanisms in sample I and sample II, which are widespread in practical applications. The simulation results reveal that the selection mechanisms in sample II have a larger influence on biases and type I error rates than those in sample I. As an illustrative example, we find the nonrandom selection in sample II (coronary heart disease patients) can magnify the causal effect estimation of obesity on the HbA1c levels.

5. Page 5 line 92. "(pleiotropy) always leads to the violation..." is incorrect, it can do, but not always.

**Answer:**

Thank you very much. We have modified " always " as " can ". The revised sentence is on the page 5, line 96)

6. Equation 1 and Figure 1. Why are gamma and phi both shown by grey dotted lines in the figure, but only gamma appears in the equation?

**Answer:**

Thank you for your comments. For a consistent expression between Equation 1 and Figure 1. Referring to several two-sample MR articles, we have modified Figure 1. The new Figure is as follows:

**Figure 1.** Illustrative diagram of Mendelian randomization.

[1] Jack Bowden, George Davey Smith, Stephen Burgess, Mendelian randomization with invalid instruments: Effect estimation and bias detection through Egger regression, International Journal of Epidemiology, Volume 44 2015, Pages 512–525, https://doi.org/10.1093/ije/dyv080

[2] Bowden J and Holmes MV. Meta-analysis and Mendelian randomization: a review. Res Synth Methods. 2019;10(4):486-496. doi:[10.1002/jrsm.1346](https://doi.org/10.1002/jrsm.1346)

[3] Barry, C., Liu, J., Richmond, R., Rutter, M. K., Lawlor, D. A., Dudbridge, F., Bowden, J. 2021. Exploiting collider bias to apply two-sample summary data Mendelian randomization methods to one-sample individual-level data PLoS genetics, 17(8), e1009703. https://doi.org/10.1371/journal.pgen.1009703

7. Page 8 line 157. "Existence of causal relationship" looks wrong, I think you mean something like invariance of causal relationship.

**Answer:**

Thank you for pointing it out. We have modified " Existence of causal relationship " to " the invariance of causal relationship ". The revised sentence is on page 8 lines 161.

8. Page 8 line 174. IVW is undefined at this point. Not clear what bias you are referring to.

**Answer:**

Thanks for your comments. We have defined "Inverse-variance weighting (IVW)" for the first time on page 7, lines 153.

In this section, we want to express that the nonrandom selection will lead to the violation of the MR assumptions (IV Independence and Exclusion restriction), and further make the instrumental variables invalid. We have modified it as “This directly results in the violation of the MR assumption and further make the instrumental variables invalid.”

9. Page 11 line 232. "Ratio" should be "proportion".

**Answer:**

Thanks very much. We have modified "Ratio" as "proportion" (lines 236, page 11).

10. Page 12 lines 250-254. The discussion of circadian rhythm relating to chemotherapy is distracting from the main question of chronotype as a cause of breast cancer. Not clear that this is necessary.

**Answer:**

Thank you very much for your insightful comment. We reanalyzed a more appropriate application example. The discussion on chronotypes and breast cancer has been deleted. The new application explores whether the causal estimation of obesity on HbA1c is different in patients with CHD and the general population. The results demonstrate that nonrandom selection in sample II (CHD patients) can magnify the causal effect of obesity on HbA1c levels. Fig. S81 shows a realistic causal diagram of this application. Fig. S81(A) shows the DAG for sample I. Fig. S81(B) and (C) are the DAGs for sample II in the general population and CHD patients, respectively.

The revised version for this application in Discussion section are shown on page 17, line 398-404. In our application, we restrict analysis to CHD patients to reveal the significant influence of nonrandom selection on the causal effect estimation of obesity on HbA1c levels. Several MR studies have found that obesity and HbA1c levels play important roles in CHD prevention. HbA1c is the outcome of interest and CHD is the S in sample II (Fig S81 in the S1 File). In other words, we restrict the analysis to CHD patients, that is, conditioning S=1, which depends on the outcome and exposure. In this situation, this selection mechanism can magnify the causal effect estimation of obesity on HbA1c levels.

**Figure S81.** The realistic causal diagram for the application.

11. Page 15 line 326. "To avoid the influence of winner's curse" is poor wording - it is still possible among these selected SNPs. The point in the earlier review was that winner's curse effects would be different between two analyses using different numbers of SNPs. This clause could be deleted without detriment.

**Answer:**

Thank you for your comment. We have deleted this sentence.

12. The smoking example is unconvincing. You claim that smoking is a descendant of the collider at chronotype. But there is no evidence that chronotype causes smoking. It may be smoking that causes chronotype, or their association could be due to confounding. Furthermore there may simply be an effect modification such that the chronotype-breast cancer association is different for smokers. The attenuated associations in the smokers are consistent with selection bias, but are not evidence for it. Finally the association reported by Gibson et al was with smoking cessation, not smoking itself.

**Answer:**

Thank you very much for your comment. It is really less rigorous to use smoking as an example. Instead, we used a more convincing example. We aimed to explore whether the causal estimation of obesity (BMI, WHR, and WHRadjBMI) on HbA1c levels is different between CHD patients and the general population. Several MR studies have found that obesity and HbA1c levels play important roles in CHD prevention. HbA1c is the outcome of interest and S is a binary variable indicating whether a participant is a CHD patient in sample II (Fig S81 in the S1 File).

We used GWAS summary data on BMI, WHR and WHRadjBMI for European descent from the Genetic Investigation of ANthropometric Traits (GIANT) by GWAS meta-analyses of 339,224 and 224,459 individuals, respectively. We retrieved individual data for HbA1c from the UK Biobank with a sample of 487,314 Europeans. To examine the bias of the causal effect estimation under a nonrandom selection mechanism, we also used a selected sample enriched for CHD patients with 26,765 individuals as the selected population.

The results are shown in Fig. 11. In the general population, there are strong evidences for positive causal associations of BMI, WHR, and WHRadjBMI on HbA1c levels. This means that a high BMI and WHR can improve HbA1c levels. All eight MR methods demonstrated consistent results. The effect estimates were magnified in patients with CHD. This verifies that the nonrandom selection in sample II biased the effect estimation.

**Figure 11.** Results of MR analysis of obesity on the risk of HbA1c levels. Three columns from left to right represent MR results of body mass index (BMI), waist-to-hip ratio (WHR) and WHR adjusted for BMI (WHRadjBMI) on HbA1c levels, respectively. The red and blue nodes represent MR analysis in general population and coronary heart disease (CHD) patients, respectively.
